# Exercise contagion in a global social network

**DOI:** 10.1038/ncomms14753

**Published:** 2017-04-18

**Authors:** Sinan Aral, Christos Nicolaides

**Affiliations:** 1 MIT Sloan School of Management, 100 Main Street, E62-364, Cambridge, Massachusetts 02142, USA

## Abstract

We leveraged exogenous variation in weather patterns across geographies to identify social contagion in exercise behaviours across a global social network. We estimated these contagion effects by combining daily global weather data, which creates exogenous variation in running among friends, with data on the network ties and daily exercise patterns of ∼1.1M individuals who ran over 350M km in a global social network over 5 years. Here we show that exercise is socially contagious and that its contagiousness varies with the relative activity of and gender relationships between friends. Less active runners influence more active runners, but not the reverse. Both men and women influence men, while only women influence other women. While the Embeddedness and Structural Diversity theories of social contagion explain the influence effects we observe, the Complex Contagion theory does not. These results suggest interventions that account for social contagion will spread behaviour change more effectively.

## Abstract (web summary)

Some argue that health-related behaviours, such as obesity, are contagious, but empirical evidence of health contagion remains inconclusive. Here, using a large scale quasi-experiment in a global network of runners, Aral and Nicolaides show that this type of contagion exists in fitness behaviours.

Disciplines as diverse as economics^1^, sociology^2^, medicine^3^, computer science^4^, political science^5^ and physics^6^ have recently become interested in the interdependence of behaviours across the human social network. In particular, scientists have begun to ask whether our health^7^ and other behaviours^8^ are contagious, in that our decisions and actions affect the decisions and actions of our peers. If behavioural contagions exist, understanding how, when and to what extent they manifest in different behaviours will enable us to transition from independent intervention strategies to more effective interdependent interventions that incorporate individuals' social contexts into their treatments^9^. Although this line of inquiry has tremendous potential to improve social, economic and business policy, its scientific advancement has been hindered by three distinct empirical challenges.

First, although correlations in the behaviours and outcomes of socially connected individuals are ubiquitous, causal social influence effects are harder to identify. Early work demonstrated correlations in human behaviour in network space and time^3^,^7^,^8^, signaling the possibility that health behaviours cascade through social interactions. But, subsequent investigations revealed multiple statistical challenges to identifying causal peer effects in networks, including homophily (the tendency for individuals to choose similar friends^10^,^11^), confounding effects (the tendency for connected individuals to be exposed to the same external stimuli), simultaneity (the tendency for connected individuals to co-influence each other) and other factors^12^,^13^,^14^. Recent work has addressed some of these challenges by developing new observational^10^ and experimental^15^,^16^,^17^,^18^,^19^ techniques. However, observational techniques struggle to overcome the confounding effects of unobservable factors^20^, while experimental studies, which provide more robust causal inference, are complex, difficult to implement and therefore more rare. To scale up scientific investigations of peer effects, we advocate for the exploitation of naturally occurring (rather than experimentally created) random variation across network ties to identify causal social influence. The generalization of such methods to the study of peer effects could not only identify causal peer influence across behaviours but also extend the effectiveness of causal inference in networked studies beyond strictly experimental settings.

Second, studies of social contagion currently suffer from substantial measurement error. On the one hand, survey-based studies, which elicit data about meaningful offline health behaviours such as smoking, obesity or happiness, rely on infrequent and often inaccurate^21^,^22^ self-reports of behaviours and outcomes^3^,^7^,^8^. On the other hand, experiments, which are easier to conduct digitally, are almost exclusively applied to less tangible and less potentially meaningful online behaviours, such as the adoption and use of social applications^17^,^18^, clicking on social advertisements^23^, the virality of internet memes^24^ or the use of positive or negative emotive language in digital status updates^25^. These behaviours may not proxy well for the more tangible, costly, offline health behaviours that meaningfully impact public health. Between these two extremes lies an important alternative approach that aims to provide precise, granular measurement, not of digital behaviours such as clicks or shares but of more consequential, offline health behaviours, such as diet or exercise. The coming wave of quantified self and fitness tracking data, of the type we employ here, collected by wearable devices that record detailed exercise activities time stamped to the second, will likely advance and accelerate the effectiveness of this alternative approach dramatically.

Third, current causal social influence research has limited generalizability. While field experiments have taught us much about the foundations of population-scale peer effects and their consequences, they constrain us to focus on behaviours we can easily randomize, such as the receipt of digital notifications^17^,^18^,^19^ or the social information contained in display advertisements^23^, limiting our scope of inquiry to a small set of specific, narrow conditions and behaviours. Increased experimental control in the laboratory, on the other hand, enables tests of conditions that are difficult to manipulate in the real world, such as the network structure in which individuals are embedded^16^,^26^. But, it is unknown whether these results generalize because the relationships that individuals are randomly assigned to in the laboratory are typically artificial. If the study of social influence is to impact public health, we must overcome these limitations by examining generalized peer effects, such as the effect of individuals' overall exercise behaviours on their friends, in data on actual exercise behaviours and real relationships interacting in their natural states. It is in precisely these settings that experimentation is hardest.

Our analysis of the precisely recorded daily exercise patterns of over a million people who ran over 350 million (M) km in a global social network of runners over 5 years showed that exercise is socially contagious and that its contagiousness varies with the relative activity levels of and gender relationships between friends. Less active runners influence more active runners, while the reverse is not true. Both men and women influence men, while only women influence other women. While the Embeddedness and Structural Diversity theories of social contagion explain the influence effects we observed, evidence for the Complex Contagion theory is mixed.

## Results

### Naive contagion estimates

We estimated social contagion in the exercise behaviours of runners worldwide in a data set that precisely records the geographic locations, social network ties and daily running patterns of ∼1.1M individuals, who ran ∼359M km in a global social network of runners over 5 years. Following Aral^12^, we define the magnitude of peer effects or contagion in exercise behaviour (which we also refer to as social influence, social contagion, behavioural contagion and network contagion) as the degree to which the exercise behaviours of one's peers change the likelihood that or extent to which one engages in those behaviours. The data contain the daily distance, duration and pace of, as well as calories burned during, runs undertaken by these individuals, as recorded by a suite of digital fitness-tracking devices. The data also track ∼3.4M social network ties formed among runners to connect and keep track of each other's running behaviours. We analyse the ∼2.1M ties in the network for which we can geographically locate and find weather information for both nodes connected by a tie. Ties in this network link runners who follow each other's running habits. Running information was not self-reported. When a run was completed, it was immediately digitally shared with a runner's friends. Runners could not choose which runs they shared but rather comprehensively shared all new running information with their friends upon connecting their device to the platform.

These data give us unique insight into the daily, coevolving running and social network patterns of these individuals over 5 years. For example, when we examined progressively more sophisticated models of the correlations between an individual's (also called ego's) running behaviour and that of his or her friends (also called peers) (we use the terms friends and peers interchangeably throughout the paper), we found strong evidence of the possibility of social contagion in running behaviours in both model-free correlations and ordinary least squares (OLS) models that control for time invariant and time varying characteristics of individuals and their peers, including gender, height, weight, degree, device type and country. In the OLS models, an additional kilometre run by peers was associated with an additional 6/10th of a kilometre run by ego and an additional 10 min run by peers was associated with an additional 5.3 min run by ego (see ‘Comparison of IV Estimates with an OLS Model' in Supplementary Note 3 for more detail).

Unfortunately, these estimates are only suggestive because they are subject to the well-known endogeneity biases created by homophily, confounding effects, simultaneity and other factors. We therefore focus our analysis on a natural experiment created by exogenous variation in global weather patterns across geographies. Our approach leverages an inference technique called the instrumental variables (IV) framework, which disentangles endogeneity by using exogenous variation created by natural events as a shock to one endogenous variable to estimate its causal effect on another variable (see the Methods section for more detail).

### IV estimation

**Figure 1:**
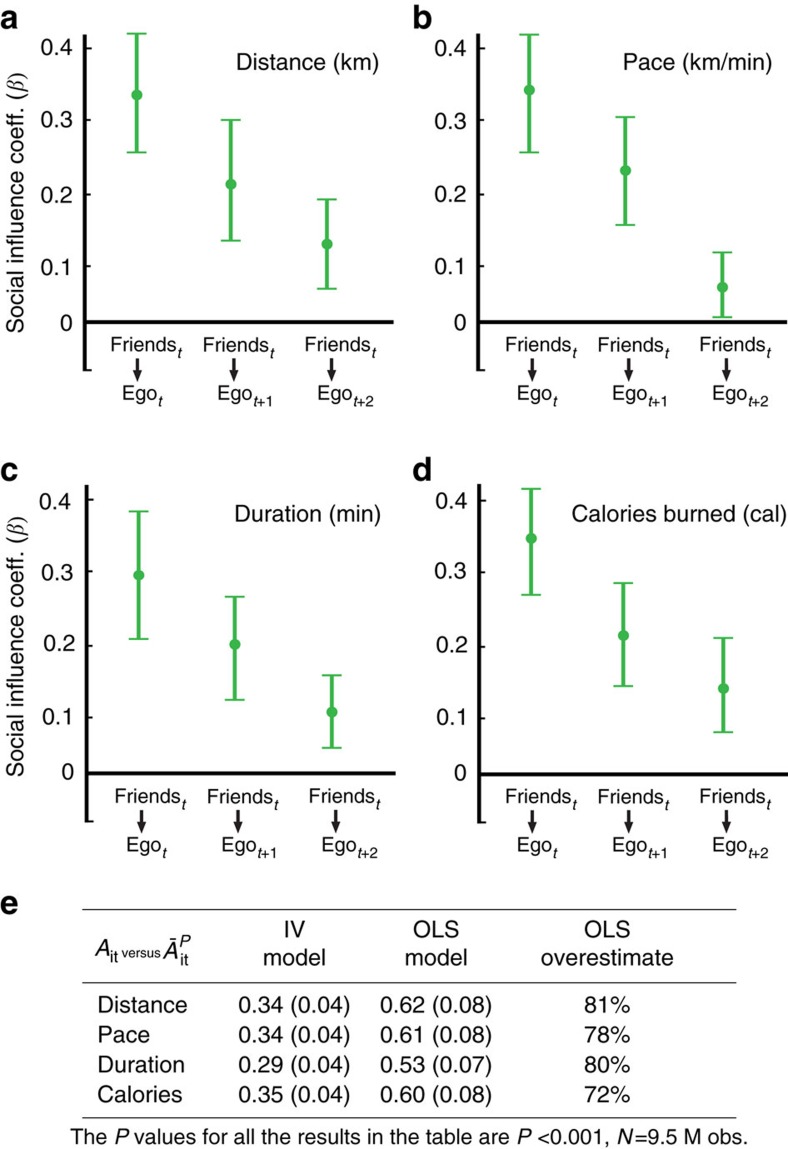
Peer effects in global running behaviours. The panels display social influence coefficients from second-stage regressions in the two-stage least squares specification for friends' behaviour at time *t* influencing ego at time *t*, *t*+1 and *t*+2 for (**a**) distance ran in kilometres (km), (**b**) pace in km per minute, (**c**) running duration in minutes and (**d**) calories burned. Bars are 95% confidence intervals. (**e**) The table at the bottom of the figure compares social influence coefficients and s.e. from the IV models to those from the OLS models and provides the OLS overestimates of social influence as a percentage of the IV estimates.

The results of our IV analysis revealed strong contagion effects: on the same day, on average, an additional kilometre run by friends influences ego to run an additional 3/10th of a kilometre (Fig. 1a); an additional kilometre per minute run by friends influences ego to run an additional 3/10th of a kilometre per minute faster (Fig. 1b); an additional 10 min run by friends influences ego to run 3 min longer (Fig. 1c); and an additional 10 calories burned by friends influences ego to burn three and a half additional calories (Fig. 1d). This peer influence diminishes over time, with friends' running today influencing ego less tomorrow and the day after for every measure.

Peer effects in exercise behaviours are both statistically and socially significant. Suppose, for example, that a runner (A) usually runs 6 km at a pace of 7 min km^−1^ (0.143 km min^−1^) and their friend (B) usually runs 6 km at a pace of 8 min km^−1^ (0.125 km min^−1^). An extra kilometre run by B (an increase from 6 to 7 km) causes A to increase their running distance by 0.3 km (from 6 to 6.3 km). Also, a 0.01 km min^−1^ increase in runner B's pace (from 0.125 to 0.135 km min^−1^) causes runner A to increase their pace by 0.003 km min^−1^ (from 0.143 to 0.146 km min^−1^).

The results in Fig. 1 also summarize the dangers of model misspecification in the estimation of peer effects. Naive models that do not account for endogeneity biases created by homophily, confounding effects, simultaneity and other factors dramatically overestimate social spillovers. As the table in Fig. 1e shows, OLS models that control for ego's (*X*_*it*_) and peers' 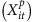 time varying and time invariant characteristics (including age, gender, height, weight, degree, device type and country) but that do not implement the IV identification strategy overestimate social influence by between 72% and 81%.

### Contagion heterogeneity

**Figure 2:**
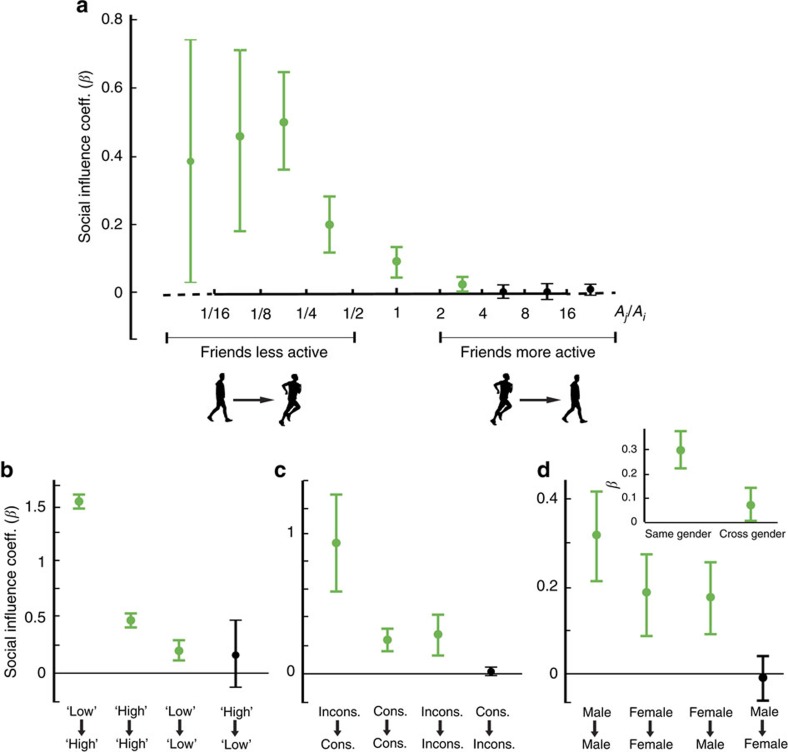
Heterogeneity in social influence effects across relationships. The panels display social influence coefficients across dyadic relationships in which ego is (**a**,**b**) a more or less active runner than their friends, (**c**) a more or less consistent runner than their friends and (**d**) either the same or a different gender than their friends. Bars are 95% confidence intervals.

Peer effects in running are also heterogeneous across relationship types. For example, runners are more influenced by peers whose performance is slightly worse, but not far worse, than their own as well as by those who perform slightly better, but not far better, than they do (Fig. 2a). Moreover, less active runners influence more active runners more than more active runners influence less active runners (Fig. 2b). These results are corroborated by heterogeneity across consistent and inconsistent runners. Inconsistent runners influence consistent runners more than consistent runners influence inconsistent runners (Fig. 2c). Social comparisons may provide an explanation for these results. Festinger's social comparison theory proposes that we self-evaluate by comparing ourselves to others^27^. But, in the context of exercise, a debate exists about whether we make upward comparisons to those performing better than ourselves^28^ or downward comparisons to those performing worse than ourselves^29^. Comparisons to those ahead of us may motivate our own self-improvement, while comparisons to those behind us may create ‘competitive behaviour to protect one's superiority' (27, p. 126). Our findings are consistent with both arguments, but the effects are much larger for downward comparisons than for upward comparisons.

We also found strong evidence that social influence depends on gender relations. Influence among same sex pairs is strong, while influence among mixed sex pairs is statistically significantly weaker (Fig. 2d inset). Men strongly influence men, and women moderately influence both men and women. But, men do not influence women at all (Fig. 2d). This may be due to gender differences in the motivations for exercise and competition. For example, men report receiving and being more influenced by social support in their decision to adopt exercise behaviours, while women report being more motivated by self-regulation and individual planning^30^. Moreover, men may be more competitive and specifically more competitive with each other. Experimental evidence suggests that women perform less well in mixed gender competition than men, even though they perform equally well in non-competitive or single sex competitive settings^31^.

### Testing structural theories of contagion

Finally, three theories describe how social network structure may shape behavioural contagions. Centola and Macy^32^ argue that complex contagions, involving costly behaviours, require multiple reinforcing signals of adoption from different peers to induce behaviour change and suggest that clustered social networks are therefore more likely to spread a complex contagion from one neighborhood to another. Centola^16^ goes on to predict that in real-world health behaviours such as exercise, which are more costly in terms of ‘time, deprivation, or even physical pain', the need for social reinforcement should be greater than in his own study of less costly online health behaviours. In contrast, Ugander *et al*.^33^ suggest that structural diversity, measured by the number of unconnected clusters (called ‘components') with at least one adopter, not the number of distinct peers, is the critical structural factor moderating influence. Aral and Walker^34^, on the other hand, suggest that embeddedness (the number of mutual connections), rather than the number of unconnected clusters, is what drives behavioural contagions. We tested these three structural theories of social contagion by examining how contagion in running varied across different network structures (see ‘Testing Structural Theories of Social Contagion' section in Supplementary Note 2 and ‘Structural Theories of Social Contagion' in Supplementary Note 3 for details).

**Figure 3:**
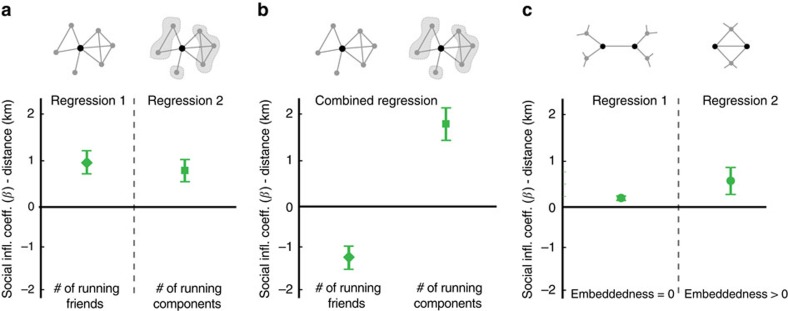
Testing structural theories of networked contagion. The panels describe the structural correlates of social influence in the distance run (in km). Panel (**a**) estimates the social influence effects of the number of distinct friends that run and the number of distinct components of friends that run independently, in separate regressions (separated by the dotted line). Panel (**b**) directly compares, in the same regression, the number of distinct friends that run (supporting Complex Contagion theory) and the number of distinct network components of friends that run (supporting Structural Diversity theory) as structural moderators of social influence effects. The positive estimate for the number of distinct network components of friends that run and the negative estimate for the number of distinct friends that run, when both are analysed together in (**b**), supports the Structural Diversity theory. Panel (**c**) tests whether embedded dyadic relationships with mutual friends transmit influence more effectively than relationships with no mutual friends (supporting Embeddedness theory). The social influence coefficient estimated for embedded relationships (Regression 2) is statistically significantly greater than the social influence coefficient estimated for non-embedded relationships (Regression 1) (*t*-statistic=2.45, *N*=10.7M). Bars are 95% confidence intervals.

We found strong evidence confirming both the Structural Diversity and Embeddedness theories of social contagion, but the evidence for Complex Contagion was mixed. Social influence coefficients under the Complex Contagion theory (which argues that the number of active friends is the key driver of diffusion for complex contagions) and the Structural Diversity theory (which argues that the number of active network components is the key driver of diffusion) are statistically significantly different (*t*-statistic=15.9, *N*=9.9M). The number of distinct friends who run is positively correlated with social influence when analysed alone (Fig. 3a), but this correlation disappears and becomes negative when we control for the structural diversity of the behaviourally active peer group (Fig. 3b). At the same time, the structural diversity of peer group activation (the number of unconnected network components that exhibit running) strongly predicts greater positive social contagion effects, even when we control for the number of distinct friends who run (Fig. 3b). This replicates the results of Ugander *et al*.^33^, who found that, for the social diffusion of Facebook, the number of active friends predicts Facebook adoption but that this correlation disappears and becomes negative when controlling for the structural diversity of Facebook adopting friends. We describe the evidence for Complex Contagion as mixed because the theory defines a complex contagion as one that exhibits adoption thresholds greater than one, meaning more than one adopter friend is required for transmission, and suggests that clustering in behavioural adoption is more conducive to the spread of complex contagions. Our findings show that contagion occurs even with only one adopter friend and that unconnected adopter friends, rather than connected adopter friends, are more likely to transmit exercise behaviours. These results suggest that exercise is not a complex contagion, but they do not invalidate Complex Contagion theory as other behaviours may indeed exhibit complex contagion dynamics.

The data also confirm that the embeddedness of a relationship (the number of mutual friends between contacts) strongly moderates social influence and contagion in running behaviours (Fig. 3c), confirming the Embeddedness theory. Unlike Complex Contagion and Structural Diversity, the Embeddedness theory does not make predictions about the social structure of adopting friends but rather about the social structure surrounding a transmission, whether or not that structure contains other adopting friends. The embeddedness of a relationship, measured by the number of mutual friends a dyad shares, can promote behavioural contagion because of the social monitoring that embedded relationships facilitate. When two people have many mutual friends, there are greater opportunities for social sanctions, reputational consequences for misbehaviour and social rewards for positive behaviours. Mutual friends may therefore provide an added incentive to keep up with running buddies because shirking is widely observed in a set of mutually reinforcing relationships.

## Discussion

Scientists have recently made great strides in understanding social contagion using longitudinal surveys and narrowly designed digital experiments. But, if we are to develop a robust, generalizable and precisely measured understanding of human health interdependence, we must pursue an alternative approach that examines generalized peer effects in data on actual behaviours and real relationships interacting in their natural states. Our work takes this approach to estimate social contagion in exercise behaviour by examining detailed, daily exercise behaviours and social network ties among ∼1.1M runners worldwide. We found that exercise is socially contagious, revealing a behavioural mechanism that could explain the correlations in obesity and happiness found in earlier work^7^,^8^. These results suggest that social intervention strategies, which account for peer effects, may spread behaviour change in networks more effectively than policies that ignore social spillovers^9^. The work also implies several avenues for future research.

First, the granularity and precision with which fitness tracking devices record real-world health behaviours portends a sea change in our understanding of human behaviour and social influence at scale. Compared with prior studies, which relied on imprecise and frequently inaccurate self-reports, the potential for these kinds of data to extend our understanding of social behaviour in real-world settings is difficult to overstate. Although there are limitations to the use of these kinds of data, in many respects they enable significant advances in the fidelity of observation and therefore the precision of the science.

Second, the analysis of heterogeneous treatment effects suggests the broad importance of not focussing exclusively on average social effects. Different subsegments of the population react differently to social influence. Such differences suggest that policies tailored for different types of people in different subpopulations will be more effective than policies constructed with only average treatment effects in mind. In fact, if subpopulations experience countervailing treatment effects, then average treatment effects may be zero even though different people are experiencing strong and significant social effects in opposite directions.

Third, the work points to the importance of examining theories of social contagion in real-world settings. Although laboratory experiments are instrumental to our understanding of social phenomena and help us reason about what types of effects are possible, people may not behave the same way in naturalistic settings as they do in the laboratory. It is therefore important to empirically examine competing theories of social contagion in the field. Such work is essential, not just in testing the validity of the theory in the real world but also in obtaining precise estimates of social contagion that provide more realistic projections of the outcomes of social and behavioural policy interventions.

## Methods

### IV framework

We estimated social contagion in exercise behaviours and avoided well-known empirical challenges in estimating causal peer effects by combining the running and social network data of ∼1.1M individuals who ran over 350M km in a global social network of runners over 5 years with records of the daily global temperature and precipitation patterns experienced by these same individuals over time, recorded by over 47,000 weather stations in 196 countries. Similar to natural experiments^35^, our approach leverages an inference technique developed by applied econometricians to identify causal effects across a variety of phenomena, including the impact of income on civil conflict^36^, poverty on crime^37^ and riots on labour markets^38^. This technique, called the IV framework, disentangles endogeneity by using variation created by exogenous events as a shock to one endogenous variable to estimate its causal effect on another variable^39^.

For example, Angrist^40^ uses random variation in the likelihood of military service created by the draft lottery to identify the causal effect of military service on wages. Since individuals with lower expected wages are more likely to choose to serve in the military, estimating the raw correlation between military service and wages produces a biased estimate of the causal effect. However, since the draft lottery is randomized and therefore uncorrelated with wages, an individual's draft lottery number can be used to identify the causal effect of military service on future earnings. Military service is first regressed on randomly assigned draft lottery numbers. Then, future wages are regressed on the predicted values of military service from this first-stage regression. The draft lottery affects the likelihood of military service because one's lottery number determines whether one is drafted. But, the lottery is uncorrelated with past wages and future wage potential (except through their impact on the likelihood of military service) because lottery numbers are randomly assigned. Since the military service driven by the draft lottery is unrelated to the future wage potential of those who serve, unbiased estimates of the average causal effect of military service on wages can be established by examining the effect of military service mandated by the draft lottery on the future wages of those who were randomly selected to serve.

To adapt the IV framework to the network setting, we need to identify a naturally occurring source of variation in individuals' running behaviour, which is exogenous to, or uncorrelated with, the behaviour of their peers. For our purposes, the weather is an ideal instrument^41^. As social ties span geographies, our data record many relationships in which peers experience uncorrelated weather. In these relationships, the weather experienced by one person is an excellent source of exogenous variation that perturbs their running behaviour without affecting the running behaviour of their geographically distant friends. We can then estimate causal social influence effects in running behaviour in a two-stage least squares specification, using the uncorrelated weather experienced by peers as an instrument for identifying the social influence they exert on ego. We specified our model of individual-level peer effects as follows:


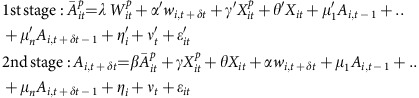


In the first stage, the average running behaviour of the peers of *i* at time *t*, denoted by a superscript *p* for peers 
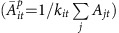
, was regressed on the weather experienced by *i*'s peers at time *t* 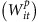 (including temperature and precipitation), peers' time varying and time invariant characteristics 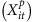 (including age, gender, height, weight, degree, device type and country), an individual fixed effect 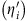, which controls for all observable and unobservable time invariant characteristics of *i*, and time fixed effects to control for temporal variation, such as seasonality or holidays, that may drive individuals' and their peers' running simultaneously 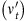. In the second stage, ego's running behaviour at time *t*, *t*+1, *t*+2 and *t*+3 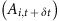 was regressed on peers' running behaviour at time *t* 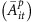 and estimated using the predicted values of 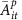 from the first stage, controlling for ego's weather 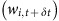 (including temperature and precipitation), ego's time varying characteristics (*X*_*it*_), peers' time varying characteristics 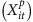, ego-level individual fixed effects (*η*_*i*_) and time fixed effects (*v*_*t*_).

The fitted values estimated in the first-stage regression capture only those changes in peer behaviour caused by changes in weather that ego does not experience. In the second stage, only the variation in peer behaviour precipitated by exogenous weather events is used to estimate peers' social influence on ego's behaviour. In this way, the IV approach enables causal inference by excluding ego's simultaneous effects on peers and variation created by observable and unobservable confounding factors.

**Figure 4:**
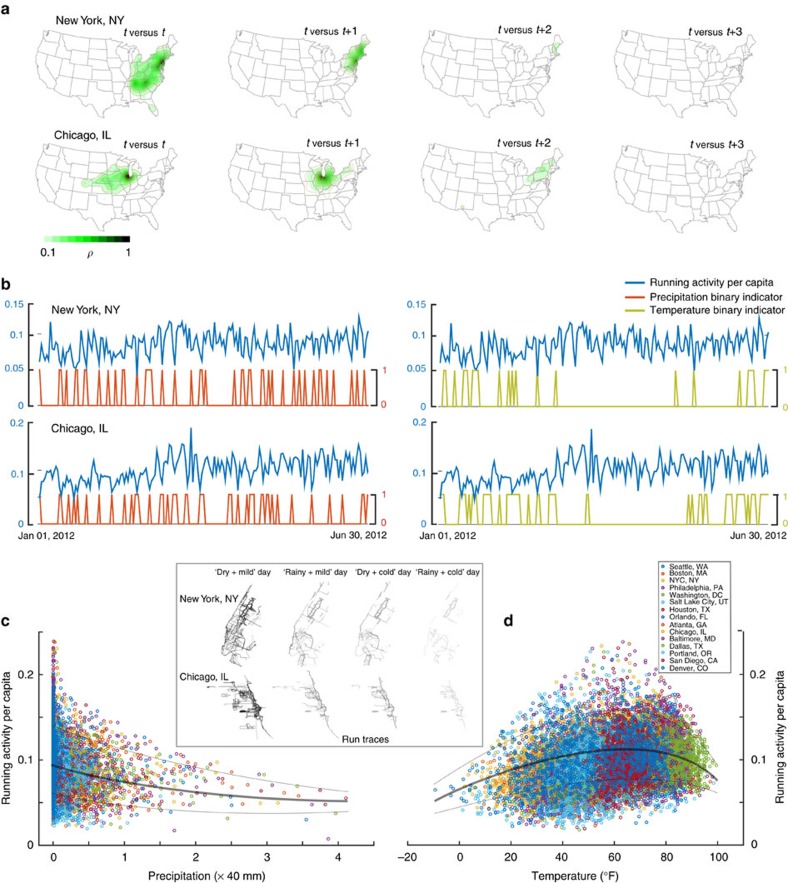
The strength and exogeneity of weather patterns as instrumental variables for running behaviours. Panel (**a**) displays daily correlations between precipitation in New York and Chicago at time *t* and precipitation in the rest of the United States at time *t*, *t*+1, *t*+2 and *t*+3. White colouring indicates no correlation, while progressively darker green colouring indicates proportionally stronger correlations. Panel (**b**) displays daily correlations between running activity per capita and binary indicators of rainfall above the annual average and temperature <35° or >85° in New York and Chicago, respectively. At nearly each spike in rainfall or extreme temperature, running declines markedly, visually demonstrating the strength of the instruments at the daily level. Panel (**c**) displays running activity per capita on the *y* axis and precipitation in millimetres on the *x* axis, while (**d**) displays running activity per capita on the *y* axis and temperature in degrees Fahrenheit on the *x* axis for the top 15 running cities in the United States during the 5-year period. These two panels show strong correlations between the weather and running behaviour and demonstrate that the correspondence of precipitation and temperature to running display different functional forms, necessitating differential approaches to constructing the precipitation and temperature instruments. The inset panel displays the aggregated run traces of the same number of randomly chosen global positioning system-enabled runners on a dry and mild day, a rainy and mild day, a dry and cold day and a rainy and cold day in New York and Chicago, with fewer traces indicating fewer runs by those runners.

To estimate an unbiased causal effect, we must establish that *j*'s weather is highly predictive of *j*'s running behaviour (a strong instrument) and uncorrelated with *i*'s running behaviour (an exogenous instrument)^42^. We constructed an optimal set of variables known as instruments by searching for cases in which *j*'s weather is uncorrelated with *i*'s weather and therefore *i*'s running behaviour. This search is non-trivial because weather patterns are correlated across geography and time. We therefore searched over the daily weather correlation matrices of individual and peer location pairs who run in different cities to find all location pairs that have uncorrelated weather across time. For example, the weather in Chicago today is typically uncorrelated with Boston's weather today but correlated with Boston's weather tomorrow and 2 days from now (see Fig. 4a and Supplementary Fig. 18). So, while the weather in Chicago today is a good instrument for Chicagoans peer effects on runners in Boston today and 3 days from now, it is not a good instrument for Chicagoan's peer effects on runners in Boston tomorrow or 2 days from now. Of the 2.1M located pairs with weather information, we analysed the 600K to 1.2M friend pairs with uncorrelated weather across different regressions, ensuring the validity of our instruments.

Temperature and precipitation also display different non-linear correlations with running. While running is an approximately log linear function of precipitation, it has an inverted U-shaped relationship with temperature (see Fig. 4b,c). We therefore constructed optimal daily individual instruments for the peer effect of *j*'s running behaviour on *i*'s running behaviour using percentile discretized precipitation and temperature in *j*'s city for all location pairs that exhibited uncorrelated weather across time, selecting the optimal instruments using a Post-Lasso penalized first-stage regression that maximizes predictive power and minimizes model complexity^43^. Diagnostics indicate that running is strongly positively correlated with less precipitation and moderate temperatures (see Fig. 4b–d) and that these instruments are both strong and exogenous (see ‘Choosing Optimal Instruments: The Lasso (Post-Lasso) Method' in Supplementary Note 2 for details on the Post-Lasso IV method and its diagnostics, each of which is listed individually for each regression in the table that displays that regression's results). On rainy and cold days, there are marked drops in running. Figure 4b shows daily runner responses to weather changes over 6 months, whereas Fig. 4c,d show responses per capita, thus underlining the fact that we are observing real reactions to weather rather than perhaps the correlations between different types of people who prefer to live in rainy or nice cites and their respective running behaviours.

### Robustness

Numerous diagnostic statistics, manipulation checks and falsification tests validated our results and confirmed their robustness. Wu–Hausman tests confirmed that peer effects in running behaviour are endogenous (we rejected the null hypothesis of exogeneity with *P*<0.00001, *N*=9.5–12M observations, see Supplementary Tables 4–7); F-statistics, which far exceeded the critical threshold of 19.93 for the 10% maximum relative bias due to weak instruments as suggested by Stock and Yogo^44^, confirmed that our instruments are strong (F-statistics ranged from 216 to 430, *N*=9.5M–12M observations, see Supplementary Tables 4–7); and Kleinbergen–Paap rk LM statistics and Hansen–Sargan tests confirmed that our estimates are not under- or over-identified, respectively (KP: *P*<0.00001; Hansen–Sargan tests fail to reject the null hypothesis that our instruments are valid with *P* values ranging from 0.13 to 0.25, *N*=9.5M–12M observations, see Supplementary Tables 4–7). Our analyses were also robust to falsification tests that examined (i) whether friends' future running behaviours influenced ego and (ii) whether unconnected friends influenced each other (both analyses showed no effect); and to multiple econometric specifications and instrument realizations. For example, an alternative specification based on simple binary weather instruments confirmed the validity of our results (see Supplementary Note 4 for more detail on estimation robustness).

But the work is not without its limitations. First, our influence estimates may not generalize to other health behaviours. It could be that diet, alcohol consumption, sexual contact, sleep patterns and other health behaviours are subject to similar social spillovers or that they exhibit different patterns of interdependence. Fortunately, new digital systems are recording and promoting the socialization of these types of behaviours as well. The quantified self-movement is proliferating the number of platforms that record and share health behaviours and we encourage more work using these data to estimate human health interdependence. Second, the individuals in our data may not represent the average person. Our network sample is reasonably representative of the one in five Americans who owns a wearable device and the over 100M people who use fitness trackers worldwide. While this is a substantial and relevant group, they may not represent the average person and peer effects may not operate similarly in the absence of devices that socialize health behaviours. Third, we could not record impression data on when runners observed their peers' running, so we cannot rule out heterogeneity in awareness as a possible explanation for heterogeneity in the treatment effects (that is, that some runners check their friends' activity more often and are therefore more influenced by their friends). Finally, our instruments are only valid for friends who live in different cities and are stronger for compliers than for non-compliers in our sample (compliers are those who do not run in the rain or during extreme temperatures and non-compliers are those who do). We report average peer effects in running behaviour, but since the instruments are valid for friend pairs in different cities and stronger for compliers, we further examine and discuss complier and non-complier behaviour in the ‘Compliers and Non-Compliers' section in Supplementary Note 4 to more precisely characterize our generalizations.

### Data availability

The weather and running data tables and analysis code are all available here (though personal, individual-level data have been redacted for legal and privacy reasons): http://dx.doi.org/10.7910/DVN/VANSK4.

## Additional information

**How to cite this article:** Aral, S. & Nicolaides, C. Exercise contagion in a global social network. *Nat. Commun.* **8,** 14753 doi: 10.1038/ncomms14753 (2017).

**Publisher's note**: Springer Nature remains neutral with regard to jurisdictional claims in published maps and institutional affiliations.

## Supplementary Material

Supplementary Information Supplementary Figures, Supplementary Tables, Supplementary Notes and Supplementary References

Supplementary Movie 1 The GPS recorded running footprint of Manhattan during a sunny Saturday afternoon.

Supplementary Movie 2 The GPS recorded running footprint of Manhattan during a rainy Saturday afternoon.

## References

[b1] Banerjee A., Chandrasekhar A. G., Duflo E. & Jackson M. O. The diffusion of microfinance. Science 341, 1236498 (2013). 23888042 10.1126/science.1236498

[b2] Van den Bulte C. & Lilien G. L. Medical innovation revisited: social contagion versus marketing effort. Am. J. Sociol. 106, 1409–1435 (2001).

[b3] Christakis N. A. & Fowler J. H. The collective dynamics of smoking in a large social network. N. Engl. J. Med. 358, 2249–2258 (2008). 18499567 10.1056/NEJMsa0706154 PMC2822344

[b4] Gomez Rodriguez M., Leskovec J. & Krause A. in *Proceedings of the* *16th ACM SIGKDD*, 1019–1028 (Washington, DC, USA, 2010).

[b5] Lazer D., Rubineau B., Chetkovich C., Katz N. & Neblo M. The coevolution of networks and political attitudes. Polit. Commun. 27, 248–274 (2010).

[b6] Watts D. J. A simple model of global cascades on random networks. Proc. Natl Acad. Sci. 99, 5766–5771 (2002). 16578874 10.1073/pnas.082090499 PMC122850

[b7] Christakis N. A. & Fowler J. H. The spread of obesity in a large social network over 32 years. N. Engl. J. Med. 357, 370–379 (2007). 17652652 10.1056/NEJMsa066082

[b8] Fowler J. H. & Christakis N. A. Dynamic spread of happiness in a large social network: longitudinal analysis over 20 years in the Framingham Heart Study. BMJ 337, 2338 (2008). 10.1136/bmj.a2338 PMC2600606 19056788

[b9] Valente T. W. Network interventions. Science 337, 49–53 (2012). 22767921 10.1126/science.1217330

[b10] McPherson M., Lovin L. S. & Cook J. M. Birds of a feather: homophily in social network. Annu. Rev. Sociol. 27, 415–444 (2001).

[b11] Aral S., Muchnik L. & Sundararajan A. Distinguishing influence-based contagion from homophily-driven diffusion in dynamic networks. Proc. Natl Acad. Sci. 106, 21544–21549 (2009). 20007780 10.1073/pnas.0908800106 PMC2799846

[b12] Aral S. Identifying social influence: a comment on opinion leadership and social contagion in new product diffusion. Market. Sci. 30, 217–223 (2011).

[b13] Manski C. F. Identification of endogenous social influence. Sociol. Methodol. 23, 1 (1993).

[b14] Currarini S., Jackson M. O. & Pin P. Identifying the roles of race-based choice and chance in high school friendship network formation. Proc. Natl Acad. Sci. 107, 4857–4861 (2010). 20212129 10.1073/pnas.0911793107 PMC2841897

[b15] Rosenblat T. & Mobius M. Directed altruism and enforced reciprocity in social networks. Q. J. Econ. 124, 1815–1851 (2009).

[b16] Centola D. The spread of behavior in an online social network experiment. Science 329, 1194–1197 (2010). 20813952 10.1126/science.1185231

[b17] Aral S. & Walker D. Creating social contagion through viral product design: a randomized trial of peer influence in social networks. Manage. Sci. 57, 1623–1639 (2011).

[b18] Aral S. & Walker D. Identify influential and susceptible members of social networks. Science 337, 337–341 (2012). 22722253 10.1126/science.1215842

[b19] Bond R. M. . 61-million-person experiment in social influence and political mobilization. Nature 489, 295–298 (2012). 22972300 10.1038/nature11421 PMC3834737

[b20] Shalizi C. R. & Thomas A. C. Homophily and contagion are generically confounded in observational social network studies. Sociol. Methods Res. 40, 211–239 (2011). 22523436 10.1177/0049124111404820 PMC3328971

[b21] Schwarz N. Self-reports: how the questions shape the answer. Am. Psychol. 54, 93 (1999).

[b22] Falk E., Berkman E. & Leiberman M. From neural responses to population behavior: neural focus group predicts population-level media effects. Psychol. Sci. 25, 439–445 (2012). 10.1177/0956797611434964 PMC3725133 22510393

[b23] Bakshy E., Eckles D., Yan R. & Rosenn I. in *Proceedings of the* *13th ACM Conference on Electronic Commerce*, 146–161 (Valencia, Spain, 2012).

[b24] Bakshy E., Rosenn I., Marlow C. & Adamic L. in *Proceedings of the 21st International Conference on World Wide Web*, 519–528 (Lyon, France, 2012).

[b25] Kramer A. D., Guillory J. E. & Hancock J. T. Experimental evidence of massive-scale emotional contagion through social networks. Proc. Natl Acad. Sci. 111, 8788–8790 (2014). 24889601 10.1073/pnas.1320040111 PMC4066473

[b26] Mason W. & Watts D. Collaborative learning in networks. Proc. Natl Acad. Sci. 109, 764–769 (2012). 22184216 10.1073/pnas.1110069108 PMC3271930

[b27] Festinger L. A theory of social comparison processes. Hum. Relat. 7, 117 (1954).

[b28] Tesser A. Toward a self-evaluation maintenance model for social behavior. Psychology 21, 181–227 (1988).

[b29] Garcia S. M., Tor A. & Gonzalez R. Ranks and rivals: a theory of competition. Pers. Soc. Psychol. Bull. 32, 970–983 (2006). 16738029 10.1177/0146167206287640

[b30] Hankonen N., Absetz P., Ghisletta P. & Renner B. Gender differences in social cognitive determinants of exercise adoption. Psychol. Health 25, 55–69 (2010). 20391207 10.1080/08870440902736972

[b31] Gneezy U., Niederle M. & Rustichini A. Performance in competitive environments: gender differences. Q. J. Econ. 118, 1049–1074 (2003).

[b32] Centola D. & Macy M. Complex contagions and the weakness of long ties. Am. J. Sociol. 113, 702–734 (2007).

[b33] Ugander J., Backstrom L., Marlow C. & Kleinberg J. Structural diversity in social contagion. Proc. Natl Acad. Sci. 109, 5962–5966 (2012). 22474360 10.1073/pnas.1116502109 PMC3341012

[b34] Aral S. & Walker D. Tie strength, embeddedness, and social influence: a large-scale networked experiment. Manage. Sci. 60, 1352–1370 (2014).

[b35] Phan T. & Airoldi E. M. A natural experiment of social network formation and dynamics. Proc. Natl Acad. Sci. 112, 6595–6600 (2015). 25964337 10.1073/pnas.1404770112 PMC4450406

[b36] Miguel E., Satyanath S. & Sergenti E. Economic shocks and civil conflict: an instrumental variables approach. J. Polit. Econ. 112, 725–753 (2004).

[b37] Mehlum H., Miguel E. & Torvik R. Poverty and crime in 19th century Germany. J. Urban Econ. 59, 370–388 (2006).

[b38] Collins W. J. & Margo R. A. *The Labor Market Effects of the 1960 Riots*. Working paper, w10243, National Bureau of Economic Research (2010).

[b39] Angrist J. D. & Krueger A. B. Instrumental variables and the search for identification: from supply and demand to natural experiments. J. Econ. Perspect. 15, 69–85 (2001).

[b40] Angrist J. D. Lifetime earnings and the Vietnam era draft lottery: evidence from Social Security Administrative Records. Am. Econ. Rev. 80, 313–336 (1990).

[b41] Coviello L. . Detecting emotional contagion in massive social networks. PLoS ONE 9, e90315 (2014). 24621792 10.1371/journal.pone.0090315 PMC3951248

[b42] Angrist J. D., Imbens G. W. & Rubin D. B. Identification of causal effects using instrumental variables. J. Am. Stat. Assoc. 91, 444–455 (1996).

[b43] Belloni A., Chen D., Chernozhukov V. & Hansen C. Sparse models and methods for optimal instruments with an application to eminent domain. Econometrica 80, 2369–2429 (2012).

[b44] Stock J. H. & Yogo M. Identification and Inference for Econometric Models: Essays in Honor of Thomas Rothenberg Cambridge Univ. Press (2005).

