# Supplementary material for: Exercise contagion in a global social network
Source: Nat Commun. 2017 Apr 18;8:14753. doi: 10.1038/ncomms14753 (PMC5399289; doi:10.1038/ncomms14753)
Supplement: Supplementary Information — Supplementary Figures, Supplementary Tables, Supplementary Notes and Supplementary References [file ncomms14753-s1.pdf]

Supplementary Note 1:
Data

Running Activity Data

Fitness Tracking. Over the last decade, many companies, including Fitbit, Garmin and Microsoft, among many others, have begun to provide fitness tracking devices which detect and record the distance, duration, pace and calories burned during exercise activity. These devices use GPS and accelerometer technology to record the physical location of and exercise activity engaged in by people who use the platforms. Runners can store, visualize, analyze and share their activity information with friends through social networks operated by the platforms.

We collected and analyzed exercise and social network data from a global fitness tracking network to better understand peer effects in exercise behavior and human health interdependence more broadly. The fitness tracking technology creates an accurate running monitor and provides real-time feedback to runners during and after each run. The technology allows runners to keep track of all of their routes including a breakdown of pace and distance at different points during a run. Runners can analyze their own running data and connect with the website after each run to instantly save the run and share it with friends via the site itself as well as on Twitter, Facebook or other social media.

After each run, the fitness tracker can be connected to the runner's personal account at the platform's website, where personal fitness activity is stored. The website helps runners monitor their running experience with dynamic graphs that compare distance and time between single sessions, as well as weekly and monthly totals.

The website also allows individuals to form social ties and follow other individuals' running activity. Therefore an individual can track her training records but also review her friends' activity as well. The website also allows runners to initiate or participate in competitions with

friends, compare themselves to other runners across the globe and use a mapping tool that illustrates individual running routes, which can be shared with others. Given these features, as we develop in the main text and in argumentation below, our main hypothesis is that peer effects should play a major role in driving individual training and performance patterns.

Data Collection Procedures. The data contain anonymized running activities (distance, duration, pace and calories burned) for each run (and in 29% of runs a GPS trace of the actual run location and trajectory), as well as demographic information for all individuals using the network's fitness tracking devices. Running activity observations were collected over a five year period [We excluded a tiny fraction of data that we believed are not physically possible *daily* running activity performance or are likely error records. We remove runs that exceed a duration of 14 hours (860 minutes) or a distance of 120 km (74.5 miles) or a pace of 1.07 km/min (40 mph).]. At the same time, data on the fitness tracking social network was also collected. The dataset is organized as dyadic relations (from-to) with a timestamp indicating when the social tie was formed. We observe link formation for a period of five years. After an individual forms a social tie, each time they finish a run, their running performance is automatically shared with their friends. At the end of the observation period there are 3.4 million unique social links in the data among 1.1 million people (network nodes) who have at least one connection. This subset of individuals account for 59 million running activity events and 359M kilometers run. Supplementary Table 1 displays summary statistics for the sample demographics and running activity respectively. The giant connected component contains $\sim$1 million nodes (Supplementary Figure 1), the degree distribution is roughly power law and the average person has four social connections (mean degree $\simeq$ 4).

Demographics of the Networked Sample. Since our work is focused on identifying social influence and behavioral contagion in running and exercise behaviors, we focus our analysis on socially connected runners. There are approximately $\sim$1.1 million individuals that account for

59M million running activity events and 359 million total kilometers run in the social network during the five years of observation. The average daily number of running activities per person in the sample is 0.12 runs (S.D.=0.02). The average distance per run is 6.61 km (S.D.=4.60), the average run duration is 44.50 minutes (S.D.= 32.17), while the average number of calories burned during a run is 489 cal (S.D.=373).

We categorize individuals' age, gender and country of application usage at the time of their registration. We also categorize individuals according to their Body Mass Index (BMI) at the time of registration, a metric that is defined as the body mass divided by the square of the body height (kg/m^2) and is frequently used to assess how much an individual's body weight departs from what is normal or desirable for a person's height. The networked data do not track or measure BMI, so using World Health Organization (WHO) recommendations, we delineate five categories of BMI range: $<$18.5 (underweight), 18.5-25 (healthy weight), 25-30 (overweight), 30-35 (moderately obese), $>$35 (severely obese) ["BMI Classification". Global Database on Body Mass Index. World Health Organization. 2006.]. Supplementary Figure 2A displays demographic distributions of our sample with respect to gender, age, country and BMI. There are almost twice as many men as women in the network. 20 to 40 year olds account for more than 80% of the networked runners. The United States has the largest share of the network, with the rest of the countries sharing the remaining 20% of runners, proportional to their population. Finally, 50% of runners are at a normal weight, 35% of runners are moderately overweight and only 2% of the sample is underweight.

Supplementary Figure 2B and C display daily activity measured in number of runs per day as well as the average pace per run taken by individuals in different demographic categories respectively. Women are more active than men on average and run at a faster pace. Interestingly, older people (especially in their 50's and 60's) run more frequently than younger people. However, these are the ages that experience the slowest pace during runs. Runners from Japan

are very frequent runners, however their pace of running is significantly lower compared to individuals from other countries. Finally, individuals who are at a normal weight or are slightly overweight are more active runners both in terms of number of daily runs but also in terms of the pace of their runs.

When we analyze exercise at the daily level, we see that activity depends not only on the day of the week but also on the specific time of day (see Supplementary Figure 4). Running is more popular during the weekend and less popular at the beginning of the work week. Also, people in our sample prefer an afternoon run to running early in the morning (Supplementary Figure 4). All of the above mentioned time fixed effects (day specific, season specific and year specific) are controlled for in our subsequent analysis for identifying exercise influence.

Social Network Data

The underlying running social network is organized in dyadic form (from-to) with a timestamp indicating when the social tie was formed. After an individual forms a social tie, each time they finish a run, their running performance is shared automatically with their running friends. At the end of the observation period we have $\sim$1.1M individuals that are connected by $\sim$3.4M links.

The running social network is a sparse network with average degree (the number of connections or ties an individual has) close to 3.7 (S.D.=8.2). As is fairly typical, the network has a heavy-tailed degree distribution. While the vast majority of runners have a small number of connections, there is a small number of people with many connections, with the maximum of 1330 (Supplementary Figure 5). Long tail degree distributions are a common characteristic of natural and socio-technical networks, from protein-protein interactions to human mobility systems (*2*).

Weather Data

Since our objective is to use the weather as an instrument to identify and quantify exercise influence, we are interested in collecting complete daily weather data (precipitation and temperature) for the period of observation. We collect weather data at the station (or tower) level worldwide through the National Oceanic and Atmospheric Administration (NOAA) (*3*) [NOAA National Climatic Data Center. http://www.ncdc.noaa.gov, http://doi.org/10.7289/V5D21VHZ, access date: Sep 8, 2015.] and other international weather agencies [European Climate Assessment & Dataset project (http://eca.knmi.nl), UK Climate – Met Office (http://www.metoffice.gov.uk).]. Each weather station (or tower), g, is associated with its exact latitude and longitude to five decimal places. Supplementary Figure 6 displays the location of the 47,559 available weather stations in 196 countries worldwide, revealing our nearly complete global coverage. In Supplementary Figure 7 we plot the location of the approximately 33K weather stations in the contiguous United States. In order to measure the correlation between population density and weather stations' spatial distribution, we collect population data from the 2010 US Census in the $\sim$ 3,000 counties in the contiguous United States [http://www.census.gov/data.html] and compare the spatial distribution of population with the spatial distribution of weather towers at the county level (Supplementary Figure 8). The correlation coefficient between the two distributions is 0.59 suggesting that in highly populated areas the distribution of weather stations is very dense compared with sparsely populated areas. All of these observations are helpful for our analysis below, since one of our objectives is to pair weather to individual runners with high precision.

We assign each individual j to a weather station g by choosing the station that is closest to his/her running activity if GPS locations are recorded for that individual or otherwise to the address they provided during registration (Supplementary Figure 9). Individuals who are training in areas more than 30 km away from the closest weather station are excluded from the

analysis (about $\sim$3% of our sample) since it is impossible to identify correctly the weather they experience. At the end of this process we have a 1:1 matching between stations and fitness tracking individuals and therefore time series of precipitation and temperature for the period of interest for each individual.

Precipitation. For each day during the period of social network observation, we collected daily precipitation data at the weather station level. Daily precipitation is recorded in tenths of a millimeter and indicates the total precipitation measured by each of the world weather stations each day. The precipitation is always a positive number and values greater than zero indicate a rainy day. The maximum precipitation recorded in our data is 179mm (7 inches)(see Supplementary Table 2).

Temperature. For each day in the same period of observation, we collected daily temperature data at the weather station level. The daily temperature is recorded in tenths of a Celsius degree and indicates the maximum temperature that the weather station experiences each day. The temperature can be either positive or negative in either the Celsius or Fahrenheit scale. In our dataset the minimum temperature recorded was -43°C (-45 ^{o}F) and the maximum was 54.5^{o}C (129 ^{o}F) (see Supplementary Table 2).

Supplementary Note 2:
Model Specification and Estimation Procedures

A Causal Model of Exercise Contagion

Since dyadic models can be biased due to heterogeneity in individuals' connectivity, from here on we specify estimation models at the ego level.

Let A_{it} be the fitness activity of individual i on day t. Individual fitness activity is measured in daily distance (km), duration (min), pace (km/min) or calories burned (cal). We define c_{ijt} to be the binary indicator of the existence (=1) or not (=0) of a relationship between individuals i and j at time t (Adjacency Matrix). We can then define the degree (connectivity) of an individual i at time t as $k_{it} = \sum_j c_{ijt}$.

We specify four factors that affect fitness activity. First, there are time fixed effects – which include holidays, weekends, marathon days etc – that we denote with ν_t for each time period t. Second, there are time-invariant, individual fixed effects that separate individuals with different fitness habits and motivations, that we denote with η_i for each individual i. Third, there are time varying characteristics like degree. Finally, there is exogenous variation in environmental conditions that perturb individual utility for outdoor training, like changes in weather patterns. These effects, which we denote as w_{it} for individual i during time period t, are time-varying and individual-specific (through the location of individual).

We further specify an endogenous factor that influences the fitness habits of an individual that is a function of their social ties. In other words we assume that each individual i's fitness activity on day t or the next couple of days, $t + \delta t$, is influenced by the fitness activity of her social circle, i.e. from the specific activity on day t of each individual j to whom she is connected.

Using the above definitions and assumptions we specify a linear model for the running

activity of individual i at time $t + \delta t$ (δt=0,1,2,.. days) as:

$$A_{i,t+\delta t} = \beta \bar{A}^p_{it} + \gamma X^p_{it} + \theta X_{it} + \alpha w_{i,t+\delta t} + \mu_1 A_{i,t-1} + .. + \mu_n A_{i,t+\delta t-1} + \eta_i + \nu_{t+\delta t} + \varepsilon_{it}, \quad (1)$$

In the special case where δt =0, we assume a memoryless model where individuals influence each other only within a day and not across time periods.

The above model assumes that the running activity $A_{i,t+\delta t}$ of Ego i at time $t+\delta t$ (δt =0,1,2,..) is an additive linear function of other factors measured at the same time $t + \delta t$ or previous time periods $t + \delta t - 1, ..., t$, including the time fixed effects $\nu_{t+\delta t}$, the effect of exogenous factors $w_{i,t+\delta t}$ (temperature and precipitation i experiences on the day of consideration $t + \delta t$); the effect β of an endogenous factor $\bar{A}^p_{it} = 1/k_{it} \sum_j c_{ijt} A_{jt}$ (the average running activity of the social contacts of i on day t), the effect of the running history of the individual on previous days $A_{i,t+\delta t-1}, ..., A_{i,t-1}$, and time invariant factors captured by the individual fixed effects η_i. The inclusion of the individual-fixed effect η_i controls for all time invariant characteristics of the Ego i, which further reduces the likelihood that correlation in fitness habits is driven by choice of social connections. We also control for time varying characteristics of i, like degree, through X_{it} and also for time varying and time invariant characteristics of peers like degree, age, gender, height, weight and country (X^p_{it}).

The usual assumption is that the error term, ε_{it}, is i.i.d. (independent and identically distributed), but this is clearly violated here since our estimation takes place in a population of individuals connected in a network. A natural approach in such cases is to assume "clustered errors" i.e. that observations within a network cluster u are correlated in some unknown way, inducing correlation in ε_{it}, within u. In the presence of clustered errors, OLS or IV estimates are unbiased but standard errors may be wrong, leading to incorrect inference in a surprisingly high proportion of finite samples.

Although this model seems straightforward to estimate, the reciprocal influence of an in-

dividual on her friends' running state and vice versa makes it difficult to interpret a simple association in their fitness behavior. Correlation in exercise habits may not only result from pairwise mutual influence, but also from triangles in the social network. For example, j might influence l's fitness behavior, which in turn affects i's fitness behavior, and so on. We address the inherent endogeneity of contagion in the next section using instrumental variable theory, a well known and understood method widely used in the econometrics literature for identifying causal effects in non-networked data.

Instrumental Variable Theory

Endogeneity exists when an explanatory variable is related to the error term in the population model of the data generating process – for example, due to omitted variables, measurement error, or other sources of simultaneity bias or reverse causality, which causes the ordinary least squares estimator (OLS) to be biased and inconsistent (*4*). Instrumental Variables (IV) is a method of estimation that is widely used in many economic, educational and epidemiology related applications, that provides a way to obtain consistent parameter estimates (*5, 6*). Examples where instrumental variables theory is applied to causal inference include estimating the effect of class size on test scores, the effect of smoking on health, or the effect of financial aid on college enrollment – all problems where the explanatory variable correlates with the error term (*7*).

For example, Angrist uses random variation in the likelihood of military service created by the draft lottery to identify the causal effect of military service on wages (*5*). Since individuals with lower expected wages are more likely to choose to serve in the military, estimating the raw correlation between military service and wages produces a biased estimate of the causal effect. However, since the draft lottery is randomized and therefore uncorrelated with wages, an individuals draft lottery number can be used to identify the causal effect of military service on

future earnings. Military service is first regressed on randomly assigned draft lottery numbers. Then, future wages are regressed on the predicted values of military service from this first stage regression. The draft lottery affects the likelihood of military service because ones lottery number determines whether one is drafted. But, the lottery is uncorrelated with past wages and future wage potential (except through their impact on the likelihood of military service) because lottery numbers are randomly assigned. Since the military service driven by the draft lottery is unrelated to the future wage potential of those who serve, unbiased estimates of the average causal effect of military service on wages can be established by examining the effect of military service mandated by the draft lottery on the future wages of those who were randomly selected to serve.

Assume we specify a simple linear model $Y = \beta X + .. + u$, where the explanatory variable X is correlated with the error term u. An *instrument* Z is a variable that (a) is correlated with the endogenous explanatory variable Y (conditional on the other covariates), and (b) does not correlate with the error term u. The first assumption requires that there is an association between the instrument Z and the variable being instrumented X while the second assumption excludes the instrument Z as a regressor in the model of Y. In linear models, (a) and (b) are basic requirements for using IV theory.

If the instrument Z is valid, i.e. satisfies the above conditions of relevance and exogeneity, then the coefficient β can be estimated using an IV estimator in a Two Stage Least Squares (2SLS) specification. In the first stage, each explanatory variable that is an endogenous covariate in the equation of interest X is regressed on all of the exogenous variables in the model, including both exogenous covariates in the equation of interest and the excluded instrument, Z. In the second stage, the regression of interest is estimated as usual, except that in this stage each endogenous covariate is replaced with the predicted values from the first stage $\hat{X}$ as $Y = \hat{\beta}\hat{X} + .. + u$. $\hat{\beta}$ is the IV estimation of the causal effect β. Let's assume that Ego's friends

running activity, $\bar{A}^p_{it} = 1/k_{it}\sum_j c_{ijt}A_{jt}$, is an endogenous factor in our estimation model of Supplementary Equation 1. Let's further suppose that an exonenous variable, w_{jt} is available for each of Ego's friends $j = 1, 2, ...k_{i,t}$, from which we can design an instrument, $W^p_{it} = f(w_{jt})$ that perturbs the endogenous factor, $\bar{A}^p_{it}$, and meets the relevance and exogeneity conditions of a valid instrument. Then, a Two Stage Least Squares (2SLS) estimation model can be specified as follows:

$$\textbf{1}^{st}\textbf{ stage}: \bar{A}^p_{it} = \lambda W^p_{it} + \alpha' w_{i,t+\delta t} + \gamma' X^p_{it} + \theta' X_{it} + \mu'_1 A_{i,t-1} + .. + \mu'_n A_{i,t+\delta t-1} + \eta'_i + \nu'_{t+\delta t} + \varepsilon'_{it}$$

$$\textbf{2}^{nd}\textbf{ stage}: A_{i,t+\delta t} = \beta \bar{A}^p_{it} + \gamma X^p_{it} + \theta X_{it} + \alpha w_{i,t+\delta t} + \mu_1 A_{i,t-1} + .. + \mu_n A_{i,t+\delta t-1} + \eta_i + \nu_{t+\delta t} + \varepsilon_{it}, \quad (2)$$

where in the second stage $\bar{A}^p_{it}$ are the *predicted values* from the first stage that are assumed to be orthogonal to ε_{it} and β is the social influence coefficient (causal effect) that we are interested in estimating.

The credibility of these estimates hinges on the selection of suitable instruments. Good instruments are often created by policy changes. For example, the cancellation of a federal student-aid scholarship program may reveal the effects of aid on some students' outcomes. Other natural and quasi-natural experiments of various types are commonly exploited, for example, Miguel, Satyanath, and Sergenti (2004) use weather shocks to identify the effect of changes in economic growth (i.e., declines) on civil conflict (*8*). In the context of peer effects and social influence IV theory was used by Tucker (2008) to identify peer effects in TV streaming technology adoption using the World Cup as an instrument (*9*).

Recently Coviello et *al.* (2014) used rainfall as an instrument to measure contagion in emotional expressions on Facebook (*10*). They use data on emotional expressions on Facebook as a proxy for happiness and other emotions aggregated to the city level across the US and develop a network of friendship across the largest US cities. They use rain as instrument for the peer cities' emotions to detect causal peer effects in emotional contagion across cities. The main

differences between our paper and Coviello et *al.* (2014) paper are: (i) we use a behavior that we measure directly using tracking devices rather than inferring behavior or emotion through written expressions (the later is subject to similar measurement error as self reports). (ii) We estimate an individual level model, rather than a city aggregated model. This provides incredible precision and fidelity because individuals may have friends in cities that are not typical of residents of that city. This heterogeneity in connectivity is lost in city level aggregations of networked data. (ii) We use a robust methodology of developing, designing and choosing optimal percentile discretized rainfall and temperature instruments that generate the greatest variation in peers' running behavior while still satisfying the exclusion restriction. Coviello et *al.* (2014) use a simple binary indicator of rain. This allows us to finely tune the instruments and to measure individual level compliance (whether individuals run through the rain and extreme temperatures), allowing us to mathematically characterize the generalizability of our estimates (see the Section on Compliers and Non-Compliers below).

The Weather as an Instrument

We propose the weather as an instrument for detecting contagion in exercise habits across social network ties. Instead of changing individuals' running activity directly with an experimental treatment (which seems difficult and expensive), we let the weather do the work for us by measuring how weather-induced changes in individual exercise behavior predict changes in the individuals' friends' exercise behavior. First, we must establish the two requirements for using an instrumental variable approach, relevance, i.e. detecting a strong relationship between the instrument (weather) and the endogenous predictor (friends' running activity), and *exogeneity*, i.e. weather changes that friends experience do not directly affect Ego's running behavior.

Relevance. Weather is unlikely to be affected by individuals running activity, therefore if we find a relationship between the two, it suggests that weather influences the running activity

of individuals and not vice versa. We have two distinct weather indicators available, rainfall and temperature. In Supplementary Figure 10, we plot the daily, per capita running activity for fifteen large cities in the United States as a function of the precipitation and temperature experienced in those cities. It is clear that more precipitation is associated with lower running activity, in a monotonic fashion (similar to the graph in Supplementary Figure 11A). On the other hand, the relationship between running activity and temperature is non-monotonic, suggesting that very high and very low temperatures are associated with reduced exercise activity (similar to the graph in Supplementary Figure 11B). We further access the granularity of our dataset to visualize how bad weather affects running. In Supplementary Movies 1 and 2 we visualize the footprint of running in Manhattan (New York, NY) during a sunny Saturday afternoon and during a rainy Saturday afternoon, showing how bad weather inhibits running (see Supplementary Figures 25 and 26). We have established a strong association between weather and running activity suggesting that precipitation and temperature can potentially serve as instrumental variables in order to detect peer effects in fitness habits and activities. We test the strength of these instruments more formally in our evaluation of the first stage regressions as described below.

Exclusion Restriction–Exogeneity. One of the biggest concerns in a model like this is that friends' weather is correlated, so the instrument might actually just be a proxy for the direct effect of weather on a person's running behavior – a violation of the "exclusion restriction" (*5*). Unfortunately, weather patterns are highly correlated, both spatially and temporally. For example, geographically proximate regions are more likely to experience the same weather on the same day. A rainy day in Chicago, IL implies with large probability that it is a rainy day in Indianapolis, IN (200 miles south of Chicago). At the same time there is a high probability of rain tomorrow in Boston, MA if it is raining today in New York, NY (180 miles south-west of Boston). In Supplementary Figure 12 we plot a typical precipitation pattern over the Midwest and south of the United States and how this pattern moves, over geography and time, over the

next two consecutive days. In Supplementary Figure 13 we visualize how the weather in four large cities in the U.S. correlates with weather in the rest of the contiguous U.S. on the same day (left panel), one day later (middle-left panel), two days later (middle-right panel) and three days later (right panel). Our data reveal some reliable weather patterns over the U.S. that typically move from from west to east. In addition, the data reveal that weather pattern correlations across time drop near zero after three or more days.

The only symmetry we should expect between the rows of subplots appear in Supplementary Figure 13 is for the same day weather correlations at the level of the cities of interest. For example if there is no same day correlation between New York NY and Chicago IL (as shown in the top left subplot) we should not expect same day correlation between Chicago and New York NY (in the second row left subplot). Otherwise the same day weather correlation figures (left panels) should not be symmetric since each city's co-variation with other cities may not be the same. For example, while in the upper left figure there is correlation between the same day weather of New York and Columbus OH, there is no correlation between Chicago IL and Columbus OH in the second-row left figure (see Supplementary Table 3).

In order to meet the exclusion restriction requirements in our IV analysis, we only consider how running behavior is transmitted between social dyads that have no correlation in weather patterns. To do so we compute the sample Pearson correlation coefficient between the weather of all links ($i_{t+\delta t}$,j_t) using the weather history for 45 months as (*11*):

$$\rho_{i_{t+\delta t},j_t} = \frac{\sum_t (w_{i,t+\delta t} - \overline{w}_i)(w_{jt} - \overline{w}_j)}{\sqrt{\sum_t (w_i - \overline{w}_{i,t+\delta t})^2}\sqrt{\sum_t (w_{jt} - \overline{w}_j)^2}}, \quad (3)$$

where $w_{i,t+\delta t}$ denotes the weather that individual i (Ego) experiences on day $t + \delta t$ (δt=0,1,2,.. days), $w_{j,t}$ is the weather that individual j (Friend) experiences on day t and $\overline{w}_i$ and $\overline{w}_j$ are the average weather i and j respectively experience for the period of observation. The summation in Supplementary Equation 3 runs from day $t = 1$ and for a period of $\sim$5 years. The correlation

coefficient ranges from -1 to 1. A value of 1 implies that individual i on day $t + \delta t$ experiences the same exact weather as individual j on day t. In the case of $\delta t = 0$, a correlation coefficient equal to 1 means that the two individuals are experiencing exactly the same weather, i.e. they are living in the same city. On the other hand, a value of -1 implies that whenever i experiences good weather on day $t + \delta t$, j experiences bad weather on day t. Finally, a correlation value of 0 implies that there is no linear correlation between the weather the two individuals experience. Using the correlation values of all social dyads in our network $\rho_{i_{t+\delta t}, j_t}$ we are able to drop those who experience similar weather (i.e. large correlations) by imposing a correlation threshold of $\rho_c = +0.025$ over which any dyad is excluded from our analysis. From the total number of $\sim$2.1M links in our analysis (for which we can geographically locate and find weather information for both nodes connected by the link), we exclude $\sim$1.51M when we consider same day correlations (t vs t), $\sim$1.44M when we consider one day difference correlations ($t + 1$ vs t), and $\sim$0.9M when we consider two day difference correlations ($t + 2$ vs t). In the case of more than two day differences, the correlation in almost all dyads drops below the threshold point and therefore almost no link is excluded (see also right panel in Supplementary Figure 13). We provide detailed tests of the sensitivity of our analysis to this choice of threshold below. The results of these analyses show that our estimates are not sensitive to the choice of threshold.

Choosing Optimal Instruments: The Lasso (Post-Lasso) Method

For each peer j on day t we consider a collection of binary weather indicators: N for the rain that the individual experiences $r_{jt}^{(n)}$ ($n = 0, 1, ...N - 1$), and M for her temperature $\theta_{jt}^{(m)}$ ($m = 1, ...M - 1$). In order to generate binary indicators we divide the range of precipitation and temperature that individuals experience into percentiles and define areas where the precipitation and temperature are larger or smaller than point percentiles, as we described in Supplementary Figure 14. In this way, we take into account and differentiate peers who live in cities with

different average weather patterns. For example, a day with 2 inches of precipitation in Seattle is different than a day with the same amount of precipitation in Los Angeles. We assume that a rainy day in a typically dry city has larger marginal effects on running behavior than in a city that is typically wet. Let's assume that we have two peers, one living in a typically wet and cold city and one in a typically dry and warm city. We design our binaries to make sure that 2 inches of rain and a temperature of 20°C in both cities activates different precipitation and temperature binaries. In this way we take into account city specific weather effects.

After we develop the different weather binaries for each peer (Supplementary Figure 14), we use a LASSO (Least Absolute Shrinkage and Selection Operator) penalized regression in the first stage of the 2SLS model in Supplementary Equation 2, to choose the set of instruments that best predicts the endogenous variable and then use them in the two stage least squares model (*12–15*). The equation we use to estimate the LASSO penalized regression can be written as:

$$\bar{A}^p_{it} = \lambda_{r,0}W^{(r,0)}_{it} + .. + \lambda_{r,N-1}W^{(r,N-1)}_{it} + ... + \lambda_{\theta,0}W^{(\theta,0)}_{it} + .. + \lambda_{\theta,M-1}W^{(\theta,M-1)}_{it} + \\ +\alpha' w_{i,t+\delta t} + \gamma' X^p_{it} + \theta' X_{it} + [\mu'_1 A_{i,t-1} + .. + \mu'_n A_{i,t+\delta t-1}] + \eta'_i + \nu'_{t+\delta t} + \varepsilon'_{it}, \quad (4)$$

where $\bar{A}^p_{it}$ is the average running activity of i' s peers, $W^{(r,n)}_{it}$ $(n = 0, 1, ..N-1)$ is the sum of *rain* binary indicators $r^{(n)}_{j,t}$ over i' s peers, $W^{(r,n)}_{it} = \sum_j r^{(n)}_{j,t}$ and $W^{(\theta,m)}_{it}$ $(m = 0, 1, ..M-1)$ is the sum of the *temperature* binary indicators $\theta^{(m)}_{j,t}$ over i' s peers, $W^{(\theta,m)}_{it} = \sum_j \theta^{(m)}_{j,t}$.

Using the LASSO, we select optimal instruments that minimize the sum $\sum(\bar{A}^p_{it} - \hat{\bar{A}}^p_{it})^2$ subject to $|\lambda_{r,0}| + |\lambda_{r,1}| + ... + |\lambda_{\theta,M-1}| \leq s$, where the first sum is taken over observations in the dataset and $\hat{\bar{A}}^p_{it}$ are the predicted values of the regression. The bound s is a tuning parameter that controls the tradeoff between the penalty and the fit (loss/likelihood). When s is large enough, the constraint has no effect and the solution is just the usual multiple linear least squares regression of $\bar{A}^p_{it}$ on $W^{(r,0)}_{it}$, $W^{(r,0)}_{it}$,...,$W^{(\theta,M-1)}_{it}$. However for smaller values of s

($s \geq 0$), the solutions are shrunken versions of the least squares estimates. Often, some of the coefficients λ's are zero. Choosing s is like choosing the number of predictors to use in the first stage regression model, and cross-validation is a good tool for estimating the best value for s. After using the above procedure for picking the "best" set of instruments for the endogenous variable $\bar{A}_{it}^{p}$, we return to the 2SLS model (Supplementary Equation 2) and run the two stages (including an OLS first stage regression) using the chosen set of instruments W_{it}^{p}.

Estimating Treatment Effect Heterogeneity

In order to gain insight regarding influential members of the running community we study, we introduce heterogeneous treatment effects in the Ego level estimation by examining time invariant features of runners. First, we are interested in whether a more active friend is more influential than a less active one. To measure these effects we split the neighborhood of each Ego j into subsets of peers according to the ratio between their overall running activity over the period of observation and Ego's total running activity. We first calculate the overall running activity of the Ego and each peer ($A_i = \sum_t A_{i,t}$ and $A_j = \sum_t A_{j,t}$, $j = 1, 2, ...k_{it}$ respectively) and calculate all ratios of peer's running activity to Ego's running activity, $\Lambda_{ji} = A_j/A_i$, $j = 1, 2, ...k_{i,t}$, where k_{it} is the number of peers Ego i has at time t. We then define several continuous ranges for Λ_{ij}: (i) $\Lambda < 1/16$, (ii) $1/16 \leq \Lambda < 1/8$, (iii) $1/8 \leq \Lambda < 1/4$, (iv) $1/4 \leq \Lambda < 1/2$, (v)$1/2 \leq \Lambda < 2$, (vi) $2 \leq \Lambda < 4$, (vii) $4 \leq \Lambda < 8$, (iix) $8 \leq \Lambda < 16$ and (ix) $\Lambda \geq 16$, and categorize peers into subsets according to the value of Λ_{ij}. We then define an interaction model using the Ego level model (Supplementary Equation 1) as a baseline, with δt =0 as follows:

$$A_{it} = \sum_{h=1}^{9} \beta_h \bar{A}_{it}^{p(h)} + \gamma X_{it}^{p} + \theta X_{it} + \alpha w_{i,t+\delta t} + \mu_1 A_{i,t-1} + \eta_i + \nu_t + \varepsilon_{it}, \tag{5}$$

where $\bar{A}_{it}^{p(h)}$ is the average running activity of the peers at time t in the subgroup h ($h = i, ii, ...ix$). We estimate the average running activity of the Ego's peers in each range of nine

subcategories of Λ ($h = i, ii, ...ix$) defined above at time t. We then estimate the instrumental variable regression, using the precipitation and temperature binary indicators chosen from the LASSO penalized regression to instrument for the endogenous term.

Since the above model only gives us partial information, in a relative sense, on how individuals with different levels of activity influence each other, for completeness we examine how two very active friends (or mostly inactive friends) influence each other. To do that we first separate all individuals into two categories, active ("H") and inactive ("L") by comparing their total running activity over the period of observation to the average running activity of all runners. For each Ego i, we then split her/his neighborhood (peers $j = 1..k_{it}$) into active $pa =$"H" and inactive $pa =$"L" runner groups and define a model of exercise contagion that includes an interaction term for the level of activeness as:

$$A_{it} = \sum_{ea=H,L} \sum_{pa=H,L} \beta_{ea,pa} E^{(ea)} \bar{A}_{it}^{p(pa)} + \gamma X_{it}^{p} + \theta X_{it} + \alpha w_{i,t+\delta t} + \mu_1 A_{i,t-1} + \eta_i + \nu_t + \varepsilon_{it}, \quad (6)$$

where $\bar{A}_{it}^{p(pa)}$ is the average running activity of the peers at time t in the active group ($pa = H$) and the inactive group ($pa = L$) and $E^{(ea)}$ is an indicator variable denoting whether ego is active ($ea = H$) or inactive ($ea = L$). The four possible scenarios are: (i) Ego is an active runner and peers are active runners, (ii) Ego is an active runner and peers are inactive runners, (i) Ego is an inactive runner and peers are active runners, (iv) Ego is an inactive runner and peers are inactive runners. We then estimate an instrumental variable regression to identify the causal effect β, using the precipitation and temperature binary indicators chosen from the LASSO penalized regression to instrument for the endogenous interaction term.

We next consider an interaction model that investigates the role of running consistency (in time) in social contagion. We are interested in whether a consistent running friend is more influential than a sporadically active friend, or the other way around. First, for each runner in our dataset, we identify the periods during which their running activity is consistent. We

do so by isolating the periods where activity is continuous without any inactivity lasting more than 2 weeks (see Supplementary Figure 15 below for illustration). By following the same methodology for all the available runners, we identify 376,000 distinct running activities with an average consistency length of 34 days (S.D. 62 days).

We then define an individual as a consistent runner if the largest consistency period lasts more than 1 month. Otherwise we define the individual as an inconsistent runner. For each ego j we split her/his neighborhood (peers $j = 1..k_{it}$) into consistent "C" and inconsistent runner groups "I" and we define a model of exercise contagion that includes an interaction term for running consistency as follows:

$$A_{it} = \sum_{ec=C,I} \sum_{pc=C,I} \beta_{ec,pc} E^{(ec)} \bar{A}_{it}^{p(pc)} + \gamma X_{it}^{p} + \theta X_{it} + \alpha w_{i,t+\delta t} + \mu_1 A_{i,t-1} + \eta_i + \nu_t + \varepsilon_{it}, \quad (7)$$

where $\bar{A}_{it}^{p(pc)}$ is the average running activity of the peers at time t in the consistent group ($pc = C$) and the inconsistent group ($pc = I$) and $E^{(ec)}$ is an indicator variable denoting whether ego is consistent ($ec = C$) or inconsistent ($ec = I$). The interaction term in the above equation considers all four possible scenarios: (i) Ego is a consistent runner and peers are consistent runners, (ii) Ego is a consistent runner and peers are sporadic runners, (i) Ego is a sporadic runner and peers are consistent runners, and (iv) Ego is a sporadic runner and peers are sporadic runners. We then estimate an instrumental variables regression to identify the causal effect β, using precipitation and temperature instruments chosen by the LASSO penalized regression to instrument for the endogenous interaction term..

Furthermore, we are interested in how gender affects exercise influence. For each Ego i we split their neighborhood (peers $j = 1..k_{it}$) into male "M" and female "F" peers and we define a model of exercise contagion that includes an interaction term for gender as follows:

$$A_{it} = \sum_{eg=M,F} \sum_{pg=M,F} \beta_{eg,pg} E^{(eg)} \bar{A}_{it}^{p(pg)} + \gamma X_{it}^{p} + \theta X_{it} + \alpha w_{i,t+\delta t} + \mu_1 A_{i,t-1} + \eta_i + \nu_t + \varepsilon_{it}, \quad (8)$$

where $\bar{A}_{it}^{p(pg)}$ is the average running activity of the peers at time t in the male group ($pg = M$) and the female group ($pg = F$) and $E^{(eg)}$ is an indicator variable denoting whether ego is male ($eg = M$) or female ($eg = F$). The interaction term in the above equation considers all four possible scenarios: (i) male peers/male Ego, (ii) female peers/male Ego, (iii) male peers/female Ego and (iv) female peers/female Ego. We then estimate an instrumental variables regression to identify the causal effect β, using precipitation and temperature instruments chosen by the LASSO penalized regression to instrument for the endogenous interaction term.

Finally, using a slightly modified model, we investigate the role of same-gender and cross-gender influence. For each Ego i we split their neighborhood (peers $j = 1..k_{it}$) into a *same-gender* group "S" and a *cross-gender* group "C" of peers with respect to Ego' s gender. We define a model of exercise contagion that includes an interaction term for gender as follows:

$$A_{it} = \sum_{g=S,C} \beta_g \bar{A}_{it}^{p(g)} + \gamma X_{it}^{p} + \theta X_{it} + \alpha w_{i,t+\delta t} + \mu_1 A_{i,t-1} + \eta_i + \nu_t + \varepsilon_{it}, \tag{9}$$

where $\bar{A}_{it}^{p(pg)}$ is the average running activity of the peers estimated in the same-gender group ($g =$"S") and in the cross-gender group ($g =$"C") at time t. The two categories are (i) same gender and (ii) cross gender. We then estimate an instrumental variables regression to identify the causal effect β, using precipitation and temperature instruments chosen by the LASSO penalized regression to instrument for the endogenous term.

Testing Structural Theories of Social Contagion

The Complex Contagion theory contends that multiple sources of exposure to a behavior increase the likelihood that an individual adopts the behavior (*16, 17*). We test whether complex contagion explains contagion in our exercise data by investigating the impact of the number of running friends on Ego's running behavior. We do so by defining a model for Ego's activity (dependent variable) where the endogenous effect is the number of friends that are active on

the same day, $\#\text{FR}_t$, controlling for the total number of connections Ego has, k_{it}, and all other characteristics of Ego and their peers, elements of X_{it} and X_{it}^p respectively as follows:

$$A_{it} = \beta(\#\text{FR}_t) + \gamma X_{it}^p + \theta X_{it} + \alpha w_{i,t+\delta t} + \mu A_{i,t-1} + \eta_i + \nu_t + \varepsilon_{it}. \tag{10}$$

We use an instrumental variable method where we instrument the endogenous variable $\#\text{FR}_t$ with the precipitation and temperature binary indicators chosen from the LASSO penalized regression. We anticipate that the number of active friends is a positive predictor of social influence.

To double check the functional relationship between exercise influence and the number of active friends, we define an additional model where we have two endogenous regressors, the number of active friends $\#\text{FR}_t$ and its square $(\#\text{FR}_t)^2$:

$$A_{it} = \beta_1(\#\text{FR}_t) + \beta_2(\#\text{FR}_t)^2 + \gamma X_{it}^p + \theta X_{it} + \alpha w_{i,t+\delta t} + \mu A_{i,t-1} + \eta_i + \nu_t + \varepsilon_{it}. \tag{11}$$

The functional form of exercise influence and the number of active friends depends on the absolute value and sign of the estimation coefficient β_2. We use the 2SLS methodology by instrumenting the two endogenous regressors β_1 and β_2 with at least two weather binary indicators that we choose using a LASSO regression analysis.

We then examine how the structural diversity of the Ego's neighborhood affects exercise influence by investigating how fitness contagion is driven by the number of (running) active connected components in Ego's network. To count the number of connected components that are active, we first go through all nodes (runners), identify their immediate connections (neighborhood), and find the connected components in the neighborhoods (see Supplementary Figure 16 for illustration).

We then define a model of Ego's activity in which the endogenous effect is the number of connected components that are active on the same day ($\#\text{CR}_t$) controlling for the total number

of connections that Ego has, k_{it}, an element of X_{it}, as follows:

$$A_{it} = \beta(\#\text{CR}_t) + \gamma X_{it}^p + \theta X_{it} + \alpha w_{i,t+\delta t} + \mu A_{i,t-1} + \eta_i + \nu_t + \varepsilon_{it}. \quad (12)$$

We use a 2SLS instrumental variable method to estimate the causal effect β using a subset of the binary weather indicators to instrument for the number of running (active) components $\#\text{CR}_t$.

Ugander et al. recently showed in a Facebook study that the probability of contagion is highly correlated with the number of connected components in an individual's contact neighborhood, rather than with the actual size of the neighborhood (*18*). We test this hypothesis by defining an exercise contagion model where we have two endogenous regressors, the number of active friends $\#\text{FR}_t$ and the number of active connected components in Ego's neighborhood $\#\text{CR}_t$, making sure that we control for Ego's connectivity at time t, k_{it} an element of X_{it}, as follows:

$$A_{it} = \beta^f(\#\text{FR}_t) + \beta^c(\#\text{CR}_t) + \gamma X_{it}^p + \theta X_{it} + \alpha w_{i,t+\delta t} + \mu A_{i,t-1} + \eta_i + \nu_t + \varepsilon_{jt}. \quad (13)$$

We use an instrumental variable method to estimate the two causal effects β^f and β^c by instrumenting the two endogenous variables with a subset of weather binary indicators chosen using a LASSO penalized regression.

One of the most widely studied social factors theorized to affect the strength of social influence is structural embeddedness, the extent to which individuals share common peers. In this subsection we investigate how structural embeddeness moderates social influence in exercise habits, while simultaneously controlling for confounding factors that can bias inference in networked settings. Here, we adopt the conventional network structural measure of embeddedness, defined as the number of common friends shared by individuals and their peers (*19–21*). We first split the neighborhood of each Ego i into two groups of peers, one in which peers share no common friends with Ego $e_{ij} = 0$ and one in which all peers share at least one common friend

with Ego $e_{ij} = 1$, where e is a categorical variable. We then propose an interaction model of exercise contagion based on our estimation model as follows:

$$A_{it} = \beta \bar{A}_{it}^{p(E)} + \gamma X_{it}^{p} + \theta X_{it} + \alpha w_{i,t+\delta t} + \mu_1 A_{i,t-1} + \eta_i + \nu_t + \varepsilon_{it}, \tag{14}$$

where $\bar{A}_{it}^{p(E)}$ is the average running activity of the set of peers that are embedded. We also estimate a model that examines the influence (β) of set of peers in Ego's neighborhood that are not embedded ($\bar{A}_{it}^{p(NE)}$). We use an instrumental variable method to identify the effect β by instrumenting the endogenous effect $\bar{A}_{it}^{p(E)}$ ($\bar{A}_{it}^{p(NE)}$) with a subset of the available weather binary indicators.

Supplementary Note 3:
Results

Model-Free Evidence of Exercise Clustering

We first present some model free evidence for running activity clustering in the network. In Supplementary Figure 17 we plot individuals' daily activity measured by the number of runs they engage in [runs] as a function of the number of connections individuals have, displayed as PlotBoxes with delineations for minimum, first quartile, median, third quartile, and maximum. It is clear that running activity is an increasing function of the number of friends individuals have. However, this is only evidence of correlations in the system. We have to set up a framework to identify the causal effect of social influence that can separate it from other explanations of correlation like homophily or other confounds. Our framework uses weather patterns as an instrument for peers running activity to understand causality in exercise influence.

Peer Effects–IV Estimation Results

While the fixed effect models provide evidence of the possible existence of peer effects in the system, their estimates are biased. To produce unbiased estimates of the magnitude of peer effects in exercise, we execute the IV estimation method described in Supplementary Note 2.

We organize our data into Ego i and day t panels, where for each day of observation we have the daily running activity of Ego A_{it} as measured by the four available indicators of running performance: distance [km], pace [km/min], duration [min] and calories burned [cal] during a day t.

We next set up the different control variables for Ego's own running activity. First, for each Ego i on each day t, we have two meteorological binary indicators, one for the precipitation and one for the temperature $w_{it} = (r_{it}, \theta_{it})$. The binary indicator for Ego's precipitation on

day t (r_{it}) takes value 1 if the total precipitation that Ego experiences on day t is larger than their seasonal average calculated for a period of 2 months, centered on day t, and 0 otherwise. The binary indicator for Ego's temperature on day t takes the value 1 if the temperature that Ego experiences on day t is either in the range $(-\inf, \langle\theta_i\rangle - 3/4(\langle\theta_i\rangle - \theta_{i,min})]$ or in the range $[\langle\theta_i\rangle + 3/4(\theta_{i,min} - \langle\theta_i\rangle), \inf)$, where $\theta_{i,min}$, $\theta_{i,max}$ and $\langle\theta_i\rangle$ are the minimum, maximum and average temperature that Ego i experiences respectively in a 2 month period centered on day t. We also control for Ego's past running activity $A_{i,t-1}$, $A_{i,t-2}$.

To specify the endogenous effect $\bar{A}^p_{it}$, for each Ego i we identify their running buddies and compute the Pearson correlation coefficient between the weather each peer experiences and the weather Ego experiences, dropping all the peers whose correlation coefficient is larger than the threshold described in the "Weather as an Instrument" in Supplementary Note 2 . By excluding all links for which peers' weather correlates with Ego's weather, we ensure the validity of our exclusion restriction. Using the remaining peers (k_{it} in total), we calculate their average running activity as $\bar{A}^p_{it} = 1/k_{it} \sum_j c_{ijt} A_{jt}$, where A_{jt} is the running activity of the peer j and c_{ijt} is the adjacency matrix. Note that when we are interested in identifying same day social influence ($\delta t = 0$), we take into account time zone in order to design $\bar{A}^p_{it}$ making sure that peers running took place *before* Ego's running.

We also prepare the time varying characteristics of Ego i (X_{it}) as well as the time varying and time invariant characteristics of peers that we control for in our model (X^p_{it}). The former includes the degree of i while the latter includes the average degree of peers, the average age of peers, the average height and weight of peers, the fraction of peers that are men (women) and the fraction of peers that are located in US, UK, Canada, or another country.

We finally specify the weather variables that instrument for the endogenous effect $\bar{A}^p_{it}$. For the k_{it} peers of each Ego i, we identify the C_j unique weather towers to which peers are most closely located. Note that the number of unique weather towers that the k_{it} peers are closely

located to is always $C_j \leq k_{it}$, since it is possible that more than one of Ego's peers are located in the same city. By considering only the towers (or cities) with distinct weather, we make sure that we do not violate the exclusion criterion of the IV model. Each of these C_j weather towers experiences different weather. For each of these weather towers, we define N rain ($r_{l,t}^{(0)}, ..r_{l,t}^{(N-1)}$, $l = 1, 2, ..C_j$) and M temperature ($\theta_{l,t}^{(0)}, ..\theta_{l,t}^{(M-1)}$, $l = 1, 2, ..C_j$) binary indicators according to the methodology described in Supplementary Figure 14. We finally define N+M variables as the sum of the weather binary indicators over the C_j unique weather towers as $R_t^{(0)} = \sum_{l=1}^{c_j} r_{l,t}^{(0)}$, .. ,$R_t^{(N-1)} = \sum_{l=1}^{c_j} r_{l,t}^{(N-1)}$,$\Theta_t^{(0)} = \sum_{l=1}^{c_j} \theta_{l,t}^{(0)}$, .., $\Theta_t^{(M-1)} = \sum_{l=1}^{c_j} \theta_{l,t}^{(M-1)}$. The weather variables $R_t^{(n)}$, $n = 0, 1, .., N-1$ and $\Theta_t^{(m)}$, $m = 0, 1, .., M-1$ are the candidates to serve as instruments for the endogenous effect $\bar{A}_{it}^{p}$.

To choose the optimal set of weather instruments (W_{it}^{p}), we run a LASSO penalized regression in the first stage of our 2SLS model. We regress our endogenous variable $\bar{A}_{it}^{p}$ on all the N+M weather variables, controlling for all other exogenous variables that appear in the second stage, including time and Ego fixed effects. The LASSO produces a sparse model, where only the coefficient of $R_t^{(7)}$ and $\Theta_t^{(2)}$ are nonzero when we consider *distance* as the running activity indicator. In the case of *duration*, *pace* and *calories*, the LASSO chooses $R_t^{(7)}$ and $\Theta_t^{(3)}$ as the best combination of instruments. This Post LASSO method selects the optimal set of instruments that maximizes the strength of the first stage regression while minimizing complexity. But, it is important to note that because theoretical guarantees in the IV method apply to OLS estimates in the first stage, the Post LASSO methods optimally selects variables for the first stage regression, which we ultimately estimate using OLS (see (*15*)). We estimate the 2SLS model specified in Supplementary Equation 2 using the above selected weather variables as instruments to estimate the causal effect β.

However, as mentioned above, there is interdependence between observations in the same network cluster u. Therefore our IV estimation will give us unbiased coefficient estimates, but

most likely give us wrong estimates of the standard errors. We therefore correct our standard errors by first partitioning our graph into 15144 communities of average size 7.7 nodes (S.D. = 41), using the classic method of optimal modularity, proposed by Newman et *al*. (*22*) and cluster our standard errors with respect to these discovered network communities (we discuss this method in more detail in "Non-Independence: Clustering and Standard Errors" in Supplementary Note 4).

Supplementary Tables 4 to 7 report the estimates–with standard errors, t-statistics, p-values and 95% confidence intervals–for the first and second stage of the 2SLS regression for the model in Supplementary Equation 2. Each Table refers to a different exercise indicator (4: distance, 5: pace, 6: duration and 7: calories). In each Table, the estimates for time-lagged causal peer effects with δt =0,1,2 are also displayed. The standard errors are cluster corrected. These estimates are graphically displayed in Figure 1 of the main manuscript (influence coefficient estimates with 95% confidence intervals).

The contagion parameter estimates β from the second stage regression are always positive and significant, indicating significant social influence in running behaviors. On average, an extra kilometer run by Ego's peers causes an increase in Ego's running activity by more than a quarter of a kilometer on the same day. To illustrate the main result, lets assume that a runner (A) usually runs 6km at a pace of 7 minutes per kilometer (0.143 km/minute) and their friend (B) usually runs 6km at a pace of 8 minutes per kilometer (0.125 km/minute). An extra kilometer run by B (an increase from 6km to 7km) causes A to increase their running distance by 0.3km (from 6km to 6.3km). Also, a 0.01 km per minute increase in runner Bs pace (from 0.125 km/minute to 0.135 km/minute) causes runner A to increase their pace by 0.003 km per minute (from 0.143 km/minute to 0.146 km/minute).

Diagnostics. We compute multiple diagnostic statistics to assess the quality and robustness of our estimates. First, we verify that the model is not underidentied. For that we use

Kleinbergen-Paap rk LM statistic to test the null hypothesis of underidentication (*23*). All of our tests reject the null hypothesis (Supplementary Tables 4 to 7). Second, we verify that the instruments are good predictors of the endogenous explanatory variable in the first-stage regression. "Weak" instruments would cause poor predicted values in the first-stage regression and poor estimation in the second-stage regression. To ensure the instruments are not weak, the Cragg-Donald Wald F statistic should exceed the critical threshold suggested by Stock and Yogo (*24*). In all cases the Cragg-Donald Wald F statistic exceeds the critical thresholds, suggesting that the weather variables we select are strong instruments. Furthermore, we need to test if the friends' activity $\bar{A}_{it}^{p}$ is indeed endogenous. The Wu-Hausman F statistics tests the null hypothesis that the variable under consideration is exogenous (*25, 26*). All of our tests are significant and reject the null hypothesis and confirm that friend's activity is indeed endogenous (see diagnostic statistics in Supplementary Tables 4 to 7). Finally, we test for overidentification using the Hansen-Sargan (HS) test (*27*). The null hypothesis is that the instruments are valid (i.e., uncorrelated with the error term) and that the excluded instruments are correctly excluded from the estimated equation. Results of the Hansen and Sargan (HS) tests suggest we are using valid instruments.

Comparison of IV Estimates with an OLS Model

In order to compare the Instrumental Variable estimates with a less sophisticated model, we build the corresponding OLS model. This model takes into account a) the social links considered for the IV estimation, i.e. all the links where Ego's weather is not correlated with Friend's weather, b) it is considered on the Ego level and c) it is based on a daily level panel exactly as our main specification model. The OLS model is a simple regression model similar to the one in Supplementary Equation 2 that control for ego's X_{it} and peer's X_{it}^{p} time varying and time invariant characteristics (including age, gender, height, weight, degree, device type and coun-

try), without considering the individual and time fixed effects. In Supplementary Table 8 we present the results of the OLS model peer effects estimation along with a comparison to the IV estimations. The OLS models without ego-level fixed effects overestimate influence by between 71%-82%, depending on the dependent variable in question; These are useful comparisons that shed light on the value of the IV estimator.

Treatment Effect Heterogeneity

In this subsection we present the results from our heterogeneous treatment effects models 5 to 9, defined in the "Estimating Treatment Effect Heterogeneity" in Supplementary Note 2. In all of the models we first split each Ego i's network neighborhood into several groups according to each model's specifications and specify the endogenous term as the interaction between the group type and the average running activity of the peers in the group. We instrument for the endogenous term with an interaction between the group type and the weather variables in order to identify the causal peer effect β.

Supplementary Table 9 reports the estimates – with standard errors, t-statistics, p-values, 95% confidence intervals, and diagnostic statistics – for the second stage of the 2SLS regression for the interaction model in Supplementary Equation 5. Surprisingly, friends who are less active than Ego influence Ego's running habits more. Specifically, peers with four to eight times less running activity compared to Ego's running activity are the most influential on average with an influence coefficient close to 0.5. On the other hand, an extra kilometer run by a more active friend has no significant effects on Ego's running activity. Results of the model are graphically displayed in Figure 2A of the main manuscript.

Supplementary Table 10 reports the estimates – with standard errors, t-statistics, p-values, 95% confidence intervals, and diagnostic statistics – for the second stage of the 2SLS regression for the interaction model in Supplementary Equation 6. It is clear that active individuals

are generally more susceptible to exercise influence, especially when influence is coming from inactive runners. It is also worth mentioning that in conjunction with the results in Supplementary Table 9, active friends have no significant influence on non-active runners. Results of the model are graphically displayed in Figure 2B of the main manuscript.

Supplementary Table 11 reports the estimates – with standard errors, t-statistics, p-values, 95% confidence intervals, and diagnostic statistics – for the second stage of the 2SLS regression for the interaction model in Supplementary Equation 7. Similar to the results on active and inactive runners, here we find that inconsistent peers are very influential over consistent runners and that consistent peers do not influence inconsistent runners. We also find that the influence coefficient is almost identical when Ego and peers are both either consistent or inconsistent. Results of the model are graphically displayed in Figure 2C of the main manuscript.

In Supplementary Table 12 we report the estimates – with standard errors, t-statistics, p-values, 95% confidence intervals, and diagnostic statistics – for the second stage of the 2SLS regression for the gender interaction model in Supplementary Equation 8. Men tend to be more influential runners, especially with respect to their influence on other men. However, the influence coefficient estimates become insignificant when we consider men influencing women. On the other hand, women exert significant influence on other women and on men. Results of the model are graphically displayed in Figure 2D of the main manuscript. Finally, in Supplementary Table 13 we report the estimates along with errors and diagnostic statistics – for the second stage of the 2SLS regression for the same-gender and cross-gender interaction model in Supplementary Equation 9. We find that same gender influence is significantly larger (t-stat=4.98) than cross-gender influence. Results of this model are graphically displayed in the inset of Figure 2D of the main manuscript.

Structural Theories of Social Contagion

Complex Contagion. The Complex Contagion Theory of behavioral contagion suggests that the number of behaviorally active friends in one's Ego network is a significant (non-linear) predictor of social influence. In Supplementary Table 14 we summarize the first and second stage coefficients as well as the accompanying identification diagnostic statistics for the 2SLS regression of the model specified in Supplementary Equation 10. It is clear that the number of Ego's friends that are active is a strong predictor of exercise influence. The results are graphically displayed in Figure 3A of the main manuscript (diamonds) when we use running distance as the performance indicator and in Supplementary Figure 20 (diamonds) when we use duration as the running performance indicator.

In Supplementary Table 16 we summarize the first and second stage coefficients as well as the accompanying diagnostic statistics for the 2SLS regression of the model in Supplementary Equation 12. The number of Ego's neighborhood connected components that are running (active) is a strong predictor of exercise influence as well. The results are graphically displayed in Figure 3A of the main manuscript (squares) when we use distance as the running performance indicator and in Supplementary Figure 19 (squares) when we use duration as the running performance indicator.

In Supplementary Table 15 we report the second stage results for the social influence coefficients β_1 and β_2. The negative though small β_2 estimate suggests that there are diminishing returns to additional peers' influence.

Structural Diversity. The Structural Diversity Theory of behavioral contagion suggests that the number of behaviorally active components in one's Ego network, rather than the number of active friends is the main predictor of social influence. In Supplementary Table 17 we report the results of the first and second stage of the 2SLS methodology (estimates, standard errors and diagnostic statistics) for the causal effect of the number of active friends and the number of

connected components on Ego's running behavior when we include both of them in the same estimation model (Supplementary Equation 13). Consistent with results found by Ugander et al (*18*), we find that the number of running active connected components is a strong positive predictor of exercise influence while the number of running friends becomes a negative predictor when controlling for structural diversity. The two values are statistically different (t-stat=15.9). This is strong evidence for the Structural Diversity theory of exercise contagion. The results are graphically displayed in Figure 3B of the main manuscript for running distance and in Supplementary Figure 20 for running duration.

Embeddedness. The Embeddedness Theory of behavioral contagion suggests that the more mutual friends two people share, the more influential they will be on one another. Supplementary Table 18 reports the estimates – with standard errors, t-statistics, p-values, 95% confidence intervals, and diagnostic statistics – for the second stage of the 2SLS regression for the interaction model in Supplementary Equation 14. The results, illustrating the correspondence between structural emboddedness and influence, are displayed in Figure 3D of the main manuscript for social influence on run distance and in Supplementary Figure 21 for influence on run duration. We observe that individuals are statistically significantly more influential on peers with whom they are embedded, i.e. share common friends (t-stat=2.45). This result is evidence for the Embeddedness Theory and is consistent with the empirical evidence described in Aral and Walker (2014) (*21*)

Supplementary Note 4: Robustness

First Stage Regressions

The first stage regression along with its diagnostic statistics is an important component of the two stage least square estimator for instrumental variables theory, since it reveals the strength and suitability of the instruments. It is important to verify that the instruments are good predictors of the endogenous explanatory variable in the first-stage regression. “Weak” instruments would cause poor predicted values in the first-stage regression and therefore poor estimation in the second-stage regression. It is therefore important to confirm that the F-statistics of the first stage are strong. To ensure the instruments are not weak, the Cragg-Donald Wald F statistic should exceed the critical threshold suggested by Stock and Yogo (19). With their tabulated values Stock and Yogo first fix the largest relative bias of the two stage least squares estimator (2SLS) relative to OLS that is acceptable. In this sense the test answers the question: can we reject the null hypothesis that the maximum relative bias due to weak instruments is 10% (or 5%, etc). In all of the cases of the 2SLS IV regressions we are considering the Cragg-Donald Wald F exceeds the critical thresholds suggesting that the weather variables we select are strong instruments. For example, in the diagnostic statistics for our main results in Supplementary Tables 4 to 7 all of the Cragg-Donald Wald F statistics are in the range between 200 and 500 well above the $\sim$20 Stock-Yogo weak identification test critical value for the 10% maximum relative bias due to weak instruments.

Exogeneity

We test for any direct causal relationship between weather changes that Ego’s peers experience and Ego’s running activity. As we discussed in the sections on model specification, an important

component of the model is to exclude all links between individuals whose weather patterns are correlated. In order to investigate if our methodology is reliable, we consider here a simple model with Ego's running activity as the dependent variable and as independent variables the instruments we used for our model estimation Z_{jt}, controlling for all other exogenous factors and for peer's running activity, as follows:

$$A_{it} = \delta W_{it}^p + \gamma X_{it}^p + \bar{A}_{it}^p + \theta X_{it} + \alpha w_{i,t+\delta t} + \mu_1 A_{i,t-1} + \eta_j + \nu_t + \varepsilon_{it}. \quad (15)$$

In Supplementary Table 19 we report the estimates of δ for the four running indicators (distance, pace, duration and calories). The non significance of the estimates for δ indicate that peers' weather does not correlate with Ego's running (except through its effect on peer running), providing evidence of the exogeneity of the instruments.

Non-Independence: Clustering and Standard Errors

The usual assumption in these types of models is that ε_{it} is iid (independent and identically distributed). But this assumption could be violated in many of the cases we consider. A natural generalization while working on networks is to assume "clustered errors" – that observations within group u are correlated in some unknown way, inducing correlation in ε_{it} within u, but that groups u and v do not have correlated errors. In the presence of clustered errors, OLS and IV estimates are both still unbiased but standard errors may be quite wrong, leading to incorrect inference in a surprisingly high proportion of finite samples.

The optimal way of avoiding such a problem in a network topology would be having a large number of connected components whose errors are uncorrelated with each other ["Components" refers to completely unconnected clusters, while "clusters" (in the Supplementary Information and in the literature) can be defined with varying levels of connectedness between and within groups of nodes that make up a cluster. Conceptually, components are a subset of clusters. Ugander et al use "components" when defining structural diversity, and so we maintain

the same terminology and definition when testing the structural diversity theory, meaning we create "components" by taking an ego network, eliminating all ties to ego and then defining the remaining clusters in that ego network that have no connection to other outside nodes in the ego network as "components." We use "clusters" here to deal with the non-independence of our data: we use a clustering algorithm to find clusters of highly connected nodes (but they are not completely unconnected from other nodes, only connected to other nodes below a threshold of connectivity) and treat individuals within those clusters as non-independent for the purposes of estimating the standard errors in our models.]. However, another common property of socio-technical systems is that a giant connected component typically accounts for a large fraction of the network. This is something we observe in our network as well: $\sim$90% of runners are concentrated in a giant connected component.

For that reason, we first partition our graph into 15144 communities of average size 7.7 nodes (S.D. = 41), using the classic method of optimal modularity proposed by Newman et *al*. (*22*). A good metric for how independent the clusters in a graph partition are is the fraction of friends that are within community. In our case, the average over all individuals' fraction of friends that are within community is close to 82%. In other words, on average 8 out of 10 friends are within cluster while 2 of 10 are across clusters. We can achieve this high level of partition because the running network is quite sparse, which demonstrates another important quality of this dataset for obtaining reliable estimates of social contagion. All standard errors presented in Supplementary Tables 4 to 27 reflect clustering with respect to the network communities. While in most cases clustering of the standard errors increases the 95% confidence intervals, all of our results remain highly significant.

Alternative Instrument Design

As an alternative robustness check, we design the instruments in a slightly different, less sophisticated way to make sure our more complex specification is not somehow producing spurious results. Instead of specifying N (and M) binary indicators for the precipitation (and temperature) that peers experience and using the Post LASSO method to identify which set of binaries to use as instruments, here we propose a global design of instruments that are identical across peers. For each peer we define two simple binary indicators for rain and temperature respectively. For each individual j on each day t we consider a binary rain indicator r_{jt} that is equal to 1 if the precipitation that individual j experiences on day t, pr_{jt}, is more than a seasonal average $\overline{pr}_{jt}$, and 0 otherwise (Supplementary Figure 22A). We compute the seasonal average as the average precipitation in a two month period, from 30 days before to 30 days after the current day t, $\overline{pr}_{jt} = 1/60 \sum_{\tau=t-30}^{t+31} pr_{j\tau}$. In this way, we account for seasonality as we differentiate 2-inches of precipitation during a wet winter from 2-inches of precipitation during a dry summer. At the same time, for each individual, we build a binary indicator for temperature $\theta_{j,t}$ that is equal to 1 if the temperature $T_{j,t}$ that individual j experiences is outside a normal temperature range (T_0, T_1)=(35,85)^{o}F and 0 otherwise (Supplementary Figure 22B).

After we establish the exclusion criterion by dropping dyads whose weather is correlated using the methodology described in "Weather as an Instrument" in Supplementary Note 2, we define the two variables that will serve as instruments for the average activity of Ego's friends in our analysis as the sum of the binaries over the set of unique weather towers ($l = 1..c_j$) that peers of Ego i are located close to, $R_{ft} = \sum_{l=1}^{c_j} r_{l,t}$ for the rain and $\Theta_{ft} = \sum_{l=1}^{c_j} \theta_{l,t}$ for temperature respectively. Note that these two instruments by design are expected to negatively correlate with the running activity of peers. We then use Supplementary Equation 2) to estimate the causal effect β.

Supplementary Tables 20 to 23 report the estimates–with standard errors, t-statistics, p-

values, 95% confidence intervals and regression diagnostics–for the first and second stage of the 2SLS regression for the model in Supplementary Equation 2 using R_{ft} and Θ_{ft} as instruments. Each Table refers to a different exercise indicator (20: distance, 21: pace, 22: duration and 23: calories) and the estimates for time-lapsed causal peer effects are indexed by δt =0,1,2. The standard errors shown are also corrected with respect to clustering. The estimates are graphically displayed in Supplementary Figure 23 (point influence estimates with 95% confidence intervals) along with the results from the LASSO-based instrument design that we presented in "First Stage Regressions" in Supplementary Note 4. It is clear from this analysis that our results are robust to using the above method of designing the instruments and are not significantly different from the coefficients we estimate using the more sophisticated LASSO method, suggesting that our results of exercise influence are robust with respect to instrument modifications.

Falsification Tests

Falsification Test 1. If our procedure is correctly estimating exercise influence, we would not expect to be able to predict individuals' running activity using their friends' future weather and running. Here, we test a falsification model using the same instrumental variables technique described above to estimate the effect of friends' future running activity on Ego's running activity today. We arbitrarily choose $t + 60$ days ($\sim$2 months later) as a point in time far enough in the future where we suspect that friends' running should not affect Ego's running today. We then modify the Ego level model with δt =0 (Supplementary Equation 1) to shift the independent variable 60 days ($\sim$2 months) into the future:

$$A_{it} = \beta \bar{A}^p_{i,t+60} + \gamma X^p_{it} + \theta X_{it} + \alpha w_{i,t+\delta t} + \mu A_{i,t-1} + \eta_i + \nu_t + \varepsilon_{it}. \quad (16)$$

We then use the instrumental variable method using the weather indicators 60 days afterwards as an instrument for friends' activity to predict the exercise influence coefficient β.

Supplementary Table 24 reports the estimates – with standard errors, t-statistics, p-values, 95% confidence intervals, and diagnostic statistics – for the first and second stage of the 2SLS regression for our first falsification test. Note that we use exactly the same network that we use to identify our main results in Supplementary Tables 4 to 7. The estimates from the second stage regression, β, are not statistically significant and they are much lower in magnitude than those estimated for the model in Supplementary Equation 1, suggesting the robustness of our main estimates.

Falsification Test 2. If our results are correct, we also expect that exercise influence should not exist ($\beta \sim 0$) if we use the wrong social network for identification. Here, we test a falsification model where we randomly manipulate the underlying running-buddy network and try to identify the exercise influence coefficient using the same identification strategy. We randomly rewire each in the underlying social network with probability 1, making sure that the total number of links remains unchanged. We then use the model in Supplementary Equation 1 to identify the exercise influence coefficient.

In Supplementary Tables 25, 26 and 27 we report the estimates along with the diagnostic statistics for the first and second stage of the 2SLS regression for *three realizations* of the second falsification test. Results are also graphically shown in Supplementary Figure 24. It is clear that by breaking the structure of the real running buddy network we get completely different results in the second stage and the social influence coefficient is no longer positive and significant. We check the results for multiple realizations and find that in all cases the social influence coefficient is near zero and insignificant. These results again suggest that our results are quite robust.

Sensitivity Analysis on the Weather Correlation Threshold

As discussed above in the sections on model specification, one procedure we followed was to exclude all links between individuals whose weather patterns correlate. For our main analysis, we set a weather correlation threshold of $\rho_c = +0.025$ over which we drop all links (i.e. exclude links in which Ego and Friend experience weather correlation coefficients larger than 0.025). A question that naturally arises in this context is: how robust are our estimates to variations in this threshold? In this subsection we examine the sensitivity of our estimates on the weather correlation threshold choice by reporting estimates for a range of correlation threshold values between $\rho_c = [0.01, 0.1]$. In Supplementary Table 28 we report the estimates from the second stage regression of in Supplementary Equation 2. We find that within this range of correlation thresholds near zero (0.01 to 0.10) our estimates are relatively insensitive to the choice of threshold, suggesting that our choice of $\rho_c = 0.025$ provides robust estimates.

Compliers and Non-Compliers

We finally analyze the running population to understand who "complies" with shocks from our instruments and who does not in order to make our generalizations more precise. For each runner, we calculate the faction of runs that happen on a rainy day f_i (mean=0.1899, S.D.=0.1294). We then define a linear model which uses all the available time-invariant characteristics of individuals (average daily activity, age, gender, height, weight, country and others) to predict compliance with the weather instrument (not running when it rains) while we control for how much rain an individual experiences:

$$f_i = \alpha X_i + nr_i + \varepsilon_i, \tag{17}$$

where nr_i is the total number of raining days i experiences. In Supplementary Table 29 we show the results of the above regression. The results show that the more active someone is

the more likely they are to run through the rain. Also men, younger individuals and those of normal weight are more likely to run in the rain, while height plays no role. Finally we find that individuals in the United States, UK, Canada, Germany, Spain, Brazil, France and the Netherlands are more likely to run on a rainy day than people in Australia, Mexico and Japan. These results help us more precisely characterize the types of people to whom our results most directly generalize.

Supplementary References

1. Kobourov S G (2012) Spring Embedders and Force Directed Graph Drawing Algorithms *arXiv preprint arXiv:1201.3011*.

2. Albert R, Barabási A-L (2002) Emergence of Scaling in Random Networks *Reviews of modern physics*, 74:47.

3. Menne M J et al. (2012) An Overview of the Global Historical Climatology Network-Daily Database. *Journal of Atmospheric and Oceanic Technology* 29:897-910.

4. Bullock J G, Green D P, Ha S E (2010)Yes, but what' s the mechanism (don' t expect an easy answer). *Journal of personality and social psychology* 98:550.

5. Angrist J D, Imbens G W, Rubin D B (1996) Identification of causal effects using instrumental variables. *Journal of the American statistical Association* 91:444.

6. Eckles D, Kizilcec R F, Bakshy E (2016) *MIT Working Paper*.

7. Stock J W, Watson M W (2003), *Introduction to econometrics*, vol. 104 (Addison Wesley Boston,).

8. Miguel E, Satyanath S, Sergenti E (2004) Economic Shocks and Civil Conflict: An Instrumental Variables Approac. *Journal of Political Economy* 112:725.

9. Tucker C (2008) Identifying formal and informal influence in technology adoption with network externalities. *Management Science* 54:2024.

10. Coviello L et al. (2014) Detecting emotional contagion in massive social networks, *PloS one* 9:e90315.

11. Pearson K (1901) LIII. On lines and planes of closest fit to systems of points in space. *The London, Edinburgh, and Dublin Philosophical Magazine and Journal of Science* 2:559.

12. Tibshirani R (1996) Regression shrinkage and selection via the lasso. *Journal of the Royal Statistical Society. Series B (Methodological)* 1:267–288.

13. Friedman J, Hastie T, Tibshirani R (2001) The elements of statistical learning, vol. 1 (Springer series in statistics Springer, Berlin).

14. Athey S, Imbens G (2015) Machine Learning Methods for Estimating Heterogeneous Causal Effects. *arXiv preprint arXiv:1504.01132*.

15. Belloni A, Chen D, Chernozhukov V, Hansen C (2012) Sparse models and methods for optimal instruments with an application to eminent domain. *Econometrica* 80:2369.

16. Centola D, Macy M (2007) Complex contagions and the weakness of long ties. *American Journal of Sociology* 113:702.

17. Centola D (2010) The spread of behavior in an online social network experiment. *Science* 329:1194.

18. Ugander J, Backstroke L, Marlow C, Kleinberg J (2012) Structural diversity in social contagion. *Proc. Natl. Acad. Sci. USA* 109:5962.

19. Valente T W (1996) Social network thresholds in the diffusion of innovations. *Social networks* **18** 69.

20. Uzzi B (1997) Social structure and competition in interfirm networks: The paradox of embeddedness. *Administrative science quarterly* 42:35–67.

21. Aral S, Walker D (2014) Tie Strength, Embeddedness, and Social Influence: A Large-Scale Networked Experiment., *Management Science* 60:1352.

22. Newman M E (2006) Modularity and community structure in networks. *Proc. Natl. Acad. Sci. USA* 103:8577.

23. Kleibergen F, Paap R (2006) Generalized reduced rank tests using the singular value decomposition, *Journal of econometrics* 133:97.

24. Stock J H, Yogo M (2005) Identification and inference for econometric models: Essays in honor of Thomas Rothenberg (Cambridge Univ. Press, Cambridge, UK).

25. Wu D M (1973) Alternative tests of independence between stochastic regressors and disturbances. *Econometrica: journal of the Econometric Society* 41:733–750.

26. Hausman J A (1978) Specification tests in econometrics. *Econometrica: Journal of the Econometric Society* 46:1251–1271.

27. Sargan D, Desai M (1988) Lectures on advanced econometric theory (Blackwell, Oxford UK).

Supplementary Figures

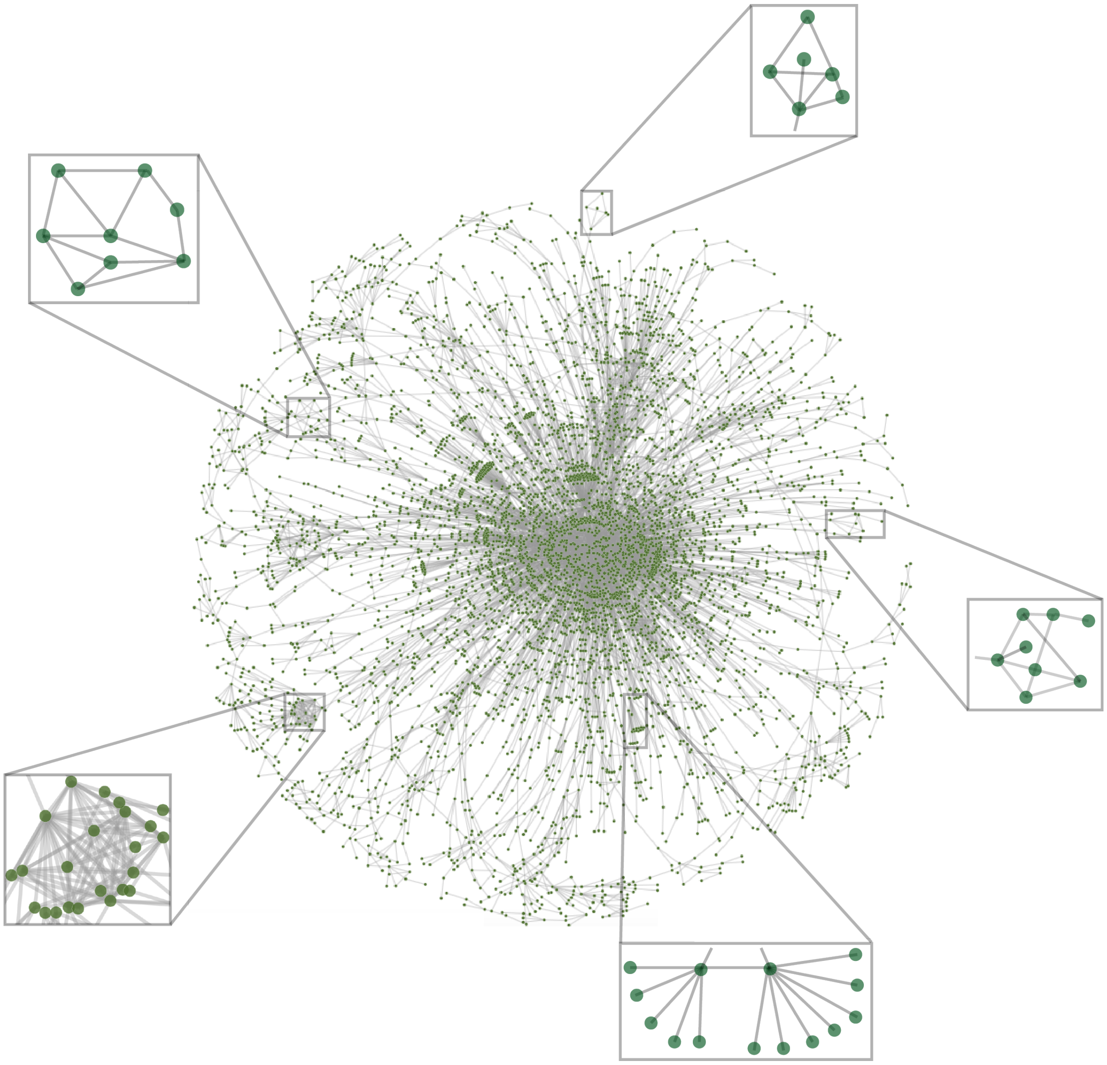

Supplementary Figure 1: A network visualization of a random 10% sample of the giant connected component of the network displayed using a force-directed graph drawing algorithm. Also shown are insets showing characteristic motifs of the network structure. The algorithm situates nodes of the graph in two-dimensional space so that all the edges are of more or less equal length and there are as few crossing edges as possible. This is achieved by assigning forces among the set of edges and the set of nodes and then using these forces either to simulate the motion of the edges and nodes or to minimize their energy (*1*).

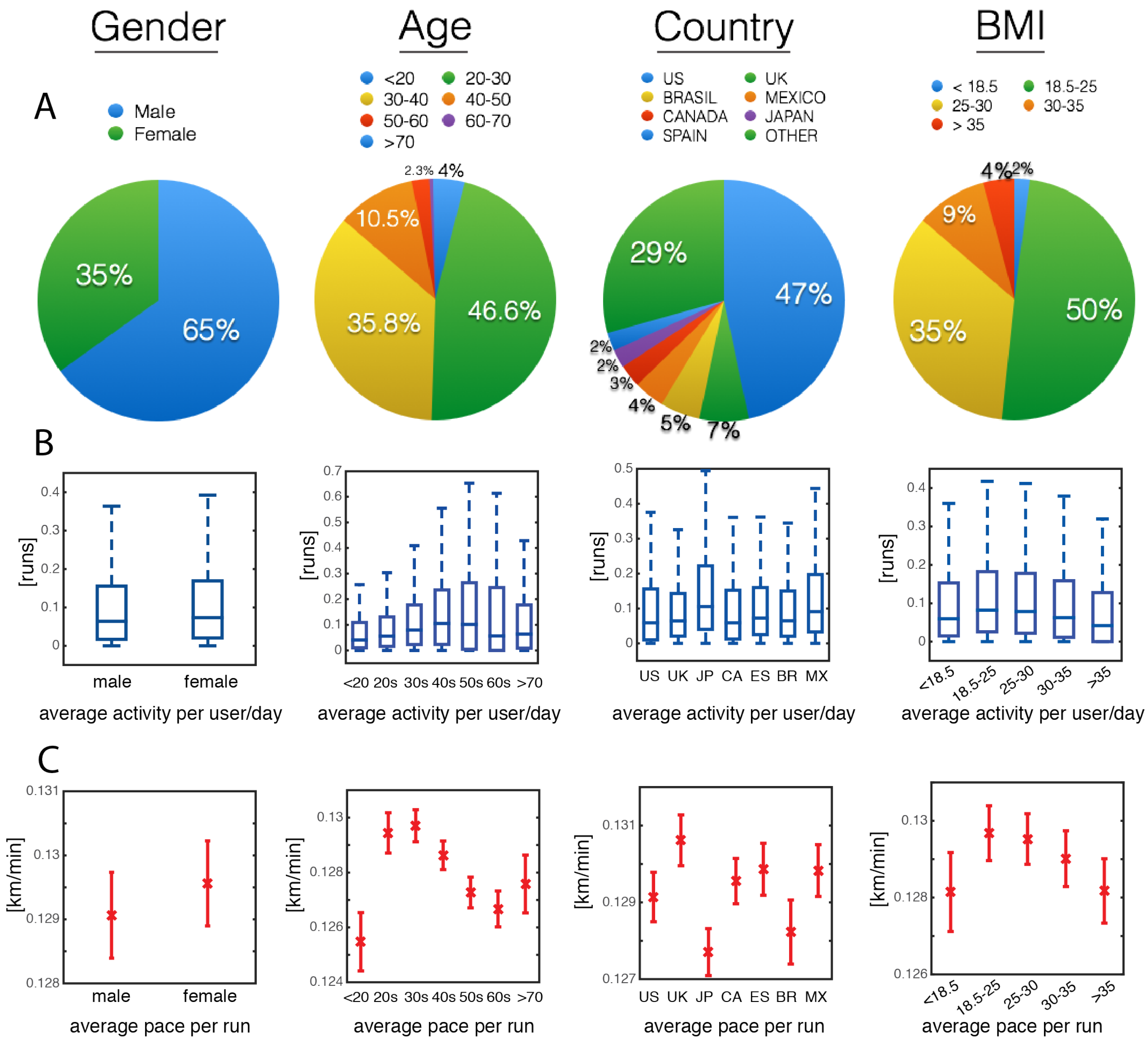

Supplementary Figure 2: (A) The demographic summary statistics of the ∼1.1 million network-embedded runners by gender, age, country and BMI at the time of registration. (B) The daily activity measured by the number of runs per day by different demographic categories displayed as PlotBoxs with demarcations for the minimum, first quartile, median, third quartile, and maximum. (C) The average pace per run (with 95% confidence intervals) for different demographic categories measured in kilometers per minute.

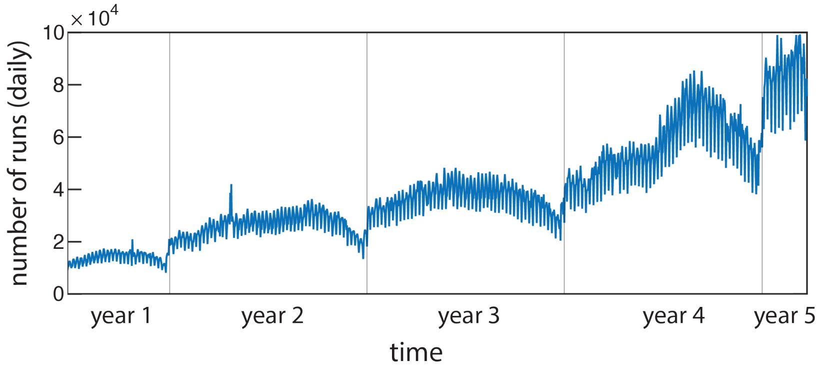

Supplementary Figure 3: Aggregated daily activity as a function of time (measured in total number of runs) of the network-embedded runners.

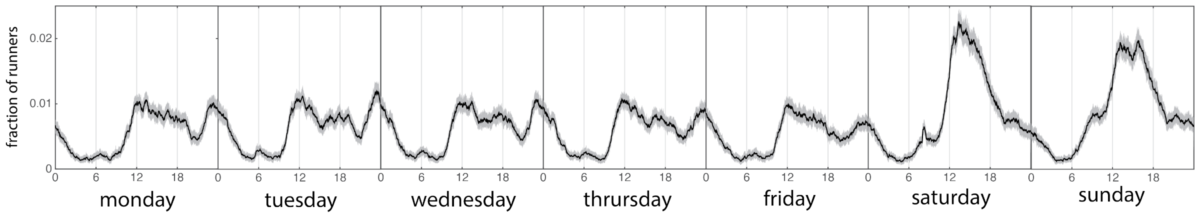

Supplementary Figure 4: The fraction of active runners in United States (number of people running divided by the total number of runners) for a period of one week.

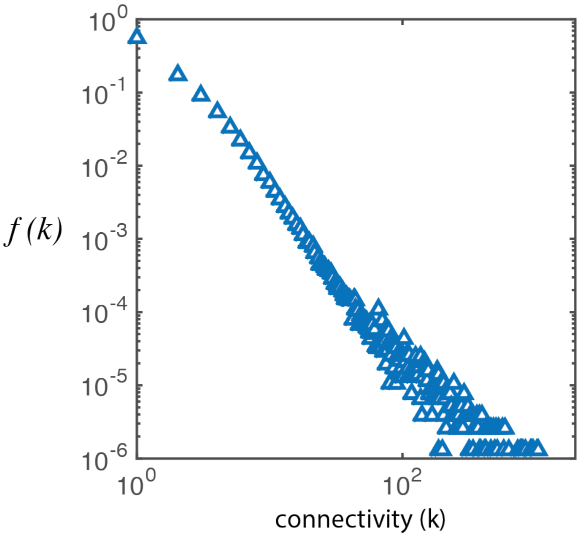

Supplementary Figure 5: The degree distribution $f(k)$ of the underlying running social network.

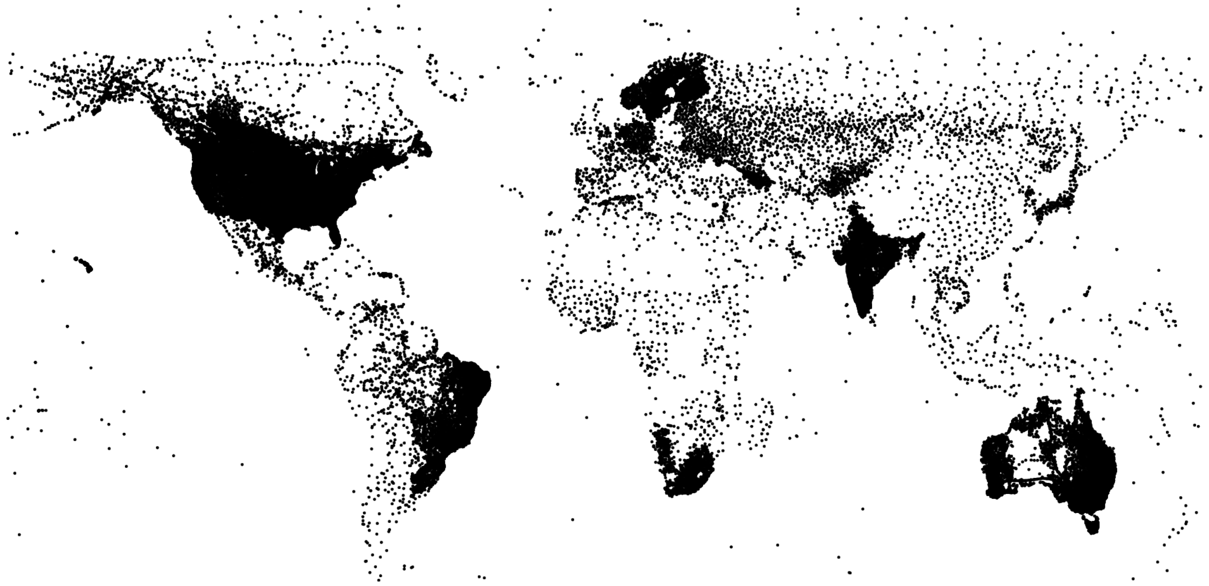

Supplementary Figure 6: The location of the almost 48K weather stations worldwide where weather data are available for the period of observation. The four countries with the largest number of weather towers are: the United States (32,643), Australia (7,069), Canada (2256) and Sweden (823). Even without geographic demarcations, a geographic plot of the weather stations gives us a well defined world map.

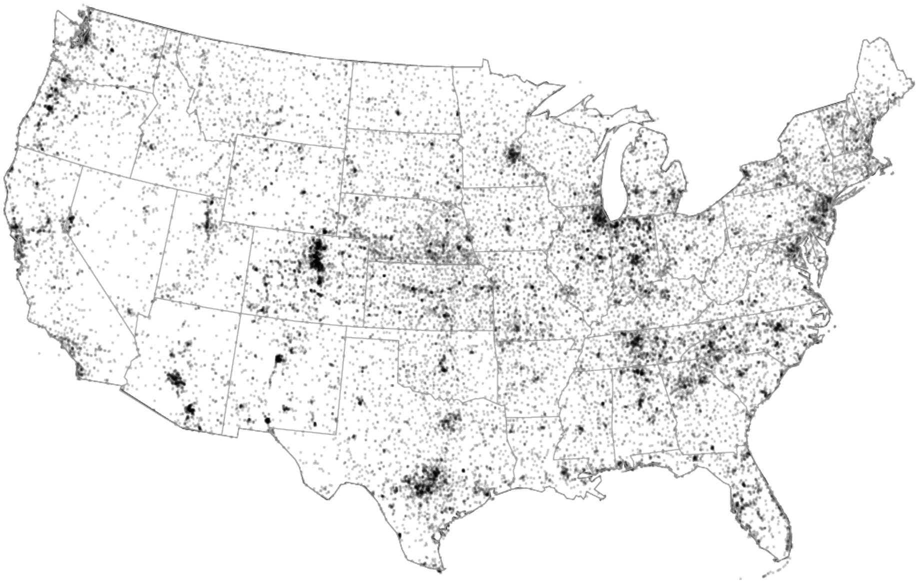

Supplementary Figure 7: The location of the $\sim$32K weather stations in the contiguous US.

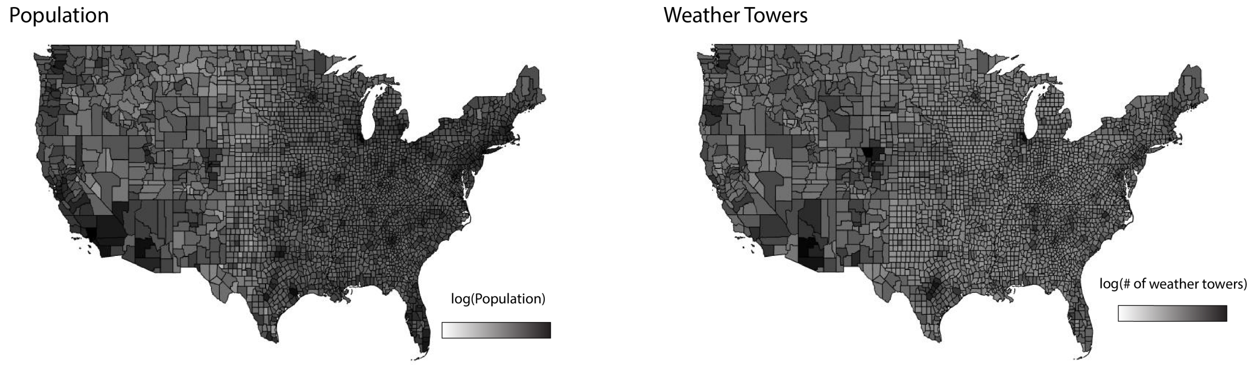

Supplementary Figure 8: The population distribution in the contiguous United States at the county level (left) along with the weather tower distribution (right). The correlation coefficient between the two distributions is ∼0.59, suggesting that the density of weather towers is well correlated with population density.

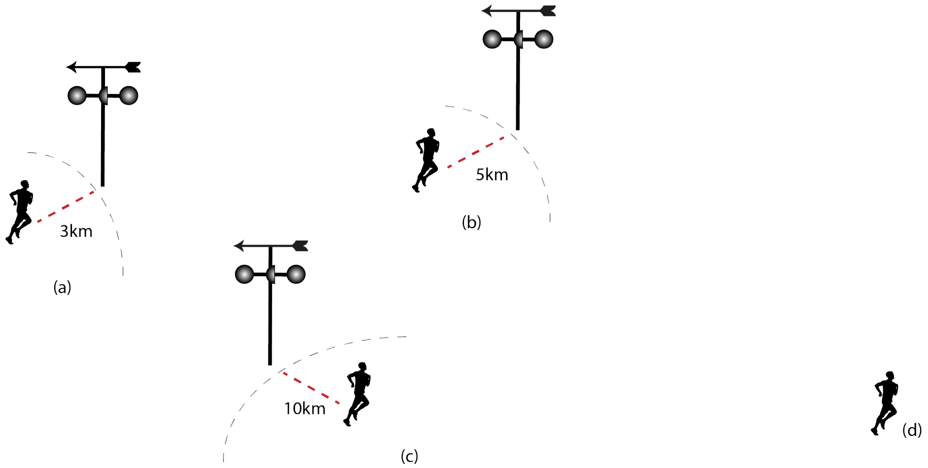

Supplementary Figure 9: Illustration of how we assign individuals to weather stations. For each individual we have their location either through GPS data or through the address they provide during registration on the website. At the same time we have information about the exact location of weather stations. The weather of the closest weather station to an individual is assigned as the weather the individual experiences. Individuals that are located further than 30Km away from their closest weather station are excluded since we are unable to precisely identify the weather they experience (e.g. individual d). The excluded group makes up approximately 3% of the sample.

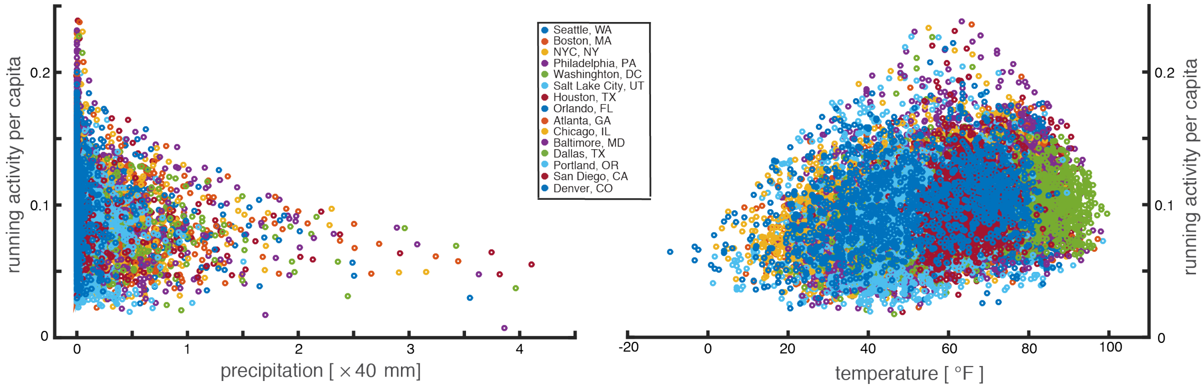

Supplementary Figure 10: Daily per capita running activity as a function of the daily precipitation (left) and average daily temperature (right) of the 15 largest cities in which these individuals run in the United States. The plot reveals a monotonic relationship between running and precipitation on one hand and an inverted-U shaped relationship between running and temperature on the other.

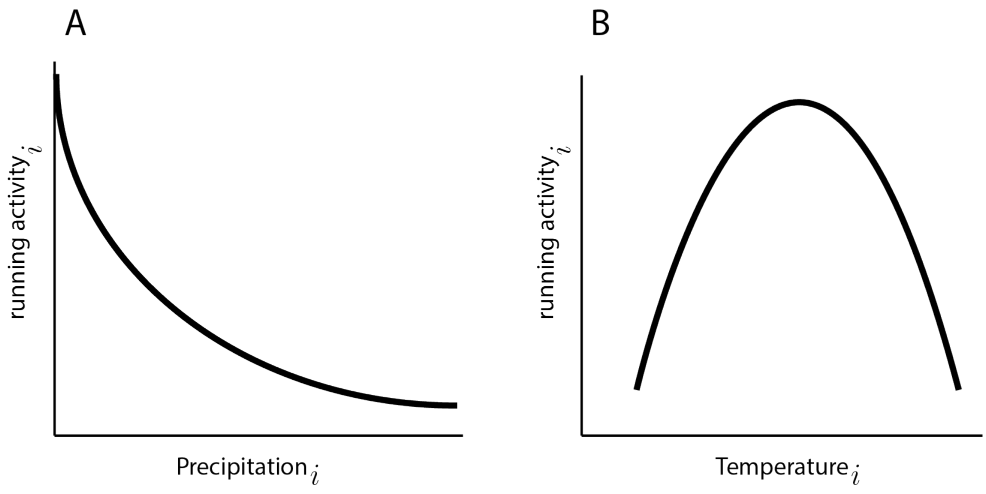

Supplementary Figure 11: More precipitation is monotonically associated with less running (see Figure 4C in the main manuscript). On the other hand, the relationship between running and temperature is non monotonic suggesting that very high and low temperatures are associated with less exercise activity (see Figure 4C in the main manuscript).

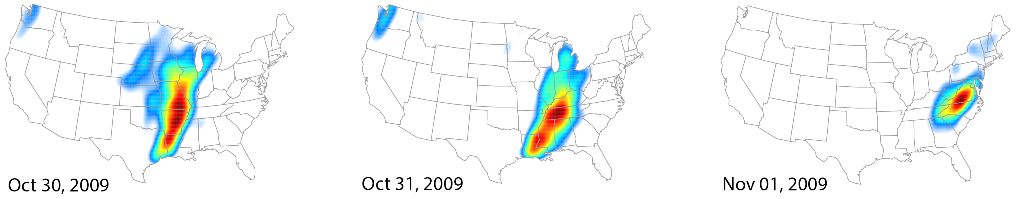

Supplementary Figure 12: A typical precipitation pattern over the Midwest and south of the U.S. as well as its temporal evolution over three consecutive days. Dark red colors indicate large amounts of precipitation.

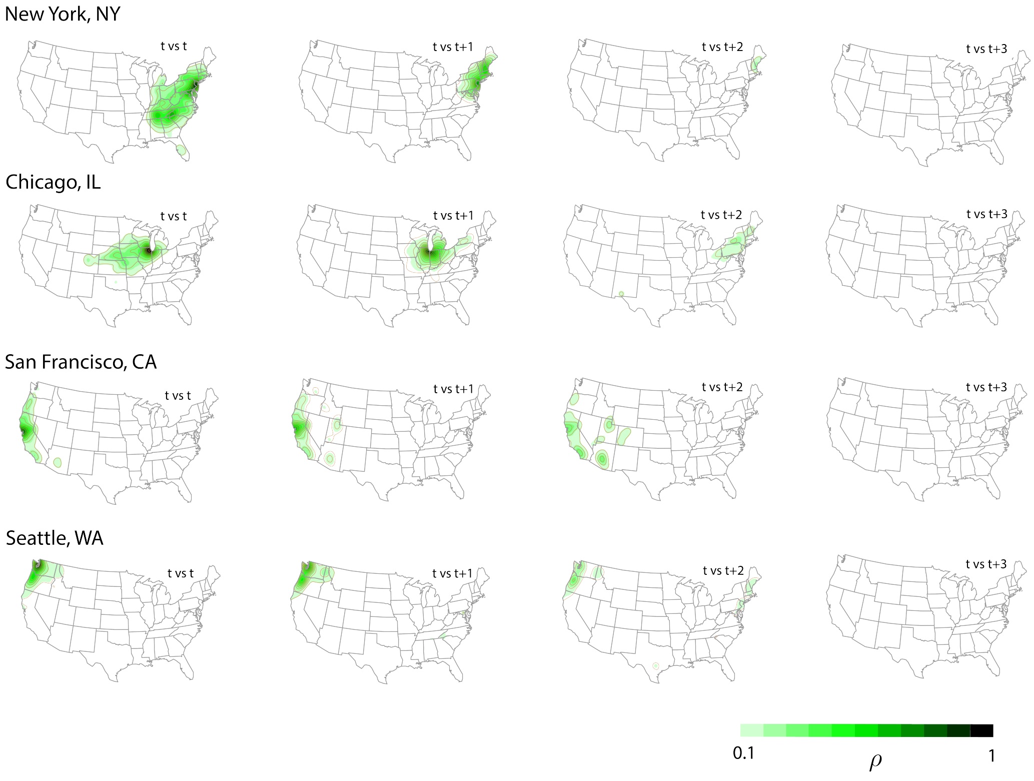

Supplementary Figure 13: The sample Pearson correlation coefficient between the weather in four large cities in US (New York, Chicago, San Francisco and Seattle) on day t and any other area in the contiguous US on the same day are displayed (left panel) next to the correlations of the weather in these cities on day t with the weather everywhere else in the US one (t vs $t+1$), two (t vs $t+2$), and three days (t vs $t+3$) later. Dark green colors represent correlations close to 1, light green colors represent correlations close to 0.1 and white colors represent correlations less than 0.1.

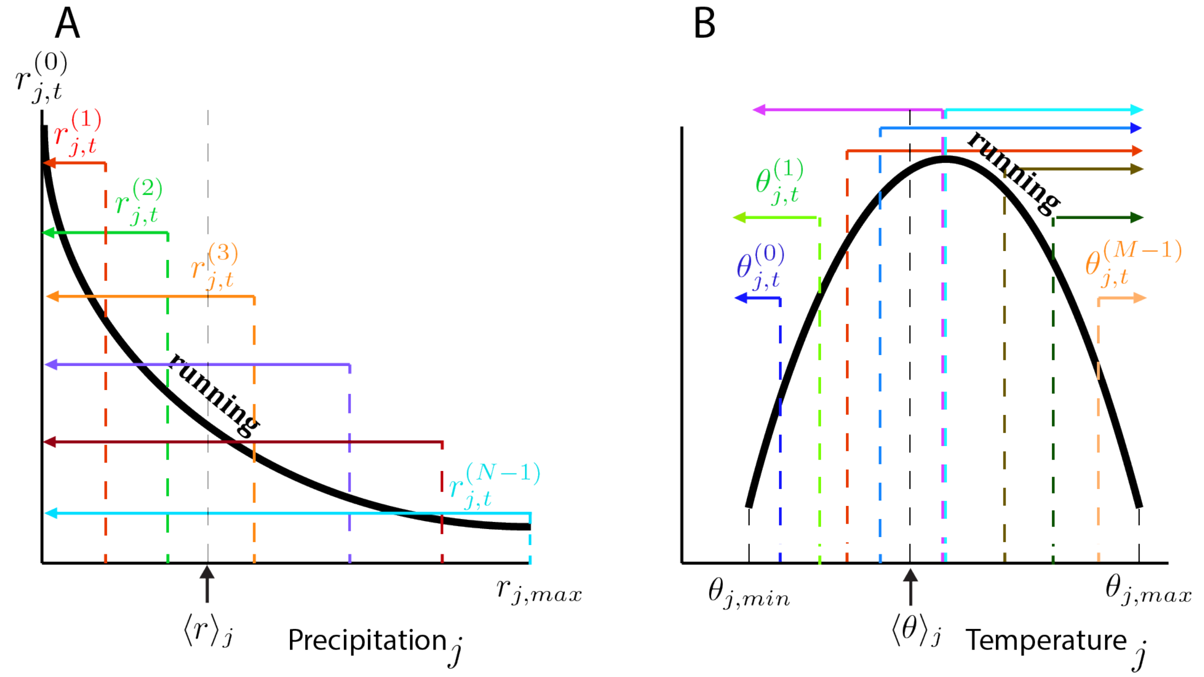

Supplementary Figure 14: Larger amounts of precipitation the peer experiences, are associated with lower running activity (A) while the relationship between running activity and temperature is non monotonic suggesting that very high and very low temperatures are associated with low exercise activity (B). We design N=12 rain and M=10 temperature binary indicators for the weather the peers' experience. For the rain binaries, we divide the range of precipitation i experiences into N percentiles and we define binaries so that $r_{j,t}^{(0)} = (r_{j,t} = 0)$, $r_{j,t}^{(1)} = (pr_{j,t} \leq \langle r \rangle_i/20)$, $r_{j,t}^{(2)} = (pr_{j,t} \leq \langle r \rangle_j/10)$, $r_{j,t}^{(3)} = (pr_{j,t} \leq \langle r \rangle_j/5)$, $r_{j,t}^{(4)} = (pr_{j,t} \leq \langle r \rangle_j/4)$, $r_{j,t}^{(5)} = (pr_{j,t} \leq \langle r \rangle_j/2)$, $r_{j,t}^{(6)} = (pr_{j,t} \leq \langle r \rangle_j)$, $r_{j,t}^{(7)} = (pr_{j,t} \leq \langle r \rangle_j * 1.5)$, $r_{j,t}^{(8)} = (pr_{j,t} \leq \langle r \rangle_j * 2)$, $r_{j,t}^{(9)} = (pr_{j,t} \leq \langle r \rangle_j * 5)$, $r_{j,t}^{(10)} = (pr_{j,t} \leq \langle r \rangle_j * 7)$, $r_{j,t}^{(11)} = (pr_{j,t} \leq \langle r \rangle_j * 10)$, where $pr_{j,t}$ is the amount of precipitation j experiences on day t and $\langle r_j \rangle$ is the average amount of precipitation i experiences for the period of observation. At the same time, for the temperature binaries, we design the binary indicators in a slightly more complicated way in order to be able to capture the nonlinear relationship between running and temperature. For each peer we define the minimum, maximum and average temperature that she/he experiences for the duration of observation and define the binary indicators so that $\theta_{j,t}^{(0)} = (\theta_{j,t} < \theta_{j,min} + \{\langle \theta \rangle_j - \theta_{j,min}\}/5)$, $\theta_{j,t}^{(1)} = (\theta_{j,t} < \theta_{j,min} + 2\{\langle \theta \rangle_j - \theta_{j,min}\}/5)$, $\theta_{j,t}^{(2)} = (\theta_{j,t} \geq \theta_{j,min} + 3.5\{\langle \theta \rangle_j - \theta_{j,min}\}/5)$, $\theta_{j,t}^{(3)} = (\theta_{j,t} \geq \theta_{j,min} + 4\{\langle \theta \rangle_j - \theta_{j,min}\}/5)$, $\theta_{j,t}^{(4)} = (\theta_{j,t} < \langle \theta \rangle_j$, $\theta_{j,t}^{(5)} = (\theta_{j,t} \geq \langle \theta \rangle_j$, $\theta_{j,t}^{(6)} = (\theta_{j,t} \geq \theta_{j,max} - \{\theta_{j,max} - \langle \theta \rangle_j\}/5)$, $\theta_{j,t}^{(7)} = (\theta_{j,t} \geq \theta_{j,max} - 2\{\theta_{j,max} - \langle \theta \rangle_j\}/5)$, $\theta_{j,t}^{(8)} = (\theta_{j,t} \geq \theta_{j,max} - 3\{\theta_{j,max} - \langle \theta \rangle_j\}/5)$, $\theta_{j,t}^{(9)} = (\theta_{j,t} \geq \theta_{j,max} - 4\{\theta_{j,max} - \langle \theta \rangle_j\}/5)$, where $\theta_{j,max}$ ($\theta_{j,min}$) is the maximum (minimum) and $\langle \theta \rangle_j$ is the average temperature that the peer j experiences throughout the course of observation.

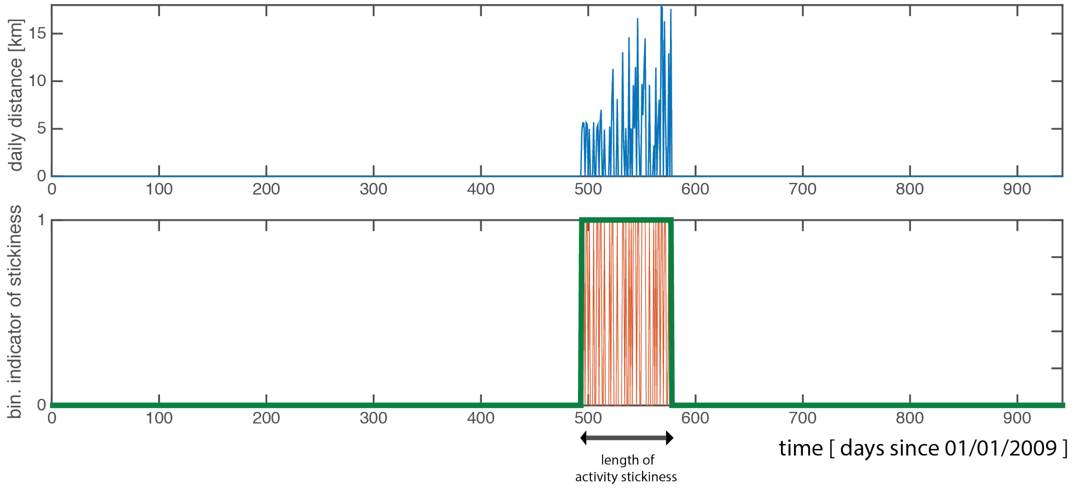

Supplementary Figure 15: Illustration of the methodology used to extract the training consistency of runners. Periods of consistent activity are defined as those during which no period of inactivity longer than two weeks exists.

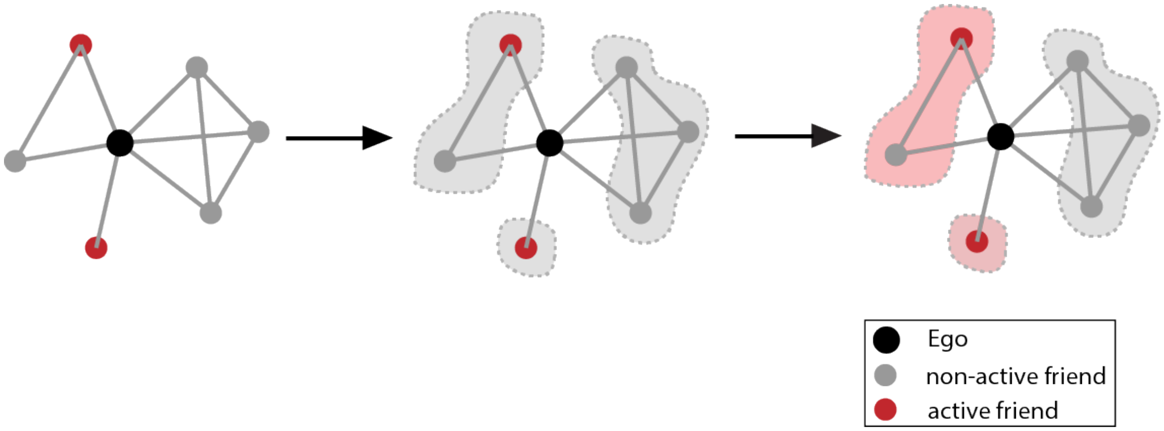

Supplementary Figure 16: Illustration of the methodology used to extract the number of active connected components a runner (Ego) has at each time. In this particular example an Ego i on day t has a neighborhood of 6 friends (2 of which are running) and 3 connected components (2 of which are active).

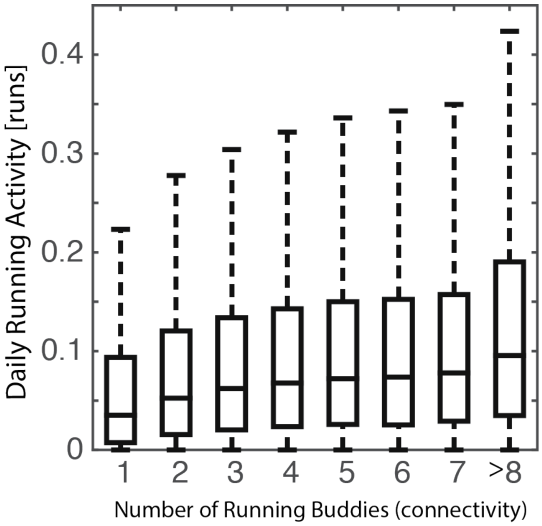

Supplementary Figure 17: Box plots with the minimum, first quartile, median, third quartile, and maximum of daily individual running activity, measured in number of runs, as a function of the number of friends someone has.

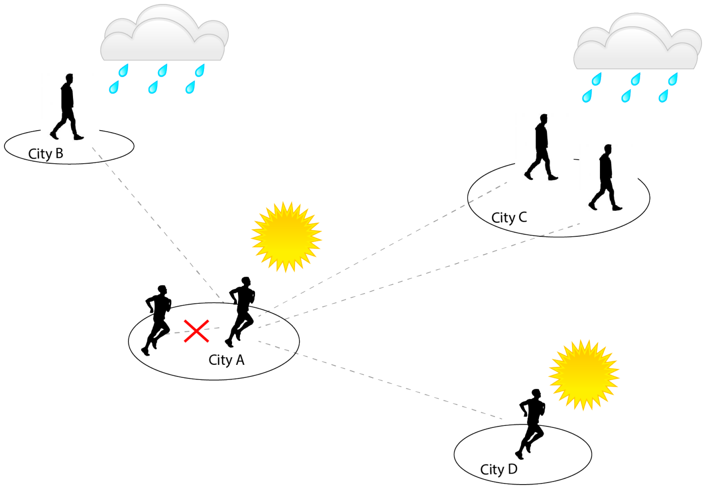

Supplementary Figure 18: Meeting the exclusion criterion. For each Ego i we exclude all the links to peers that share the same or highly correlated weather. Also to design the weather variables that can potentially serve as instruments for the peers' running activity we consider only the distinct number of towers that have different weather. For example, in the above illustration, Ego has five friends in four different cities. First, we remove links between Ego and the friends that they have in the same city. Furthermore, in order to design the variables that can serve as instruments, we use the weather of the three distinct cities in which Ego has peers (city B, city C and city D). The above methodology ensures that the exclusion criterion is not violated.

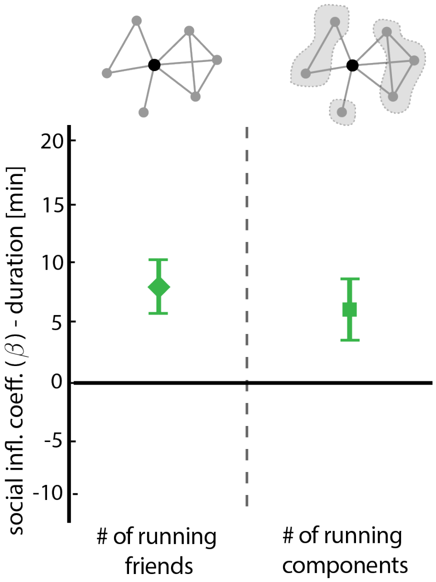

Supplementary Figure 19: (A) The social influence coefficient (measured in minutes) when we consider the number of active friends (diamonds) and the number of active connected components in Ego's neighborhood (squares) as the endogenous variables in *separate* regressions. Full results with diagnostic statistics are displayed in Supplementary Tables 14, 16

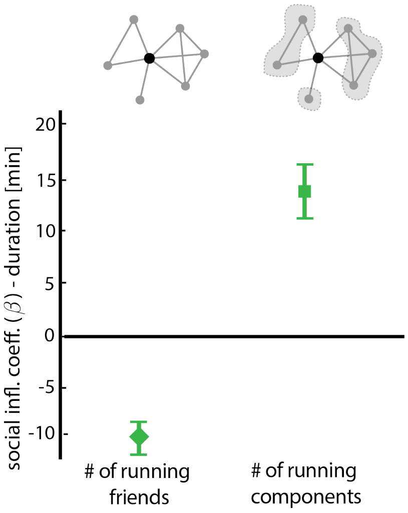

Supplementary Figure 20: The social influence coefficient for running duration (measured in minutes) when we consider the number of running friends (diamonds) and the number of running components (squares) in the same regression. Full results and regression diagnostics are displayed in Supplementary Table 17

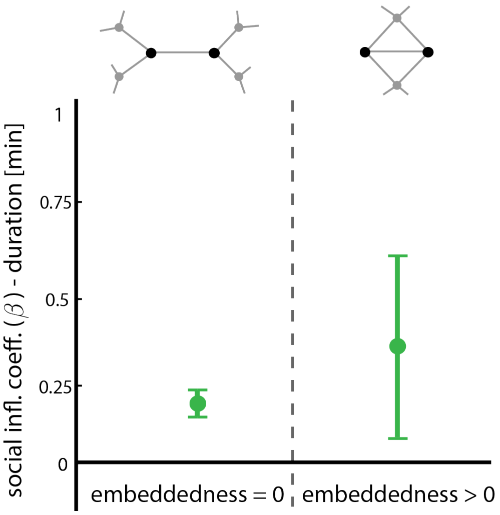

Supplementary Figure 21: The social influence coefficient when we consider duration as the running performance indicator when the links between Ego and peers are not embedded (left) and in the case when the links are embedded, i.e. Ego and peers share at least one common friend (right). Full results are displayed in Supplementary Table 14

.

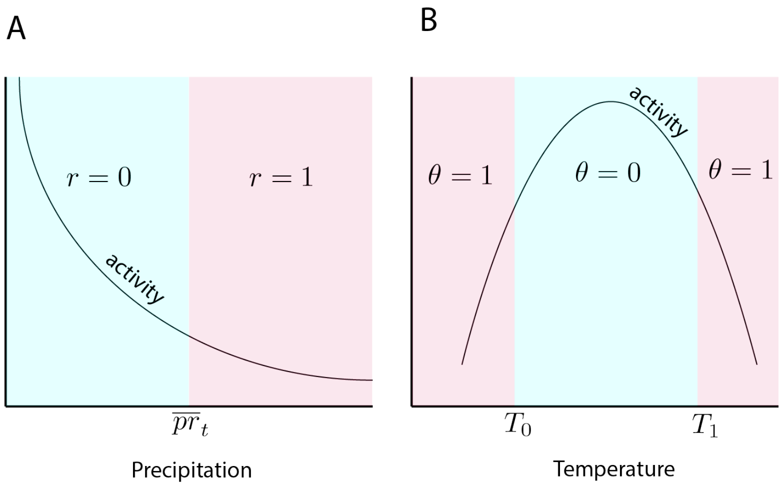

Supplementary Figure 22: The design of the two weather binary indicators. (A) For each individual j on each day t we consider a binary rain indicator r_{jt} that is equal to 1 if the precipitation individual j experiences on day t, pr_{jt}, is more than a seasonal average $\overline{pr}_{jt}$, and 0 otherwise. (B) We build a binary indicator for temperature $\theta_{j,t}$ that is equal to 1 if the temperature $T_{j,t}$ that individual j experiences is outside a normal temperature range (T_0, T_1)=(35,85)^{o}F and 0 otherwise. By design the two weather indicators are expected to negatively correlate with running activity.

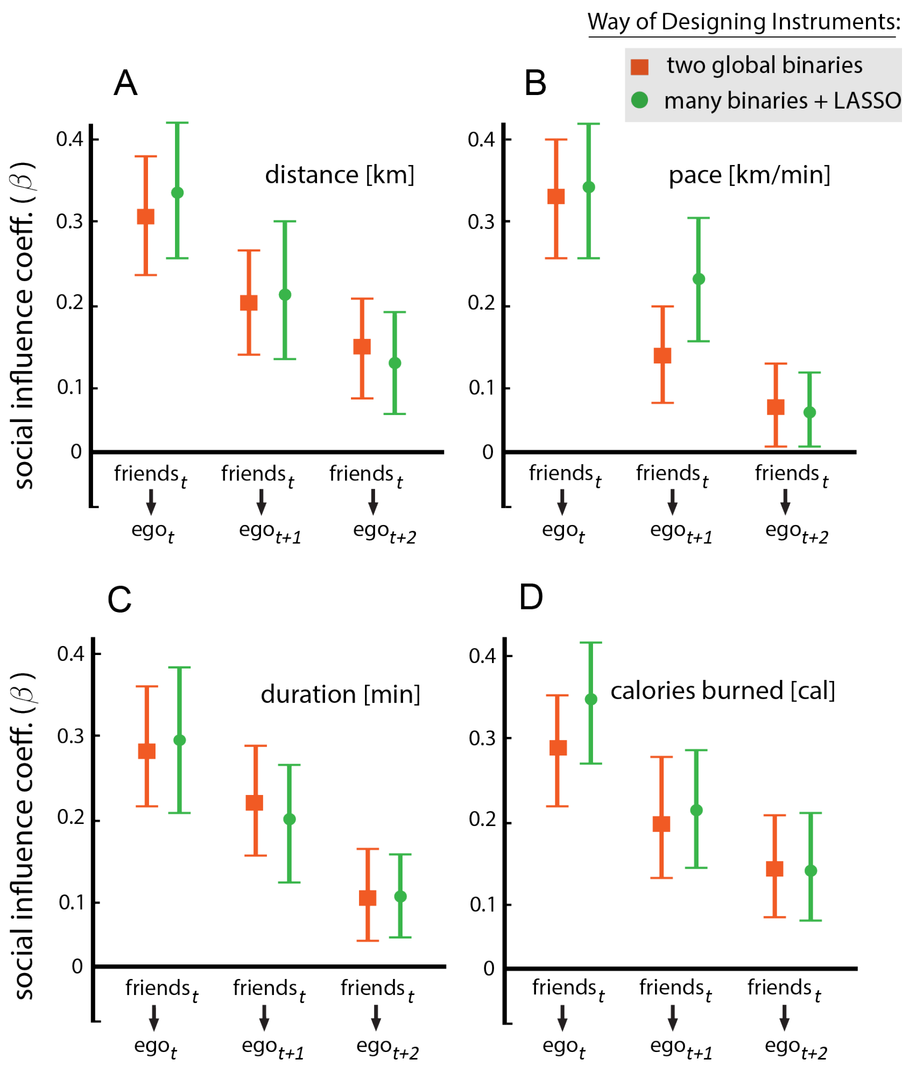

Supplementary Figure 23: The social influence coefficients β from the two stage least squares model (Supplementary Equation 2) for the case in which we use two global binary indicators for weather (R_{ft} and Θ_{ft}) as instruments, as well as the coefficients from the 2SLS model in which we design N+M binary weather indicators and use the LASSO method to identify the most suitable instruments as described in "First Stage Regressions" in Supplementary Note 4. Results are also displayed, with a full set of statistical diagnostic tests, in Supplementary Tables 20 to 23.

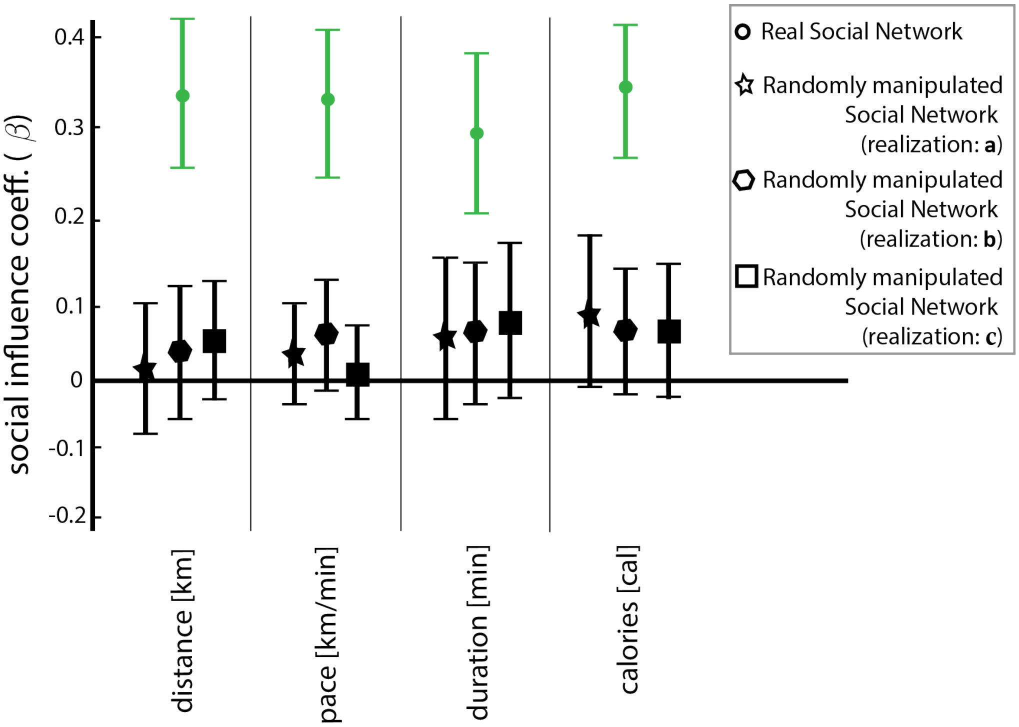

Supplementary Figure 24: The social influence coefficient for the four running performance indicators are shown when we consider the true underlying social network from the running network data (circles) as well as when we randomly manipulate the social network. We display results from three realizations of the network randomization in the figure. Full results are displayed in Supplementary Tables 25, 26 and 27

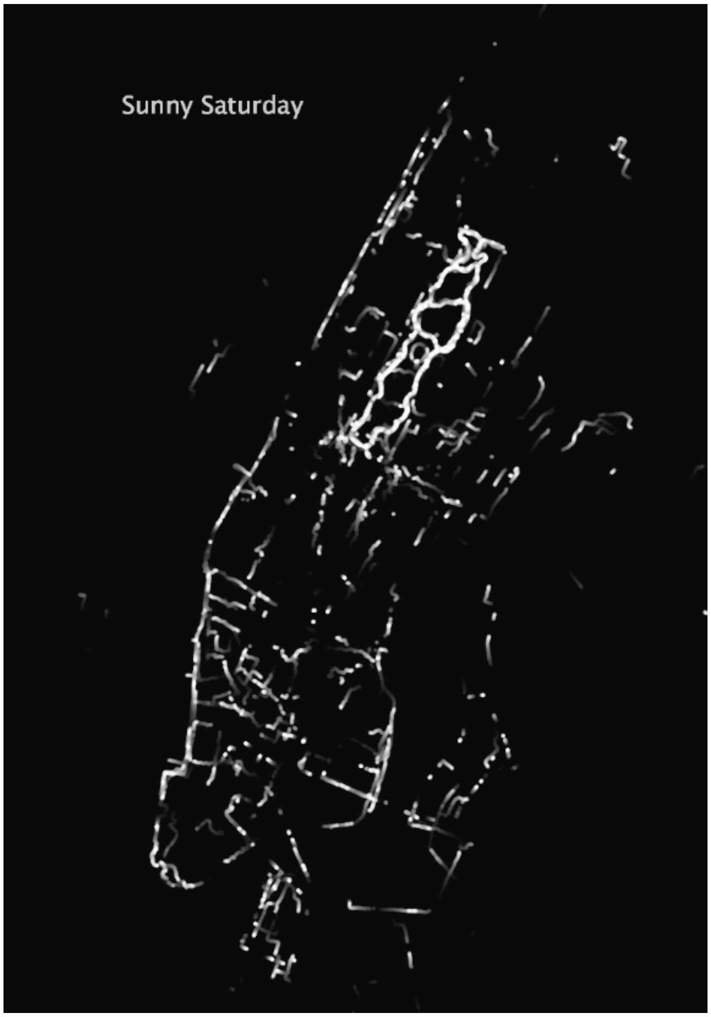

Supplementary Figure 25: Snapshot from Supplementary Movie 1. The GPS recorded running footprint of Manhattan during a *sunny* Saturday afternoon.

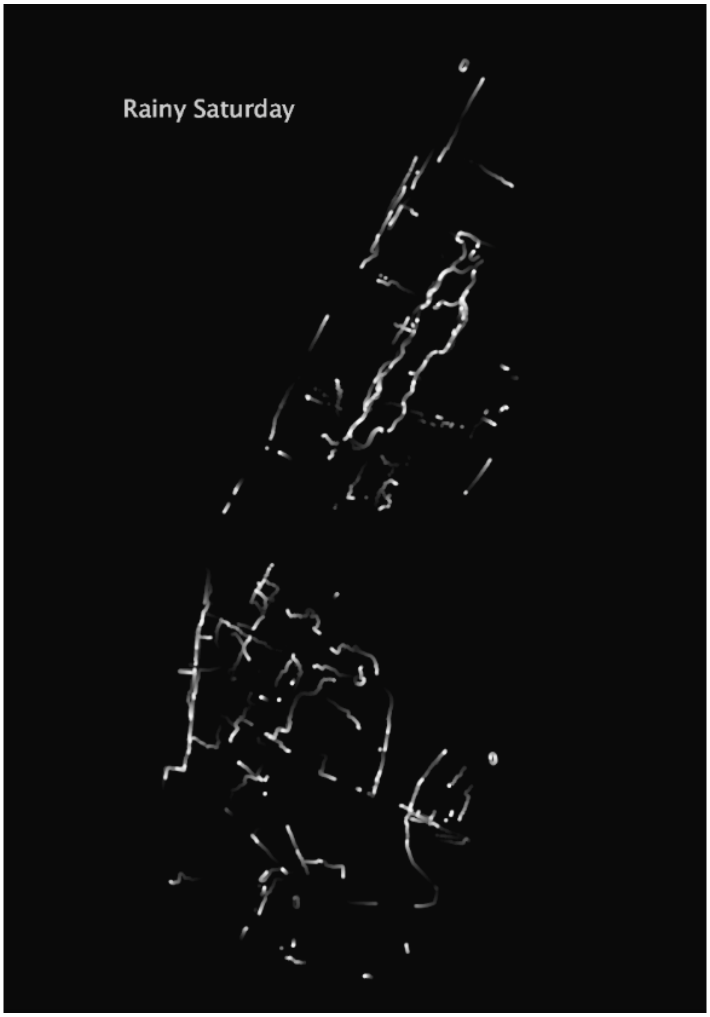

Supplementary Figure 26: Snapshot from Supplementary Movie 2. The GPS recorded running footprint of Manhattan during a *rainy* Saturday afternoon.

Supplementary Tables

demographic data

variable name	mean	S.D.	min	max
demographics				
age (years)	30.73	8.25	9	89
height (m)	1.73	0.10	1.27	2.29
weight (kg)	76.75	16.26	20.80	138.0
gender				
male (bin.)	0.6459		0	1
female (bin.)	0.3535		0	1
undefined (bin.)	0.0006		0	1
country				
USA (bin.)	0.4742		0	1
UK (bin.)	0.2877		0	1
Brasil (bin.)	0.0521		0	1
Mexico (bin.)	0.0371		0	1
Canada (bin.)	0.0327		0	1
Japan (bin.)	0.0235		0	1
Spain	0.0184		0	1
other	0.0743		0	1
running activity				
distance per run [km]	6.61	4.60	0.1	111
duration per run [min]	44.50	32.17	1.23	421.21
calories per run [cal]	489	373	33	4810
pace per run [km/min]	0.129	0.042	0.001	1.076

Supplementary Table 1: Demographics and running activity data for the 1.1 million individuals in the running social network.

weather data				
variable name	mean	S.D.	min	max
daily precipitation (mm)	40	11	0	179
daily temperature (°C)	22	8	-43	54.5

Supplementary Table 2: Weather data.

	New York NY(t)	Chicago IL(t)	Columbus OH(t)
New York NY(t)	1	0.02	0.23
Chicago IL(t)	0.02	1	0
Columbus OH(t)	0.23	0	1

Supplementary Table 3: Same day weather correlation coefficients between New York NY, Chicago IL and Columbus OH.

Identification Model: **Supplementary Equation 2**
Fitness Indicator : **Daily Distance [km]**

A_{it} Vs. $\bar{A}^p_{it}$

Instruments: **1.** $R_t^{(7)}$ **2.** $\Theta_t^{(2)}$

first stage					95% conf. interval	
Friends' Av. Activity $\bar{A}^p_{it}$	coefficient	std. error	t-value	$P > \|t\|$	low	high
Instrument 1	0.0124	0.0006	22.47	0.000	0.0113	0.0135
Instrument 2	0.0077	0.0003	24.14	0.000	0.0071	0.0083
second stage					95% conf. interval	
Ego' s Activity A_{it}	coefficient	std. error	t-value	$P > \|t\|$	low	high
Friends' Av. Activity $\bar{A}^p_{it}$	0.3425	0.0400	8.56	0.000	0.2640	0.4209

Total number of observations N=9,560,804. The Kleibergen-Paap rk LM statistic is 1392 (P = 0.0000) suggesting the regression is not underidentied. The Cragg-Donald Wald F statistics is 335 which exceeds the critical thresholds suggested by Stock and Yogo to ensure the instruments are not weak. The Wu-Hausmann F statistics is 75 (P=0.0000) which suggests that the friend's activity is endogenous. The Hansen overidentification restriction test gives p-value equal to P=0.2122 that fails to reject the null hypothesis for valid instruments.

$A_{i,t+1}$ Vs. $\bar{A}^p_{it}$

Instruments: **1.** $R_t^{(3)}$ **2.** $\Theta_t^{(3)}$

first stage					95% conf. interval	
Friends' Av. Activity $\bar{A}^p_{it}$	coefficient	std. error	t-value	$P > \|t\|$	low	high
Instrument 1	0.0122	0.0004	28.93	0.000	0.0114	0.0131
Instrument 2	0.0067	0.0002	26.70	0.000	0.0062	0.0072
second stage					95% conf. interval	
Ego' s Activity $A_{i,t+1}$	coefficient	std. error	t-value	$P > \|t\|$	low	high
Friends' Av. Activity $\bar{A}^p_{it}$	0.2143	0.0519	5.12	0.000	0.1284	0.3001

Total number of observations N=10,204,895. The Kleibergen-Paap rk LM statistic is 1901 (P = 0.0000) suggesting the regression is not underidentied. The Cragg-Donald Wald F statistics is 429 which exceeds the critical thresholds suggested by Stock and Yogo to ensure the instruments are not weak. The Wu-Hausmann F statistics is 47 (P=0.000) which suggests that the friend's activity is endogenous. The Hansen overidentification restriction test gives p-value equal to P=0.1992 that fails to reject the null hypothesis for valid instruments.

$A_{i,t+2}$ Vs. $\bar{A}^p_{it}$

Instruments: **1.** $R_t^{(0)}$ **2.** $\Theta_t^{(2)}$

first stage					95% conf. interval	
Friends' Av. Activity $\bar{A}^p_{it}$	coefficient	std. error	t-value	$P > \|t\|$	low	high
Instrument 1	0.0113	0.0005	19.47	0.000	0.0102	0.0124
Instrument 2	0.0066	0.0004	16.88	0.000	0.0058	0.0074
second stage					95% conf. interval	
Ego' s Activity $A_{i,t+2}$	coefficient	std. error	t-value	$P > \|t\|$	low	high
Friends' Av. Activity $\bar{A}^p_{it}$	0.1325	0.0337	3.93	0.000	0.0664	0.1987

Total number of observations N=12,186,406. The Kleibergen-Paap rk LM statistic is 574 (P = 0.0000) suggesting the regression is not underidentied. The Cragg-Donald Wald F statistics is 266 which exceeds the critical thresholds suggested by Stock and Yogo to ensure the instruments are not weak. The Wu-Hausmann F statistics is 29 (P=0.000) which suggests that the friend's activity is endogenous. The Hansen overidentification restriction test gives p-value equal to P=0.1888 that fails to reject the null hypothesis for valid instruments.

Supplementary Table 4: Ego Influence Identification Results (Ego ID fixed effects) – Daily Distance. Results are displaying in Figure 1 (top-left panel) of the main manuscript.

Identification Model: **Supplementary Equation 2**
Fitness Indicator : **Daily Pace [km/min]**

A_{it} Vs. $\bar{A}^p_{it}$

Instruments: **1.** $R^{(3)}_t$ **2.** $\Theta^{(3)}_t$

					95% conf. interval	
first stage						
Friends' Av. Activity $\bar{A}^p_{it}$	coefficient	std. error	t-value	$P > \|t\|$	low	high
Instrument 1	0.0002	0.0000	23.74	0.000	0.0002	0.0002
Instrument 2	0.0001	0.0000	23.74	0.000	-0.0001	-0.0001
second stage					95% conf. interval	
Ego' s Activity A_{it}	coefficient	std. error	t-value	$P > \|t\|$	low	high
Friends' Av. Activity $\bar{A}^p_{it}$	0.3412	0.0437	7.80	0.000	0.2555	0.4270

Total number of observations N=9,504,974. The Kleibergen-Paap rk LM statistic is 1285 (P = 0.0000) suggesting the regression is not underidentied. The Cragg-Donald Wald F statistics is 299 which exceeds the critical thresholds suggested by Stock and Yogo to ensure the instruments are not weak. The Wu-Hausmann F statistics is 52 (P=0.0000) which suggests that the friend's activity is endogenous. The Hansen overidentification restriction test gives p-value equal to P=0.1432 that fails to reject the null hypothesis for valid instruments.

$A_{i,t+1}$ Vs. $\bar{A}^p_{it}$

Instruments: **1.** $R^{(3)}_t$ **2.** $\Theta^{(3)}_t$

					95% conf. interval	
first stage						
Friends' Av. Activity $\bar{A}^p_{it}$	coefficient	std. error	t-value	$P > \|t\|$	low	high
Instrument 1	0.0002	0.0000	29.22	0.000	-0.0012	-0.0010
Instrument 2	0.0001	0.0000	23.11	0.000	0.0001	0.0001
second stage					95% conf. interval	
Ego' s Activity $A_{i,t+1}$	coefficient	std. error	t-value	$P > \|t\|$	low	high
Friends' Av. Activity $\bar{A}^p_{it}$	0.2398	0.0381	6.00	0.000	0.1822	0.3073

Total number of observations N=10,580,279. The Kleibergen-Paap rk LM statistic is 779 (P = 0.0000) suggesting the regression is not underidentied. The Cragg-Donald Wald F statistics is 366 which exceeds the critical thresholds suggested by Stock and Yogo to ensure the instruments are not weak. The Wu-Hausmann F statistics is 68 (P=0.000) which suggests that the friend's activity is endogenous. The Hansen overidentification restriction test gives p-value equal to P=0.1319 that fails to reject the null hypothesis for valid instruments.

$A_{i,t+2}$ Vs. $\bar{A}^p_{it}$

					95% conf. interval	
first stage						
Friends' Av. Activity $\bar{A}^p_{it}$	coefficient	std. error	t-value	$P > \|t\|$	low	high
Friends' Tot. Rainfall $R_{f,t}$	-0.0010	0.0000	-21.34	0.000	-0.0010	-0.0009
Friends' Tot. Temper. $\Theta_{f,t}$	-0.0001	0.0000	-4.51	0.000	-0.0002	-0.0001
second stage					95% conf. interval	
Ego' s Activity $A_{i,t+2}$	coefficient	std. error	t-value	$P > \|t\|$	low	high
Friends' Av. Activity $\bar{A}^p_{it}$	0.072	0.03199	2.17	0.000	0.012	0.1358

Total number of observations N=11,988,434. The Kleibergen-Paap rk LM statistic is 458 (P = 0.0000) suggesting the regression is not underidentied. The Cragg-Donald Wald F statistics is 216 which exceeds the critical thresholds suggested by Stock and Yogo to ensure the instruments are not weak. The Wu-Hausmann F statistics is 39 (P=0.000) which suggests that the friend's activity is endogenous. The Hansen overidentification restriction test gives p-value equal to P=0.1222 that fails to reject the null hypothesis for valid instruments.

Supplementary Table 5: Ego Influence Identification Results (Ego ID fixed effects) – Daily Pace. Results are displaying in Figure 1 (top-right panel) of the main manuscript.

Identification Model: **Supplementary Equation 2**
Fitness Indicator : **Daily Duration [min]**

A_{it} Vs. $\bar{A}_{it}^{p}$

Instruments: **1.** $R_t^{(7)}$ **2.** $\Theta_t^{(3)}$

first stage					95% conf. interval	
Friends' Av. Activity $\bar{A}_{it}^{p}$	coefficient	std. error	t-value	$P > \|t\|$	low	high
Instrument 1	0.0873	0.0044	19.77	0.000	0.0786	0.0959
Instrument 2	0.0582	0.0021	27.27	0.000	0.0540	0.0624
second stage					95% conf. interval	
Ego' s Activity A_{it}	coefficient	std. error	t-value	$P > \|t\|$	low	high
Friends' Av. Activity $\bar{A}_{it}^{p}$	0.2950	0.0438	6.72	0.000	0.2087	0.3804

Total number of observations N=9,560,779. The Kleibergen-Paap rk LM statistic is 560 (P = 0.0000) suggesting the regression is not underidentied. The Cragg-Donald Wald F statistics is 321 which exceeds the critical thresholds suggested by Stock and Yogo to ensure the instruments are not weak. The Wu-Hausmann F statistics is 63 (P=0.0000) which suggests that the friend's activity is endogenous. The Hansen overidentification restriction test gives p-value equal to P=0.2177 that fails to reject the null hypothesis for valid instruments.

$A_{i,t+1}$ Vs. $\bar{A}_{it}^{p}$

Instruments: **1.** $R_t^{(3)}$ **2.** $\Theta_t^{(3)}$

first stage					95% conf. interval	
Friends' Av. Activity $\bar{A}_{it}^{p}$	coefficient	std. error	t-value	$P > \|t\|$	low	high
Instrument 1	0.0872	0.0034	25.42	0.000	0.0805	0.0939
Instrument 2	0.0560	0.0020	27.21	0.000	0.0520	0.0601
second stage					95% conf. interval	
Ego' s Activity $A_{i,t+1}$	coefficient	std. error	t-value	$P > \|t\|$	low	high
Friends' Av. Activity $\bar{A}_{it}^{p}$	0.2009	0.0377	5.33	0.000	0.1270	0.2749

Total number of observations N=10,204,872. The Kleibergen-Paap rk LM statistic is 729 (P = 0.0000) suggesting the regression is not underidentied. The Cragg-Donald Wald F statistics is 364 which exceeds the critical thresholds suggested by Stock and Yogo to ensure the instruments are not weak. The Wu-Hausmann F statistics is 34 (P=0.0000) which suggests that the friend's activity is endogenous. The Hansen overidentification restriction test gives p-value equal to P=0.2311 that fails to reject the null hypothesis for valid instruments

$A_{i,t+2}$ Vs. $\bar{A}_{it}^{p}$

Instruments: **1.** $R_t^{(3)}$ **2.** $\Theta_t^{(3)}$

first stage					95% conf. interval	
Friends' Av. Activity $\bar{A}_{it}^{p}$	coefficient	std. error	t-value	$P > \|t\|$	low	high
Instrument 1	0.0870	0.0034	24.42	0.000	0.0799	0.0959
Instrument 2	0.0568	0.0020	26.21	0.000	0.0510	0.0612
second stage					95% conf. interval	
Ego' s Activity $A_{i,t+2}$	coefficient	std. error	t-value	$P > \|t\|$	low	high
Friends' Av. Activity $\bar{A}_{it}^{p}$	0.1094	0.0345	3.57	0.000	0.0417	0.1771

Total number of observations N=11,922,086. The Kleibergen-Paap rk LM statistic is 497 (P = 0.0000) suggesting the regression is not underidentied. The Cragg-Donald Wald F statistics is 255 which exceeds the critical thresholds suggested by Stock and Yogo to ensure the instruments are not weak. The Wu-Hausmann F statistics is 36 (P=0.000) which suggests that the friend's activity is endogenous. The Hansen overidentification restriction test gives p-value equal to P=0.2009 that fails to reject the null hypothesis for valid instruments.

Supplementary Table 6: Ego Influence Identification Results (Ego ID fixed effects) – Daily Duration. Results are displaying in Figure 1 (bottom-left panel) of the main manuscript.

Identification Model: **Supplementary Equation 2**
Fitness Indicator : **Daily Calories Burned [cal]**

A_{it} Vs. $\bar{A}_{it}^{p}$

Instruments: **1.** $R_t^{(7)}$ **2.** $\Theta_t^{(3)}$

					95% conf. interval	
first stage						
Friends' Av. Activity $\bar{A}_{it}^{p}$	coefficient	std. error	t-value	$P > \|t\|$	low	high
Instrument 1	0.9469	0.0414	22.86	0.000	0.8657	1.0280
Instrument 2	0.5390	0.0197	27.27	0.000	0.5002	0.5777
second stage					95% conf. interval	
Ego' s Activity A_{it}	coefficient	std. error	t-value	$P > \|t\|$	low	high
Friends' Av. Activity $\bar{A}_{it}^{p}$	0.3501	0.0400	8.75	0.000	0.2716	0.4284

Total number of observations N=9,560,256. The Kleibergen-Paap rk LM statistic is 670 (P = 0.0000) suggesting the regression is not underidentied. The Cragg-Donald Wald F statistics is 335 which exceeds the critical thresholds suggested by Stock and Yogo to ensure the instruments are not weak. The Wu-Hausmann F statistics is 79 (P=0.0000) which suggests that the friend's activity is endogenous. The Hansen overidentification restriction test gives p-value equal to P=0.1655 that fails to reject the null hypothesis for valid instruments.

$A_{i,t+1}$ Vs. $\bar{A}_{it}^{p}$

Instruments: **1.** $R_t^{(3)}$ **2.** $\Theta_t^{(3)}$

					95% conf. interval	
first stage						
Friends' Av. Activity $\bar{A}_{it}^{p}$	coefficient	std. error	t-value	$P > \|t\|$	low	high
Instrument 1	0.9147	0.0318	28.71	0.000	0.8523	0.9772
Instrument 2	0.5214	0.0191	27.26	0.000	0.4838	0.5588
second stage					95% conf. interval	
Ego' s Activity $A_{i,t+1}$	coefficient	std. error	t-value	$P > \|t\|$	low	high
Friends' Av. Activity $\bar{A}_{it}^{p}$	0.2244	0.0342	6.56	0.000	0.1573	0.2915

Total number of observations N=10,204,310. The Kleibergen-Paap rk LM statistic is 861 (P = 0.0000) suggesting the regression is not underidentied. The Cragg-Donald Wald F statistics is 430 which exceeds the critical thresholds suggested by Stock and Yogo to ensure the instruments are not weak. The Wu-Hausmann F statistics is 51 (P=0.0000) which suggests that the friend's activity is endogenous. The Hansen overidentification restriction test gives p-value equal to P=0.1428 that fails to reject the null hypothesis for valid instruments.

$A_{i,t+2}$ Vs. $A_{i,t}$

Instruments: **1.** $R_t^{(3)}$ **2.** $\Theta_t^{(3)}$

					95% conf. interval	
first stage						
Friends' Av. Activity $\bar{A}_{it}^{p}$	coefficient	std. error	t-value	$P > \|t\|$	low	high
Instrument 1	0.9148	0.0319	26.75	0.000	0.8503	0.9791
Instrument 2	0.5210	0.0193	25.16	0.000	0.4812	0.5605
second stage					95% conf. interval	
Ego' s Activity $A_{i,t+2}$	coefficient	std. error	t-value	$P > \|t\|$	low	high
Friends' Av. Activity $\bar{A}_{it}^{p}$	0.1474	0.0348	4.24	0.000	0.0793	0.2157

Total number of observations N=12,109,634. The Kleibergen-Paap rk LM statistic is 620 (P = 0.0000) suggesting the regression is not underidentied. The Cragg-Donald Wald F statistics is 284 which exceeds the critical thresholds suggested by Stock and Yogo to ensure the instruments are not weak. The Wu-Hausmann F statistics is 37 (P=0.000) which suggests that the friend's activity is endogenous. The Hansen overidentification restriction test gives p-value equal to P=0.1215 that fails to reject the null hypothesis for valid instruments.

Supplementary Table 7: Ego Influence Identification Results (Ego ID fixed effects) – Daily Calories Burned. Results are displaying in Figure 1 (bottom-right panel) of the main manuscript.

A_{it} Vs. $\bar{A}^p_{it}$	IV model	OLS model	OLS overestimate
distance	0.34 (0.04)	0.62 (0.08)	81%
pace	0.34 (0.04)	0.61 (0.08)	78%
duration	0.29 (0.04)	0.53 (0.07)	80%
calories	0.35 (0.04)	0.60 (0.08)	72%

The p-values for all the results in the table are $p < 0.001$, N==9,560,779 observations.

Supplementary Table 8: The IV estimations are compared with the estimations from the corresponding OLS model as described in “Comparison of IV Estimates with an OLS Model” in Supplementary Note 3

Identification Model: **Supplementary Equation 5**
Fitness Indicator : **Daily Distance [km]**
Instruments: **Interactions between $R_t^{(7)}$ and $\nu_{ij} = A_j/A_i$**

<u>*second stage results*</u>

Ego' s Activity A_{it}

	coefficient	std. error	t-value	$P > \|t\|$	95% conf. interval low	high
$A_j/A_i \leq 1/16$						
Friends' Activity $\bar{A}_{it}^p$	0.3968	0.1493	2.35	0.021	0.0354	0.7771
$1/16 < A_j/A_i \leq 1/16$						
Friends' Activity $\bar{A}_{it}^p$	0.4452	0.1304	3.41	0.001	0.1896	0.7007
$1/8 < A_j/A_i \leq 1/4$						
Friends' Activity $\bar{A}_{it}^p$	0.5244	0.0702	7.47	0.000	0.3867	0.6620
$1/4 < A_j/A_i \leq 1/16$						
Friends' Activity $\bar{A}_{it}^p$	0.2040	0.0554	3.68	0.000	0.0954	0.3125
$1/2 < A_j/A_i \leq 2$						
Friends' Activity $\bar{A}_{it}^p$	0.11279	0.0314	3.59	0.000	0.0511	0.1743
$2 < A_j/A_i \leq 4$						
Friends' Activity $\bar{A}_{it}^p$	0.0439	0.0210	2.09	0.037	0.0026	0.0851
$4 < A_j/A_i \leq 8$						
Friends' Activity $\bar{A}_{it}^p$	0.0177	0.0222	0.80	0.425	-0.0259	0.0613
$8 < A_j/A_i \leq 16$						
Friends' Activity $\bar{A}_{it}^p$	0.0087	0.0288	0.30	0.761	-0.0476	0.0651
$A_j/A_i > 16$						
Friends' Activity $\bar{A}_{it}^p$	0.0238	0.0507	0.47	0.638	-0.0156	0.2233

Total number of observations N=14,105,729. The Kleibergen-Paap rk LM statistic is 1273 (P = 0.0000) suggesting the regression is not underidentied. The Cragg-Donald Wald F statistics is 598 which exceeds the critical thresholds suggested by Stock and Yogo to ensure the instruments are not weak. The Wu-Hausmann F statistics is 121 (P=0.0000) which suggests that the friend's activity is endogenous.

Supplementary Table 9: Results of the second stage of the interaction model in Supplementary Equation 5. The same results are graphically displayed in Figure 2A of the main manuscript.

Identification Model: **Supplementary Equation 6**
Fitness Indicator : **Daily Distance [km]**
Instruments: **Interactions between 1.**$R_t^{(7)}$**, 2.**$\Theta_t^{(2)}$ **and** $ac_i \in \{0, 1\}$

second stage results

Ego' s Activity A_{it}

	coefficient	std. error	t-value	$P > \|t\|$	95% conf. interval low	high
Ego Active / Friends Inactive Friends' Activity $\bar{A}_{it}^p$	1.597	0.0518	32.72	0.000	1.4952	1.6986
Ego Active / Friends Active Friends' Activity $\bar{A}_{it}^p$	0.4859	0.0114	42.44	0.000	0.4635	0.5083
Ego Inactive / Friends Inactive Friends' Activity $\bar{A}_{it}^p$	0.2386	0.0380	6.27	0.000	0.1640	0.3132
Ego Inactive / Friends Active Friends' Activity $\bar{A}_{it}^p$	0.1888	0.1921	0.98	0.326	-0.1278	0.5055

Total number of observations N=10,980,058. The Kleibergen-Paap rk LM statistic is 1154 (P = 0.0000) suggesting the regression is not underidentied. The Cragg-Donald Wald F statistics is 521 which exceeds the critical thresholds suggested by Stock and Yogo to ensure the instruments are not weak. The Wu-Hausmann F statistics is 232 (P=0.0000) which suggests that the friend's activity is endogenous. The Hansen overidentification restriction test gives p-value equal to P=0.2121 that fails to reject the null hypothesis for valid instruments.

Supplementary Table 10: Results of the second stage of the interaction model in Supplementary Equation 6. The same results are graphically displayed in Figure 2B of the main manuscript.

Identification Model: **Supplementary Equation 7**
Fitness Indicator : **Daily Distance [km]**
Instruments: **Interactions between 1.$R_t^{(7)}$, 2.$\Theta_t^{(2)}$ and $c_i \in \{0,1\}$**

second stage results

Ego' s Activity A_{it}

	coefficient	std. error	t-value	$P > \|t\|$	95% conf. interval low	high
Ego Cons. / Friend InCons. Friends' Activity $\bar{A}_{it}^p$	0.932	0.2194	4.28	0.000	0.595	1.201
Ego InCons. / Friend InCons. Friends' Activity $\bar{A}_{it}^p$	0.2485	0.0958	4.55	0.000	0.1870	0.4234
Ego Cons. / Friend Cons. Friends' Activity $\bar{A}_{it}^p$	0.2218	0.0567	8.33	0.000	0.1900	0.2517
Ego InCons. / Friend Cons. Friends' Activity $\bar{A}_{it}^p$	0.049	0.0679	1.08	0.085	-0.0021	0.0721

Total number of observations N=11,589,142. The Kleibergen-Paap rk LM statistic is 1032 (P = 0.0000) suggesting the regression is not underidentied. The Cragg-Donald Wald F statistics is 544 which exceeds the critical thresholds suggested by Stock and Yogo to ensure the instruments are not weak. The Wu-Hausmann F statistics is 192 (P=0.0000) which suggests that the friend's activity is endogenous. The Hansen overidentification restriction test gives p-value equal to P=0.2323 that fails to reject the null hypothesis for valid instruments.

Supplementary Table 11: Results of the second stage of the interaction model in Supplementary Equation 7. The same results are graphically displayed in Figure 2C of the main manuscript.

Identification Model: **Supplementary Equation 8**
Fitness Indicator : **Daily Distance [km]**
Instruments: **Interactions between 1.**$R_t^{(0)}$ **and** $g_i \in \{male, female\}$

<u>*second stage results*</u>

Ego' s Activity A_{it}

	coefficient	std. error	t-value	$P > \|t\|$	95% conf. interval low	high
Ego male / Friends male<br>Friends' Activity $\bar{A}^p_{it}$	0.3289	0.0518	6.35	0.000	0.2273	0.4304
Ego male / Friends female<br>Friends' Activity $\bar{A}^p_{it}$	0.1859	0.0460	4.04	0.000	0.0957	0.2760
Ego female / Friends female<br>Friends' Activity $\bar{A}^p_{it}$	0.1866	0.0538	3.47	0.001	0.0811	0.2922
Ego female / Friends male<br>Friends' Activity $\bar{A}^p_{it}$	-0.0272	0.0669	-0.61	0.484	-0.1085	0.0540

Total number of observations N=11,253,533. The Kleibergen-Paap rk LM statistic is 1739 (P = 0.0000) suggesting the regression is not underidentied. The Cragg-Donald Wald F statistics is 869 which exceeds the critical thresholds suggested by Stock and Yogo to ensure the instruments are not weak. The Wu-Hausmann F statistics is 232 (P=0.0000) which suggests that the friend's activity is endogenous.

Supplementary Table 12: Results of the second stage of the interaction model in Supplementary Equation 8. The same results are graphically displayed in Figure 2D of the main manuscript.

Identification Model: **Supplementary Equation 9**
Fitness Indicator : **Daily Distance [km]**
Instruments: **Interactions between 1.$R_t^{(0)}$ and $sg_{ij} \in \{0, 1\}$**

second stage results

Ego' s Activity A_{it}

	coefficient	std. error	t-value	$P > \|t\|$	95% conf. interval low	high
Ego - Friends *same-gender* Friends' Activity $\bar{A}_{it}^{p}$	0.2932	0.0373	7.85	0.000	0.2200	0.3664
Ego - Friends *cross-gender* Friends' Activity $\bar{A}_{it}^{p}$	0.0754	0.0376	2.01	0.045	0.0018	0.1490

Total number of observations N=11,253,533. The Kleibergen-Paap rk LM statistic is 1555 (P = 0.0000) suggesting the regression is not underidentied. The Cragg-Donald Wald F statistics is 786 which exceeds the critical thresholds suggested by Stock and Yogo to ensure the instruments are not weak. The Wu-Hausmann F statistics is 213 (P=0.0000) which suggests that the friend's activity is endogenous.

Supplementary Table 13: Results of the second stage of the interaction model in Supplementary Equation 9. The same results are graphically displayed in the inset of Figure 2D in the main manuscript.

Identification Model: **Supplementary Equation 10**

Fitness Indicator: **Distance [km]**

Instruments: **1.** $R_t^{(0)}$

first stage					95% conf. interval	
# of running friends (*#FR*)	coefficient	std. error	t-value	$P > \|t\|$	low	high
Instrument 1	0.0053	0.0001	38.72	0.000	0.0050	0.0056
second stage					95% conf. interval	
Ego' s Activity A_{it}	coefficient	std. error	t-value	$P > \|t\|$	low	high
# of running friends (*#FR*)	1.0844	0.1277	8.49	0.000	0.8341	1.3347

Total number of observations N=10,472,115. The Kleibergen-Paap rk LM statistic is 2100 (P = 0.0000) suggesting the regression is not underidentied. The Cragg-Donald Wald F statistics is 1100 which exceeds the critical thresholds suggested by Stock and Yogo to ensure the instruments are not weak. The Wu-Hausmann F statistics is 392 (P=0.0000) which suggests that the *FR* is endogenous.

Fitness Indicator.: **Duration [min]**

Instruments: **1.** $R_t^{(0)}$

first stage					95% conf. interval	
# of running friends (*#FR*)	coefficient	std. error	t-value	$P > \|t\|$	low	high
Instrument 1	0.0053	0.0001	38.76	0.000	0.0050	0.0056
second stage					95% conf. interval	
Ego' s Activity A_{it}	coefficient	std. error	t-value	$P > \|t\|$	low	high
# of running friends (*#FR*)	7.929	1.066	7.44	0.000	5.839	10.018

Total number of observations N=5,698,030. The Kleibergen-Paap rk LM statistic is 2200 (P = 0.0000) suggesting the regression is not underidentied The Cragg-Donald Wald F statistics is 1029 which exceeds the critical thresholds suggested by Stock and Yogo to ensure the instruments are not weak. The Wu-Hausmann F statistics is 420 (P=0.0000) which suggests that the *#FR* is endogenous.

Supplementary Table 14: The effect of the number of running friends on the Ego's activity. The results for the "Distance" are graphically displayed in figure 3A of the main manuscript.

Identification Method: **Ego Level 2SLS – Ego ID Fixed Effects**
Endogenous Variables: **# of running friends *FR* and (# of running friends)^2 *(FR)*^{2}**
Instruments: **1.** $R_t^{(7)}$ **2.** $\Theta_t^{(2)}$

Fitness Ind.: **Distance [km]**

second stage					95% conf. interval	
Ego' s Activity A_{it}	coefficient	std. error	t-value	$P > \|t\|$	low	high
# of running friends *FR*	1.333	0.0652	20.42	0.000	1.205	1.461
(# of running friends)^2 *(FR)*^{2}	-0.0077	0.0012	-6.31	0.000	-0.0101	-0.0053

Total number of observations N=10,674,361. The Kleibergen-Paap rk LM statistic is 15000 (P = 0.0000) suggesting the regression is not underidentied The Cragg-Donald Wald F statistics is 4938 which exceeds the critical thresholds suggested by Stock and Yogo to ensure the instruments are not weak. The Wu-Hausmann F statistics is 577 (P=0.0000) which suggests that the friend's activity is endogenous.

Fitness Ind.: **Duration [min]**

second stage					95% conf. interval	
Ego' s Activity A_{it}	coefficient	std. error	t-value	$P > \|t\|$	low	high
# of running friends *FR*	6.040	0.3222	18.74	0.0000	5.409	6.672
(# of running friends)^2 *(FR)*^{2}	-0.0179	0.0060	-2.98	0.003	-0.0296	-0.0061

Total number of observations N=10,645,722. The Kleibergen-Paap rk LM statistic is 14000 (P = 0.0000) suggesting the regression is not underidentied The Cragg-Donald Wald F statistics is 4835 which exceeds the critical thresholds suggested by Stock and Yogo to ensure the instruments are not weak. The Wu-Hausmann F statistics is 592 (P=0.0000) which suggests that the friend's activity is endogenous.

Supplementary Table 15: The effect of the number of running friends (as a single variable) and its square on the Ego's activity.

Identification Model: **Supplementary Equation 12**

Fitness Ind.: **Distance [km]**

Instruments: **1.** $R_t^{(0)}$

first stage					95% conf. interval	
# of running comp. (*#CR*)	coefficient	std. error	t-value	$P > \|t\|$	low	high
Instrument 1	0.0071	0.0001	68.08	0.000	0.0069	0.0073

second stage					95% conf. interval	
Ego' s Activity A_{it}	coefficient	std. error	t-value	$P > \|t\|$	low	high
# of running comp. (*#CR*)	0.7982	0.0926	8.61	0.000	0.6165	0.9798

Total number of observations N=10,472,115. The Kleibergen-Paap rk LM statistic is 6971 (P = 0.0000) suggesting the regression is not underidentied. The Cragg-Donald Wald F statistics is 4490 which exceeds the critical thresholds suggested by Stock and Yogo to ensure the instruments are not weak. The Wu-Hausmann F statistics is 323 (P=0.0000) which suggests that the *#CR* is endogenous.

Fitness Ind.: **Duration [min]**

Instruments: **1.** $R_t^{(0)}$

first stage					95% conf. interval	
# of running comp. (*#CR*)	coefficient	std. error	t-value	$P > \|t\|$	low	high
Instrument 1	0.0072	0.0002	68.11	0.000	0.0070	0.0074

second stage					95% conf. interval	
Ego' s Activity A_{it}	coefficient	std. error	t-value	$P > \|t\|$	low	high
# of running comp. (*#CR*)	5.8397	0.7768	7.52	0.000	4.317	7.362

Total number of observations N=10,472,098. The Kleibergen-Paap rk LM statistic is 6813 (P = 0.0000) suggesting the regression is not underidentied The Cragg-Donald Wald F statistics is 4639 which exceeds the critical thresholds suggested by Stock and Yogo to ensure the instruments are not weak. The Wu-Hausmann F statistics is 231 (P=0.0000) which suggests that the *#CR* is endogenous.

Supplementary Table 16: The effect of the number of running *connected components* on the Ego's activity. The results for the "Distance" are graphically displayed in figure 3A of the main manuscript. The results for the "Duration" are graphically displayed in Supplementary Figure 19.

Identification Model: **Supplementary Equation 13**

Fitness Ind.: **Distance [km]**

Instruments: **1.** $R_t^{(3)}$ **2.** $\Theta_t^{(4)}$

first stage					95% conf. interval	
# of running friends *(#FR)*	coefficient	std. error	t-value	$P > \|t\|$	low	high
Instrument 1	0.0059	0.0001	42.67	0.000	0.0056	0.0062
Instrument 2	-0.0105	0.0001	-133.90	0.000	-0.0107	-0.0103
# of running comp. *(#CR)*	coefficient	std. error	t-value	$P > \|t\|$	low	high
Instrument 1	0.0077	0.0001	72.91	0.000	-0.0075	-0.0079
Instrument 2	-0.0058	0.0001	-97.12	0.000	-0.060	-0.0057
second stage					95% conf. interval	
Ego' s Activity A_{it}	coefficient	std. error	t-value	$P > \|t\|$	low	high
# of running friends*(#FR)*	-1.288	0.1053	-12.23	0.000	-1.495	-1.082
# of running comp. *(#CR)*	1.749	0.1587	11.02	0.000	1.4386	2.060

Total number of observations N=9,920,398. The Kleibergen-Paap rk LM statistic is 888 (P = 0.0000) suggesting the regression is not underidentied. The Cragg-Donald Wald F statistics is 444 which exceeds the critical thresholds suggested by Stock and Yogo to ensure the instruments are not weak. The Wu-Hausmann F statistics is 114 (P=0.0000) which suggests that the *#FR* and *#CR* are endogenous.

Fitness Ind.: **Duration [min]**

Instruments: **1.** $R_t^{(3)}$ **2.** $\Theta_t^{(4)}$

first stage					95% conf. interval	
# of running friends *(#FR)*	coefficient	std. error	t-value	$P > \|t\|$	low	high
Instrument 1	0.0059	0.0001	42.72	0.000	0.0056	0.0061
Instrument 2	-0.0105	0.0001	-133.87	0.000	-0.0107	-0.0103
# of running comp. *(#CR)*	coefficient	std. error	t-value	$P > \|t\|$	low	high
Instrument 1	0.0077	0.0001	-72.95	0.000	0.0075	0.0079
Instrument 2	-0.0058	0.0001	-97.09	0.000	-0.0060	-0.0057
second stage					95% conf. interval	
Ego' s Activity A_{it}	coefficient	std. error	t-value	$P > \|t\|$	low	high
# of running friends *(#FR)*	-10.77	0.8844	-12.17	0.000	-12.50	-9.032
# of running comp. *(#CR)*	14.19	1.3329	10.65	0.000	11.57	16.80

Total number of observations N=9,920,382. The Kleibergen-Paap rk LM statistic is 901 (P = 0.0000) suggesting the regression is not underidentied The Cragg-Donald Wald F statistics is 550 which exceeds the critical thresholds suggested by Stock and Yogo to ensure the instruments are not weak. The Wu-Hausmann F statistics is 92 (P=0.0000) which suggests that the *#FR* and *#CR* are endogenous.

Supplementary Table 17: The effect of the number of running friends (as a single variable) and the number of running connected components on the Ego's activity. Results for the "Distance" display in the Figure 3B of the main manuscript while the result for the running "duration" display in Supplementary Figure 20.

Identification Model: **Supplementary Equation 14**
Instruments: **Interactions between 1.**$R_t^{(7)}$**, 2.**$\Theta_t^{(2)}$ **and** $e_{ij} \in \{0, 1\}$

Fitness Ind.: **Distance [km]**

second stage results

Ego' s Activity A_{it}

	coefficient	std. error	t-value	$P > \|t\|$	95% conf. interval low	high
Ego - Friends *(embedded)* Friends' Activity $\bar{A}_{it}^p$	0.5616	0.1620	4.04	0.000	0.2539	0.8593
Ego - Friends *(non-embedded)* Friends' Activity $\bar{A}_{it}^p$	0.1777	0.02189	8.12	0.000	0.1349	0.2206

Total number of observations N=1,500,123/10,733,162 (embedded/nonembedded). The Kleibergen-Paap rk LM statistic is 980/1435 (P = 0.0000) suggesting the regression is not underidentied. The Cragg-Donald Wald F statistics is 234/786 which exceeds the critical thresholds suggested by Stock and Yogo to ensure the instruments are not weak. The Wu-Hausmann F statistics is 39/84 (P=0.0000) which suggests that the friend's activity is endogenous. The Hansen overidentification restriction test gives p-value equal to P=0.1321/0.2123 that fails to reject the null hypothesis for valid instruments.

Fitness Ind.: **Duration [min]**

second stage results

Ego' s Activity A_{it}

	coefficient	std. error	t-value	$P > \|t\|$	95% conf. interval low	high
Ego - Friends *(embedded)* Friends' Activity $\bar{A}_{it}^p$	0.3525	0.1688	2.31	0.006	0.1083	0.6234
Ego - Friends *(non-embedded)* Friends' Activity $\bar{A}_{it}^p$	0.1993	0.0244	8.17	0.000	0.1515	0.2472

Total number of observations N=1,497,322/10,733,100 (embedded/nonembedded). The Kleibergen-Paap rk LM statistic is 711/1682 (P = 0.0000) suggesting the regression is not underidentied. The Cragg-Donald Wald F statistics is 287/841 which exceeds the critical thresholds suggested by Stock and Yogo to ensure the instruments are not weak. The Wu-Hausmann F statistics is 29/66 (P=0.0000) which suggests that the friend's activity is endogenous. The Hansen overidentification restriction test gives p-value equal to P=0.1349/0.2088 that fails to reject the null hypothesis for valid instruments.

Supplementary Table 18: Results of the second stage of the interaction model in Supplementary Equation 14. The same results are graphically displayed in the inset of Figure 3C in the main manuscript for the running distance and in the Supplementary Figure 21 for the running duration.

Exogeneity Test
Identification Model: **Supplementary Equation 15**

Fitness Ind.: **Distance [km]**	Instruments: $\mathbf{1.}R_t^{(7)}$, $\mathbf{2.}\Theta_t^{(2)}$					
					95% conf. interval	
Ego' s Activity A_{it}	coefficient	std. error	t-value	$P>\|t\|$	low	high
Instrument 1	0.0007	0.0008	1.01	0.171	-0.0002	0.0016
Instrument 2	0.0004	0.0005	0.89	0.371	-0.0005	0.0014

Total number of observations N=9,588,231.

Fitness Ind.: **Pace [km/min]**	Instruments: $\mathbf{1.}R_t^{(3)}$, $\mathbf{2.}\Theta_t^{(3)}$					
					95% conf. interval	
Ego' s Activity A_{it}	coefficient	std. error	t-value	$P>\|t\|$	low	high
Instrument 1	0.0001	0.0000	1.29	0.109	-0.0000	0.0001
Instrument 2	-0.0000	0.0000	-0.13	0.900	-0.0000	0.0000

Total number of observations N=9,592,634.

Fitness Ind.: **Duration [min]**	Instruments: $\mathbf{1.}R_t^{(7)}$, $\mathbf{2.}\Theta_t^{(3)}$					
					95% conf. interval	
Ego' s Activity A_{it}	coefficient	std. error	t-value	$P>\|t\|$	low	high
Instrument 1	0.0158	0.0196	1.43	0.090	-0.0055	0.0356
Instrument 2	0.0015	0.0036	0.43	0.670	-0.0055	0.0086

Total number of observations N=9,596,778.

Fitness Ind.: **calories [cal]**	Instruments: $\mathbf{1.}R_t^{(7)}$, $\mathbf{2.}\Theta_t^{(3)}$					
					95% conf. interval	
Ego' s Activity A_{it}	coefficient	std. error	t-value	$P>\|t\|$	low	high
Instrument 1	0.0222	0.0300	0.88	0.136	-0.0277	0.0700
Instrument 2	0.01534	0.0324	0.47	0.636	-0.0481	0.0788

Total number of observations N=9,596,404.

Supplementary Table 19: Instrument exogeneity test using the structural model of Supplementary Equation 15.

Identification Model: **Supplementary Equation 2**
Fitness Indicator : **Daily Distance [km]**
Instruments: **1.** R_{ft}**, 2.** Θ_{ft}**.**

A_{it} Vs. $\bar{A}^p_{it}$

first stage					95% conf. interval	
Friends' Av. Activity $\bar{A}^p_{it}$	coefficient	std. error	t-value	$P > \|t\|$	low	high
Instrument 1	-0.0858	0.0036	-23.81	0.000	-0.0928	-0.0787
Instrument 2	-0.0473	0.0030	-15.88	0.000	-0.0532	-0.0415
second stage					95% conf. interval	
Ego' s Activity A_{it}	coefficient	std. error	t-value	$P > \|t\|$	low	high
Friends' Av. Activity $\bar{A}^p_{it}$	0.3140	0.0343	7.70	0.000	0.2468	0.3912

Total number of observations N=9,560,804. The Kleibergen-Paap rk LM statistic is 771 (P = 0.0000) suggesting the regression is not underidentied. The Cragg-Donald Wald F statistics is 386 which exceeds the critical thresholds suggested by Stock and Yogo to ensure the instruments are not weak. The Wu-Hausmann F statistics is 59 (P=0.0000) which suggests that the friend's activity is endogenous. The Hansen overidentification restriction test gives p-value equal to P=0.2517 that fails to reject the null hypothesis for valid instruments.

$A_{i,t+1}$ Vs. $\bar{A}^p_{it}$

first stage					95% conf. interval	
Friends' Av. Activity $\bar{A}^p_{it}$	coefficient	std. error	t-value	$P > \|t\|$	low	high
Instrument 1	-0.0795	0.0036	-22.14	0.000	-0.0865	-0.0724
Instrument 2	-0.0413	0.0030	-13.94	0.000	-0.0471	-0.0355
second stage					95% conf. interval	
Ego' s Activity $A_{i,t+1}$	coefficient	std. error	t-value	$P > \|t\|$	low	high
Friends' Av. Activity $\bar{A}^p_{it}$	0.2092	0.0329	5.44	0.000	0.1447	0.2738

Total number of observations N=10,698,170. The Kleibergen-Paap rk LM statistic is 644 (P = 0.0000) suggesting the regression is not underidentied. The Cragg-Donald Wald F statistics is 298 which exceeds the critical thresholds suggested by Stock and Yogo to ensure the instruments are not weak. The Wu-Hausmann F statistics is 47 (P=0.000) which suggests that the friend's activity is endogenous. The Hansen overidentification restriction test gives p-value equal to P=0.2177 that fails to reject the null hypothesis for valid instruments.

$A_{i,t+2}$ Vs. $\bar{A}^p_{it}$

first stage					95% conf. interval	
Friends' Av. Activity $\bar{A}^p_{it}$	coefficient	std. error	t-value	$P > \|t\|$	low	high
Instrument 1	-0.0752	0.0036	-20.99	0.000	-0.0822	-0.0682
Instrument 2	-0.0382	0.0030	-12.86	0.000	-0.0441	-0.0324
second stage					95% conf. interval	
Ego' s Activity $A_{i,t+2}$	coefficient	std. error	t-value	$P > \|t\|$	low	high
Friends' Av. Activity $\bar{A}^p_{it}$	0.1525	0.0337	3.93	0.000	0.0864	0.2187

Total number of observations N=12,695,085. The Kleibergen-Paap rk LM statistic is 574 (P = 0.0000) suggesting the regression is not underidentied. The Cragg-Donald Wald F statistics is 266 which exceeds the critical thresholds suggested by Stock and Yogo to ensure the instruments are not weak. The Wu-Hausmann F statistics is 29 (P=0.000) which suggests that the friend's activity is endogenous. The Hansen overidentification restriction test gives p-value equal to P=0.1788 that fails to reject the null hypothesis for valid instruments.

Supplementary Table 20: Ego Influence Identification Results (Ego ID fixed effects) – Daily Running Distance. Instruments are designed as described in "Alternative Instrument Design" in Supplementary Note 4. Results are displaying in Supplementary Figure 23 (top-left panel).

Identification Model: **Supplementary Equation 2**
Fitness Indicator : **Daily Pace [km/min]**
Instruments: **1.** R_{ft}**, 2.** Θ_{ft}**.**

A_{it} Vs. $\bar{A}_{it}^{p}$

first stage					95% conf. interval	
Friends' Av. Activity $\bar{A}_{it}^{p}$	coefficient	std. error	t-value	$P > \|t\|$	low	high
Instrument 1	-0.0012	0.0000	-27.71	0.000	-0.0013	-0.0011
Instrument 2	-0.0003	0.0000	-7.17	0.000	-0.0004	-0.0002
second stage					95% conf. interval	
Ego' s Activity A_{it}	coefficient	std. error	t-value	$P > \|t\|$	low	high
Friends' Av. Activity $\bar{A}_{it}^{p}$	0.3390	0.0338	8.88	0.000	0.2726	0.4053

Total number of observations N=9,700,135. The Kleibergen-Paap rk LM statistic is 644 (P = 0.0000) suggesting the regression is not underidentied. The Cragg-Donald Wald F statistics is 305 which exceeds the critical thresholds suggested by Stock and Yogo to ensure the instruments are not weak. The Wu-Hausmann F statistics is 72 (P=0.0000) which suggests that the friend's activity is endogenous. The Hansen overidentification restriction test gives p-value equal to P=0.2145 that fails to reject the null hypothesis for valid instruments.

$A_{i,t+1}$ Vs. $\bar{A}_{it}^{p}$

first stage					95% conf. interval	
Friends' Av. Activity $\bar{A}_{it}^{p}$	coefficient	std. error	t-value	$P > \|t\|$	low	high
Instrument 1	-0.0011	0.0000	-23.46	0.000	-0.0012	-0.0010
Instrument 2	-0.0002	0.0000	-5.92	0.000	-0.0003	-0.0001
second stage					95% conf. interval	
Ego' s Activity $A_{i,t+1}$	coefficient	std. error	t-value	$P > \|t\|$	low	high
Friends' Av. Activity $\bar{A}_{it}^{p}$	0.1434	0.0317	3.88	0.000	0.0813	0.2055

Total number of observations N=10,698,170. The Kleibergen-Paap rk LM statistic is 836 (P = 0.0000) suggesting the regression is not underidentied. The Cragg-Donald Wald F statistics is 230 which exceeds the critical thresholds suggested by Stock and Yogo to ensure the instruments are not weak. The Wu-Hausmann F statistics is 57 (P=0.000) which suggests that the friend's activity is endogenous. The Hansen overidentification restriction test gives p-value equal to P=0.1876 that fails to reject the null hypothesis for valid instruments.

$A_{i,t+2}$ Vs. $\bar{A}_{it}^{p}$

first stage					95% conf. interval	
Friends' Av. Activity $\bar{A}_{it}^{p}$	coefficient	std. error	t-value	$P > \|t\|$	low	high
Instrument 1	-0.0010	0.0000	-21.34	0.000	-0.0010	-0.0009
Instrument 2	-0.0001	0.0000	-4.51	0.000	-0.0002	-0.0001
second stage					95% conf. interval	
Ego' s Activity $A_{i,t+2}$	coefficient	std. error	t-value	$P > \|t\|$	low	high
Friends' Av. Activity $\bar{A}_{it}^{p}$	0.072	0.03199	2.17	0.000	0.012	0.1358

Total number of observations N=12,707,505. The Kleibergen-Paap rk LM statistic is 458 (P = 0.0000) suggesting the regression is not underidentied. The Cragg-Donald Wald F statistics is 216 which exceeds the critical thresholds suggested by Stock and Yogo to ensure the instruments are not weak. The Wu-Hausmann F statistics is 39 (P=0.000) which suggests that the friend's activity is endogenous. The Hansen overidentification restriction test gives p-value equal to P=0.1471 that fails to reject the null hypothesis for valid instruments.

Supplementary Table 21: Ego Influence Identification Results (Ego ID fixed effects) – Daily Running Pace. Instruments are designed as described in "Alternative Instrument Design" in Supplementary Note 4. Results are displaying in Supplementary Figure 23 (top-right panel).

Identification Model: **Supplementary Equation 2**
Fitness Indicator : **Daily Duration [min]**
Instruments: **1.** R_{ft}**, 2.** Θ_{ft}**.**

A_{it} Vs. $\bar{A}^p_{it}$

first stage					95% conf. interval	
Friends' Av. Activity $\bar{A}^p_{it}$	coefficient	std. error	t-value	$P > \|t\|$	low	high
Instrument 1	-0.4300	0.0169	-25.39	0.000	-0.4632	-0.3968
Instrument 2	-0.1923	0.0142	-13.52	0.000	-0.2202	-0.1645
second stage					95% conf. interval	
Ego' s Activity A_{it}	coefficient	std. error	t-value	$P > \|t\|$	low	high
Friends' Av. Activity $\bar{A}^p_{it}$	0.2950	0.0335	8.80	0.000	0.2293	0.3607

Total number of observations N=9,637,236. The Kleibergen-Paap rk LM statistic is 795 (P = 0.0000) suggesting the regression is not underidentied. The Cragg-Donald Wald F statistics is 407 which exceeds the critical thresholds suggested by Stock and Yogo to ensure the instruments are not weak. The Wu-Hausmann F statistics is 63 (P=0.0000) which suggests that the friend's activity is endogenous. The Hansen overidentification restriction test gives p-value equal to P=0.2497 that fails to reject the null hypothesis for valid instruments.

$A_{i,t+1}$ Vs. $\bar{A}^p_{it}$

first stage					95% conf. interval	
Friends' Av. Activity $\bar{A}^p_{it}$	coefficient	std. error	t-value	$P > \|t\|$	low	high
Instrument 1	-0.3775	0.0168	-22.48	0.000	-0.4104	-0.3446
Instrument 2	-0.1458	0.0140	-10.41	0.000	-0.1733	-0.1184
second stage					95% conf. interval	
Ego' s Activity $A_{i,t+1}$	coefficient	std. error	t-value	$P > \|t\|$	low	high
Friends' Av. Activity $\bar{A}^p_{it}$	0.2274	0.0338	6.73	0.000	0.1612	0.2935

Total number of observations N=10,628,895. The Kleibergen-Paap rk LM statistic is 590 (P = 0.0000) suggesting the regression is not underidentied. The Cragg-Donald Wald F statistics is 298 which exceeds the critical thresholds suggested by Stock and Yogo to ensure the instruments are not weak. The Wu-Hausmann F statistics is 51 (P=0.0000) which suggests that the friend's activity is endogenous. The Hansen overidentification restriction test gives p-value equal to P=0.2077 that fails to reject the null hypothesis for valid instruments.

$A_{i,t+2}$ Vs. $\bar{A}^p_{it}$

first stage					95% conf. interval	
Friends' Av. Activity $\bar{A}^p_{it}$	coefficient	std. error	t-value	$P > \|t\|$	low	high
Instrument 1	-0.3502	0.0168	-20.91	0.000	-0.3831	-0.3174
Instrument 2	-0.1232	0.0140	-8.79	0.000	-0.1507	-0.0957
second stage					95% conf. interval	
Ego' s Activity $A_{i,t+2}$	coefficient	std. error	t-value	$P > \|t\|$	low	high
Friends' Av. Activity $\bar{A}^p_{it}$	0.1094	0.0345	3.57	0.000	0.0417	0.1771

Total number of observations N=12,622,086. The Kleibergen-Paap rk LM statistic is 497 (P = 0.0000) suggesting the regression is not underidentied. The Cragg-Donald Wald F statistics is 255 which exceeds the critical thresholds suggested by Stock and Yogo to ensure the instruments are not weak. The Wu-Hausmann F statistics is 36 (P=0.000) which suggests that the friend's activity is endogenous. The Hansen overidentification restriction test gives p-value equal to P=0.1665 that fails to reject the null hypothesis for valid instruments.

Supplementary Table 22: Ego Influence Identification Results (Ego ID fixed effects) – Daily Running Duration. Instruments are designed as described in "Alternative Instrument Design" in Supplementary Note 4. Results are displaying in Supplementary Figure 23 (bottom-left panel).

Identification Model: **Supplementary Equation 2**
Fitness Indicator : **Daily Calories burned [cal]**
Instruments: **1.** R_{ft}**, 2.** Θ_{ft}**.**

A_{it} Vs. $\bar{A}^p_{it}$

first stage					95% conf. interval	
Friends' Av. Activity $\bar{A}^p_{it}$	coefficient	std. error	t-value	$P > \|t\|$	low	high
Instrument 1	-4.964	0.2013	-24.66	0.000	-5.359	-4.570
Instrument 2	-2.761	0.1669	-16.54	0.000	-3.088	-2.434
second stage					95% conf. interval	
Ego' s Activity A_{it}	coefficient	std. error	t-value	$P > \|t\|$	low	high
Friends' Av. Activity $\bar{A}^p_{it}$	0.2957	0.0367	8.62	0.000	0.2238	0.3676

Total number of observations N=9,739,876. The Kleibergen-Paap rk LM statistic is 834 (P = 0.0000) suggesting the regression is not underidentied. The Cragg-Donald Wald F statistics is 383 which exceeds the critical thresholds suggested by Stock and Yogo to ensure the instruments are not weak. The Wu-Hausmann F statistics is 93 (P=0.0000) which suggests that the friend's activity is endogenous. The Hansen overidentification restriction test gives p-value equal to P=0.2001 that fails to reject the null hypothesis for valid instruments.

$A_{i,t+1}$ Vs. $\bar{A}^p_{it}$

first stage					95% conf. interval	
Friends' Av. Activity $\bar{A}^p_{it}$	coefficient	std. error	t-value	$P > \|t\|$	low	high
Instrument 1	-4.583	0.2002	-22.88	0.000	-4.975	-4.190
Instrument 2	-2.390	0.1652	-14.47	0.000	-2.714	-2.066
second stage					95% conf. interval	
Ego' s Activity $A_{i,t+1}$	coefficient	std. error	t-value	$P > \|t\|$	low	high
Friends' Av. Activity $\bar{A}^p_{it}$	0.2080	0.0339	6.13	0.000	0.1415	0.2745

Total number of observations N=10,700,294. The Kleibergen-Paap rk LM statistic is 693 (P = 0.0000) suggesting the regression is not underidentied. The Cragg-Donald Wald F statistics is 318 which exceeds the critical thresholds suggested by Stock and Yogo to ensure the instruments are not weak. The Wu-Hausmann F statistics is 51 (P=0.0000) which suggests that the friend's activity is endogenous. The Hansen overidentification restriction test gives p-value equal to P=0.1743 that fails to reject the null hypothesis for valid instruments.

$A_{i,t+2}$ Vs. $A_{i,t}$

first stage					95% conf. interval	
Friends' Av. Activity $\bar{A}^p_{it}$	coefficient	std. error	t-value	$P > \|t\|$	low	high
Instrument 1	-4.362	0.2002	-21.79	0.000	-4.754	-3.970
Instrument 2	-2.215	0.1660	-13.35	0.000	-2.540	-1.890
second stage					95% conf. interval	
Ego' s Activity $A_{i,t+2}$	coefficient	std. error	t-value	$P > \|t\|$	low	high
Friends' Av. Activity $\bar{A}^p_{it}$	0.1474	0.0348	4.24	0.000	0.0793	0.2157

Total number of observations N=12,709,634. The Kleibergen-Paap rk LM statistic is 620 (P = 0.0000) suggesting the regression is not underidentied. The Cragg-Donald Wald F statistics is 284 which exceeds the critical thresholds suggested by Stock and Yogo to ensure the instruments are not weak. The Wu-Hausmann F statistics is 37 (P=0.000) which suggests that the friend's activity is endogenous. The Hansen overidentification restriction test gives p-value equal to P=0.1314 that fails to reject the null hypothesis for valid instruments.

Supplementary Table 23: Ego Influence Identification Results (Ego ID fixed effects) – Daily Calories Burned. Instruments are designed as described in "Alternative Instrument Design" in Supplementary Note 4. Results are displaying in Figure 23 (bottom-right panel).

Falsification Test 1
Identification Model: **Supplementary Equation 16** Instruments: **1.**$R_t^{(3)}$**, 2.**$\Theta_{t+60}^{(3)}$

Fitness Ind.: **Distance [km]**

first stage					95% conf. interval	
Friends' Activity $\langle A_{t+60} \rangle_j$	coefficient	std. error	t-value	$P > \|t\|$	low	high
Instrument 1	0.0118	0.0005	24.14	0.000	0.0108	0.0127
Instrument 2	0.0059	0.0002	25.20	0.000	0.0055	0.0063
second stage					95% conf. interval	
Ego' s Activity A_{it}	coefficient	std. error	t-value	$P > \|t\|$	low	high
Friends' Activity $\langle A_{t+60} \rangle_j$	0.0923	0.0680	1.36	0.175	-0.0410	0.2254

Total number of observations N=7,831,310. The Kleibergen-Paap rk LM statistic is 1517 (P = 0.0000) suggesting the regression is not underidentied. The Cragg-Donald Wald F statistics is 313 which exceeds the critical thresholds suggested by Stock and Yogo to ensure the instruments are not weak.

Fitness Ind.: **Pace [km/min]**

first stage					95% conf. interval	
Friends' Activity $\langle A_{t+60} \rangle_j$	coefficient	std. error	t-value	$P > \|t\|$	low	high
Instrument 1	0.0002	0.0000	28.02	0.000	0.0002	0.0002
Instrument 2	0.0001	0.0000	21.49	0.000	0.0001	0.0001
second stage					95% conf. interval	
Ego' s Activity A_{it}	coefficient	std. error	t-value	$P > \|t\|$	low	high
Friends' Activity $\langle A_{t+60} \rangle_j$	0.1100	0.0611	1.80	0.072	-0.0099	0.2299

Total number of observations N=7,802,150. The Kleibergen-Paap rk LM statistic is 1510 (P = 0.0000) suggesting the regression is not underidentied. The Cragg-Donald Wald F statistics is 323 which exceeds the critical thresholds suggested by Stock and Yogo to ensure the instruments are not weak.

Fitness Ind.: **Duration [min]**

first stage					95% conf. interval	
Friends' Activity $\langle A_{t+60} \rangle_j$	coefficient	std. error	t-value	$P > \|t\|$	low	high
Instrument 1	0.0802	0.0040	19.99	0.000	0.0724	0.0881
Instrument 2	0.0471	0.0018	24.98	0.000	0.04340	0.0508
second stage					95% conf. interval	
Ego' s Activity A_{it}	coefficient	std. error	t-value	$P > \|t\|$	low	high
Friends' Activity $\langle A_{t+60} \rangle_j$	0.0234	0.0767	0.31	0.760	-0.1269	0.1738

Total number of observations N=7,831,298. The Kleibergen-Paap rk LM statistic is 1246 (P = 0.0000) suggesting the regression is not underidentied. The Cragg-Donald Wald F statistics is 244 which exceeds the critical thresholds suggested by Stock and Yogo to ensure the instruments are not weak.

Fitness Ind.: **calories [cal]**

first stage					95% conf. interval	
Friends' Activity $\langle A_{t+60} \rangle_j$	coefficient	std. error	t-value	$P > \|t\|$	low	high
Instrument 1	0.8778	0.0367	23.90	0.000	0.8058	0.9497
Instrument 2	0.4509	0.0179	25.21	0.000	0.4159	0.4860
second stage					95% conf. interval	
Ego' s Activity A_{it}	coefficient	std. error	t-value	$P > \|t\|$	low	high
Friends' Activity $\langle A_{t+60} \rangle_j$	0.1224	0.0689	1.78	0.076	-0.0127	0.2575

Total number of observations N=7,831,007. The Kleibergen-Paap rk LM statistic is 1523 (P = 0.0000) suggesting the regression is not underidentied. The Cragg-Donald Wald F statistics is 311 which exceeds the critical thresholds suggested by Stock and Yogo to ensure the instruments are not weak.

Supplementary Table 24: Ego Influence Identification Results (Ego ID fixed effects) for the Falsification Test 1 described in Supplementary Equation 16.

Falsification Test 2 / **Realization 1**
Identification Model: **Supplementary Equation 2 with manipulated social network**

Fitness Ind.: **Distance [km]** Instruments: **1.**$R_t^{(7)}$**, 2.**$\Theta_t^{(2)}$

first stage					95% conf. interval	
Friends' Activity $\bar{A}_{it}^p$	coefficient	std. error	t-value	$P > \|t\|$	low	high
Instrument 1	0.0147	0.0012	12.25	0.000	0.0123699	0.0170
Instrument 2	0.0205	0.0011	18.48	0.000	0.0183438	0.02269
second stage					95% conf. interval	
Ego' s Activity A_{it}	coefficient	std. error	t-value	$P > \|t\|$	low	high
Friends' Activity $\bar{A}_{it}^p$	0.0164	0.0484	0.34	0.735	-0.0784	0.1112

Total number of observations N=6,998,292. The Kleibergen-Paap rk LM statistic is 683 (P = 0.0000) suggesting the regression is not underidentied.
The Cragg-Donald Wald F statistics is 291 which exceeds the critical thresholds suggested by Stock and Yogo to ensure the instruments are not weak.

Fitness Ind.: **Pace [km/min]** Instruments: **1.**$R_t^{(3)}$**, 2.**$\Theta_t^{(3)}$

first stage					95% conf. interval	
Friends' Activity $\bar{A}_{it}^p$	coefficient	std. error	t-value	$P > \|t\|$	low	high
Instrument 1	0.0003	0.0000	15.02	0.000	0.0003	0.0003
Instrument 2	0.0004	0.0000	24.44	0.000	0.0004	0.0005
second stage					95% conf. interval	
Ego' s Activity A_{it}	coefficient	std. error	t-value	$P > \|t\|$	low	high
Friends' Activity $\bar{A}_{it}^p$	0.0360	0.0378	0.95	0.342	-0.0382	0.1102

Total number of observations N=7,802,150. The Kleibergen-Paap rk LM statistic is 1012 (P = 0.0000) suggesting the regression is not underidentied.
The Cragg-Donald Wald F statistics is 459 which exceeds the critical thresholds suggested by Stock and Yogo to ensure the instruments are not weak.

Fitness Ind.: **Duration [min]** Instruments: **1.**$R_t^{(7)}$**, 2.**$\Theta_t^{(3)}$

first stage					95% conf. interval	
Friends' Activity $\bar{A}_{it}^p$	coefficient	std. error	t-value	$P > \|t\|$	low	high
Instrument 1	0.10123	0.0098	10.27	0.000	0.0819	0.1205
Instrument 2	0.1559	0.0089	17.36	0.000	0.1383	0.1735
second stage					95% conf. interval	
Ego' s Activity A_{it}	coefficient	std. error	t-value	$P > \|t\|$	low	high
Friends' Activity $\bar{A}_{it}^p$	0.0480	0.0509	0.94	0.345	-0.05178	0.1479

Total number of observations N=6,998,256. The Kleibergen-Paap rk LM statistic is 505 (P = 0.0000) suggesting the regression is not underidentied.
The Cragg-Donald Wald F statistics is 227 which exceeds the critical thresholds suggested by Stock and Yogo to ensure the instruments are not weak.

Fitness Ind.: **calories [cal]** Instruments: **1.**$R_t^{(7)}$**, 2.**$\Theta_t^{(3)}$

first stage					95% conf. interval	
Friends' Activity $\bar{A}_{it}^p$	coefficient	std. error	t-value	$P > \|t\|$	low	high
Instrument 1	1.067	0.0898	11.87	0.000	0.8906	1.2422
Instrument 2	1.609	0.08197	19.63	0.000	1.448	1.769
second stage					95% conf. interval	
Ego' s Activity A_{it}	coefficient	std. error	t-value	$P > \|t\|$	low	high
Friends' Activity $\bar{A}_{it}^p$	0.0881	0.0458	1.92	0.055	-0.0017	0.1781

Total number of observations N=6,998,031. The Kleibergen-Paap rk LM statistic is 601 (P = 0.0000) suggesting the regression is not underidentied.
The Cragg-Donald Wald F statistics is 300 which exceeds the critical thresholds suggested by Stock and Yogo to ensure the instruments are not weak.

Supplementary Table 25: Ego Influence Identification Results (Ego ID fixed effects) for the 1st realization of the Falsification Test 2 using the structural model of Supplementary Equation 2 with randomly manipulated social network.

Falsification Test 2 / **Realization 2**
Identification Model: **Supplementary Equation 2 with manipulated social network**

Fitness Ind.: **Distance [km]** Instruments: **1.**$R_t^{(7)}$**, 2.**$\Theta_t^{(2)}$

first stage					95% conf. interval	
Friends' Activity $\bar{A}_{it}^p$	coefficient	std. error	t-value	$P > \|t\|$	low	high
Instrument 1	0.0126	0.0012	10.49	0.000	0.0102	0.0149
Instrument 2	0.0212	0.0010	19.31	0.000	0.0190	0.02336
second stage					95% conf. interval	
Ego' s Activity A_{it}	coefficient	std. error	t-value	$P > \|t\|$	low	high
Friends' Activity $\bar{A}_{it}^p$	0.0389	0.0449	0.87	0.386	-0.0490	0.1270

Total number of observations N=6,947,017. The Kleibergen-Paap rk LM statistic is 619 (P = 0.0000) suggesting the regression is not underidentied. The Cragg-Donald Wald F statistics is 277 which exceeds the critical thresholds suggested by Stock and Yogo to ensure the instruments are not weak.

Fitness Ind.: **Pace [km/min]** Instruments: **1.**$R_t^{(3)}$**, 2.**$\Theta_t^{(3)}$

first stage					95% conf. interval	
Friends' Activity $\bar{A}_{it}^p$	coefficient	std. error	t-value	$P > \|t\|$	low	high
Instrument 1	0.0003	0.0000	17.48	0.000	0.0002	0.0003
Instrument 2	0.0004	0.0000	22.92	0.000	0.0003	0.0004
second stage					95% conf. interval	
Ego' s Activity A_{it}	coefficient	std. error	t-value	$P > \|t\|$	low	high
Friends' Activity $\bar{A}_{it}^p$	0.0669	0.0348	1.92	0.055	-0.0014	0.1353

Total number of observations N=6,910,386. The Kleibergen-Paap rk LM statistic is 1035 (P = 0.0000) suggesting the regression is not underidentied. The Cragg-Donald Wald F statistics is 469 which exceeds the critical thresholds suggested by Stock and Yogo to ensure the instruments are not weak.

Fitness Ind.: **Duration [min]** Instruments: **1.**$R_t^{(7)}$**, 2.**$\Theta_t^{(3)}$

first stage					95% conf. interval	
Friends' Activity $\bar{A}_{it}^p$	coefficient	std. error	t-value	$P > \|t\|$	low	high
Instrument 1	0.0956	0.0099	9.65	0.000	0.0762	0.1150
Instrument 2	0.1635	0.0089	18.25	0.000	0.1459	0.1811
second stage					95% conf. interval	
Ego' s Activity A_{it}	coefficient	std. error	t-value	$P > \|t\|$	low	high
Friends' Activity $\bar{A}_{it}^p$	0.0706	0.0485	1.45	0.146	-0.0245	0.1658

Total number of observations N=6,946,978. The Kleibergen-Paap rk LM statistic is 522 (P = 0.0000) suggesting the regression is not underidentied. The Cragg-Donald Wald F statistics is 236 which exceeds the critical thresholds suggested by Stock and Yogo to ensure the instruments are not weak.

Fitness Ind.: **calories [cal]** Instruments: **1.**$R_t^{(7)}$**, 2.**$\Theta_t^{(3)}$

first stage					95% conf. interval	
Friends' Activity $\bar{A}_{it}^p$	coefficient	std. error	t-value	$P > \|t\|$	low	high
Instrument 1	1.067	0.0898	11.87	0.000	0.8906	1.2422
Instrument 2	1.609	0.08197	19.63	0.000	1.448	1.769
second stage					95% conf. interval	
Ego' s Activity A_{it}	coefficient	std. error	t-value	$P > \|t\|$	low	high
Friends' Activity $\bar{A}_{it}^p$	0.0708	7 0.0446	1.59	0.113	-0.0166	0.1584

Total number of observations N=6,946,752. The Kleibergen-Paap rk LM statistic is 619 (P = 0.0000) suggesting the regression is not underidentied. The Cragg-Donald Wald F statistics is 279 which exceeds the critical thresholds suggested by Stock and Yogo to ensure the instruments are not weak.

Supplementary Table 26: Ego Influence Identification Results (Ego ID fixed effects) for the 2nd realization of the Falsification Test 2 using the structural model of Supplementary Equation 2 with randomly manipulated social network.

Falsification Test 2 / **Realization 3**
Identification Model: **Supplementary Equation 2 with manipulated social network**

Fitness Ind.: **Distance [km]** Instruments: **1.**$R_t^{(7)}$**, 2.**$\Theta_t^{(2)}$

					95% conf. interval	
first stage					95% conf. interval	
Friends' Activity $\bar{A}_{it}^p$	coefficient	std. error	t-value	$P > \|t\|$	low	high
Instrument 1	0.0145	0.0012	11.90	0.000	0.0121	0.0169
Instrument 2	0.0216	0.0011	19.44	0.000	0.0195	0.0238
second stage					95% conf. interval	
Ego' s Activity A_{it}	coefficient	std. error	t-value	$P > \|t\|$	low	high
Friends' Activity $\bar{A}_{it}^p$	0.0503	0.0422	1.19	0.232	-0.03229	0.1330

Total number of observations N=6,913,218. The Kleibergen-Paap rk LM statistic is 678 (P = 0.0000) suggesting the regression is not underidentied. The Cragg-Donald Wald F statistics is 303 which exceeds the critical thresholds suggested by Stock and Yogo to ensure the instruments are not weak.

Fitness Ind.: **Pace [km/min]** Instruments: **1.**$R_t^{(3)}$**, 2.**$\Theta_t^{(3)}$

first stage					95% conf. interval	
Friends' Activity $\bar{A}_{it}^p$	coefficient	std. error	t-value	$P > \|t\|$	low	high
Instrument 1	0.0003	0.0000	15.29	0.000	0.0003	0.0003
Instrument 2	0.0004	0.0000	22.72	0.000	0.0003	0.0004
second stage					95% conf. interval	
Ego' s Activity A_{it}	coefficient	std. error	t-value	$P > \|t\|$	low	high
Friends' Activity $\bar{A}_{it}^p$	0.0082	0.0362	0.23	0.820	-0.0628	0.0793

Total number of observations N=6,876,408. The Kleibergen-Paap rk LM statistic is 932 (P = 0.0000) suggesting the regression is not underidentied. The Cragg-Donald Wald F statistics is 423 which exceeds the critical thresholds suggested by Stock and Yogo to ensure the instruments are not weak.

Fitness Ind.: **Duration [min]** Instruments: **1.**$R_t^{(7)}$**, 2.**$\Theta_t^{(3)}$

first stage					95% conf. interval	
Friends' Activity $\bar{A}_{it}^p$	coefficient	std. error	t-value	$P > \|t\|$	low	high
Instrument 1	0.1052	0.0101	10.34	0.000	0.0852	0.1251
Instrument 2	0.1575	0.0091	17.31	0.000	0.1396	0.1753
second stage					95% conf. interval	
Ego' s Activity A_{it}	coefficient	std. error	t-value	$P > \|t\|$	low	high
Friends' Activity $\bar{A}_{it}^p$	0.0772	0.0486	1.59	0.112	-0.01808	0.1726

Total number of observations N=6,913,176. The Kleibergen-Paap rk LM statistic is 502 (P = 0.0000) suggesting the regression is not underidentied. The Cragg-Donald Wald F statistics is 288 which exceeds the critical thresholds suggested by Stock and Yogo to ensure the instruments are not weak.

Fitness Ind.: **calories [cal]** Instruments: **1.**$R_t^{(7)}$**, 2.**$\Theta_t^{(3)}$

first stage					95% conf. interval	
Friends' Activity $\bar{A}_{it}^p$	coefficient	std. error	t-value	$P > \|t\|$	low	high
Instrument 1	1.069	0.0914	11.69	0.000	0.8900	1.248
Instrument 2	1.674	0.0823	20.33	0.000	1.512	1.835
second stage					95% conf. interval	
Ego' s Activity A_{it}	coefficient	std. error	t-value	$P > \|t\|$	low	high
Friends' Activity $\bar{A}_{it}^p$	0.0495	0.0419	1.18	0.237	-0.0325	0.1317

Total number of observations N=6,912,943. The Kleibergen-Paap rk LM statistic is 690 (P = 0.0000) suggesting the regression is not underidentied. The Cragg-Donald Wald F statistics is 311 which exceeds the critical thresholds suggested by Stock and Yogo to ensure the instruments are not weak.

Supplementary Table 27: Ego Influence Identification Results (Ego ID fixed effects) for the 3rd realization of the Falsification Test 2 using the structural model of Supplementary Equation 2 with randomly manipulated social network.

Identification Model: **Supplementary Equation 2**
Fitness Indicator : **Daily Distance [km]** Instruments: **1.** $R_t^{(7)}$ **2.** $\Theta_t^{(2)}$

second stage results

Ego' s Activity A_{it}

	coefficient	std. error	t-value	$P > \|t\|$	95% conf. interval low	high
$\rho_c = 0.001$						
Friends' Activity $\bar{A}^p_{it}$	0.3424	0.0402	8.09	0.000	0.2607	0.4229

Total number of observations N=8,091,100. The Kleibergen-Paap rk LM statistic is 1144 (P = 0.0000) suggesting the regression is not underidentied. The Cragg-Donald Wald F statistics is 329 which exceeds the critical thresholds suggested by Stock and Yogo to ensure the instruments are not weak. The Wu-Hausmann F statistics is 69 (P=0.0000) which suggests that the friend's activity is endogenous. The Hansen overidentification restriction test gives p-value equal to P=0.4199 that fails to reject the null hypothesis for valid instruments.

	coefficient	std. error	t-value	$P > \|t\|$	low	high
$\rho_c = 0.010$						
Friends' Activity $\bar{A}^p_{it}$	0.3423	0.0402	8.49	0.000	0.2638	0.4208

Total number of observations N=8,990,771. The Kleibergen-Paap rk LM statistic is 1370 (P = 0.0000) suggesting the regression is not underidentied. The Cragg-Donald Wald F statistics is 335 which exceeds the critical thresholds suggested by Stock and Yogo to ensure the instruments are not weak. The Wu-Hausmann F statistics is 121 (P=0.0000) which suggests that the friend's activity is endogenous. The Hansen overidentification restriction test gives p-value equal to P=0.3387 that fails to reject the null hypothesis for valid instruments.

	coefficient	std. error	t-value	$P > \|t\|$	low	high
$\rho_c = 0.020$						
Friends' Activity $\bar{A}^p_{it}$	0.3425	0.0400	8.56	0.000	0.2640	0.4209

Total number of observations N=9,111,810. The Kleibergen-Paap rk LM statistic is 1383 (P = 0.0000) suggesting the regression is not underidentied. The Cragg-Donald Wald F statistics is 331 which exceeds the critical thresholds suggested by Stock and Yogo to ensure the instruments are not weak. The Wu-Hausmann F statistics is 71 (P=0.0000) which suggests that the friend's activity is endogenous. The Hansen overidentification restriction test gives p-value equal to P=0.2621 that fails to reject the null hypothesis for valid instruments.

	coefficient	std. error	t-value	$P > \|t\|$	low	high
$\boldsymbol{\rho_c = 0.025}$						
Friends' Activity $\bar{A}^p_{it}$	0.3425	0.0400	8.56	0.000	0.2640	0.4209

Total number of observations N=9,560,804. The Kleibergen-Paap rk LM statistic is 1392 (P = 0.0000) suggesting the regression is not underidentied. The Cragg-Donald Wald F statistics is 335 which exceeds the critical thresholds suggested by Stock and Yogo to ensure the instruments are not weak. The Wu-Hausmann F statistics is 75 (P=0.0000) which suggests that the friend's activity is endogenous. The Hansen overidentification restriction test gives p-value equal to P=0.2122 that fails to reject the null hypothesis for valid instruments.

	coefficient	std. error	t-value	$P > \|t\|$	low	high
$\rho_c = 0.030$						
Friends' Activity $\bar{A}^p_{it}$	0.3426	0.0402	8.54	0.000	0.2640	0.4211

Total number of observations N=10,105,777. The Kleibergen-Paap rk LM statistic is 1311 (P = 0.0000) suggesting the regression is not underidentied. The Cragg-Donald Wald F statistics is 333 which exceeds the critical thresholds suggested by Stock and Yogo to ensure the instruments are not weak. The Wu-Hausmann F statistics is 76 (P=0.0000) which suggests that the friend's activity is endogenous. The Hansen overidentification restriction test gives p-value equal to P=0.1829 that fails to reject the null hypothesis for valid instruments.

	coefficient	std. error	t-value	$P > \|t\|$	low	high
$\rho_c = 0.040$						
Friends' Activity $\bar{A}^p_{it}$	0.3430	0.0421	8.14	0.000	0.2610	0.4250

Total number of observations N=12,105,729. The Kleibergen-Paap rk LM statistic is 1273 (P = 0.0000) suggesting the regression is not underidentied. The Cragg-Donald Wald F statistics is 322 which exceeds the critical thresholds suggested by Stock and Yogo to ensure the instruments are not weak. The Wu-Hausmann F statistics is 68 (P=0.0000) which suggests that the friend's activity is endogenous. The Hansen overidentification restriction test gives p-value equal to P=0.1315 that fails to reject the null hypothesis for valid instruments.

	coefficient	std. error	t-value	$P > \|t\|$	low	high
$\rho_c = 0.100$						
Friends' Activity $\bar{A}^p_{it}$	0.3510	0.0429	7.99	0.000	0.2560	0.4470

Total number of observations N=14,223,129. The Kleibergen-Paap rk LM statistic is 1373 (P = 0.0000) suggesting the regression is not underidentied. The Cragg-Donald Wald F statistics is 311 which exceeds the critical thresholds suggested by Stock and Yogo to ensure the instruments are not weak. The Wu-Hausmann F statistics is 69 (P=0.0000) which suggests that the friend's activity is endogenous. The Hansen overidentification restriction test gives p-value equal to P=0.0822 that fails to reject the null hypothesis for valid instruments.

Supplementary Table 28: Results of the second stage of the model in Supplementary Equation 2 when we consider different weather correlation thresholds.

Identification Model: **Supplementary Equation 17**

	coefficient	std. error	t-value	$P > \|t\|$	95% conf. interval low	high
total num. of raining days	0.9937	0.0020	501.15	0.000	0.9898	0.9976
average daily activity	0.0433	0.0026	16.38	0.000	0.0381	0.0485
gender						
male						
female	-0.0042	0.0005	-8.02	0.000	-0.0052	-0.0032
age	-0.0002	0.0000	-16.16	0.000	-0.0002	-0.0002
height	-8.67e-07	4.07e-06	-0.21	0.831	-8.85e-06	7.12e-06
weight	-6.73e-05	1.22e-05	-5.54	0.000	-9.11e-05	-4.35e-05
country						
other country						
USA	0.0149	0.0006	23.75	0.000	0.0136	0.0161
UK	0.0197	0.0011	18.16	0.000	0.0176	0.0219
Japan	-0.0140	0.0015	-9.33	0.000	-0.0169	-0.0110
Canada	0.0085	0.0015	5.77	0.000	0.0056	0.0114
Germany	0.0141	0.0016	8.56	0.000	0.0109	0.0173
Spain	0.0399	0.0015	25.85	0.000	0.0369	0.0430
Brasil	0.0555	0.0014	39.02	0.000	0.0527	0.0583
Australia	-0.0057	0.0017	-3.35	0.001	-0.0091	-0.0023
Mexico	-0.0070	0.0012	-5.63	0.000	-0.0095	-0.0046
Netherlands	0.0163	0.0019	8.36	0.000	0.0125	0.0202
France	0.0559	0.0017	32.23	0.000	0.0525	0.0593
device						
other						
wrist device	-0.0033	0.0006	-5.16	0.000	-0.0045	-0.0020
pedometer	0.0056	0.0010	5.45	0.000	0.0036	0.0077
application 1	0.0054	0.0023	2.36	0.018	0.0009	0.0099
application 2	0.0030	0.0008	3.58	0.000	0.0013	0.0046

Supplementary Table 29: The effect of the different time independent characteristics of individuals on the fraction of runs taken during a rainy day f.
